# Gradient-based optimization for mRNA sequence design

**DOI:** 10.1093/bioinformatics/btag667

**Published:** 2026-09-10

**Authors:** Hongmin Li, Goro Terai, Takumi Otagaki, Kiyoshi Asai

**Affiliations:** Department of Computational Biology and Medical Sciences, Graduate School of Frontier Sciences, University of Tokyo, 5-1-5 Kashiwanoha, Kashiwa-shi, Chiba, 277-8561, Japan; School of Life Science and Technology, Institute of Science Tokyo, 2-12-1 Ookayama, Meguro-ku, Tokyo, 152-8550, Japan; Department of Computational Biology and Medical Sciences, Graduate School of Frontier Sciences, University of Tokyo, 5-1-5 Kashiwanoha, Kashiwa-shi, Chiba, 277-8561, Japan; AI-Empowered Life Science Initiative (ALIS), Research Organization of Information and Systems (ROIS), 4-1-1 Toranomon, Tokyo, 105-6929, Japan; Department of Computational Biology and Medical Sciences, Graduate School of Frontier Sciences, University of Tokyo, 5-1-5 Kashiwanoha, Kashiwa-shi, Chiba, 277-8561, Japan; Department of Computational Biology and Medical Sciences, Graduate School of Frontier Sciences, University of Tokyo, 5-1-5 Kashiwanoha, Kashiwa-shi, Chiba, 277-8561, Japan; School of Life Science and Technology, Institute of Science Tokyo, 2-12-1 Ookayama, Meguro-ku, Tokyo, 152-8550, Japan; AI-Empowered Life Science Initiative (ALIS), Research Organization of Information and Systems (ROIS), 4-1-1 Toranomon, Tokyo, 105-6929, Japan

## Abstract

**Motivation:**

Designing mRNA coding sequences that simultaneously optimize RNA accessibility in the translation initiation region and codon adaptation while preserving the encoded protein requires navigating a vast discrete combinatorial space. The inherently discrete nature of codon choices prevents direct application of gradient-based optimization, despite the availability of accurate deep learning predictors such as DeepRaccess for RNA accessibility prediction.

**Results:**

We present the Input Data Differentiable Designer (ID3), a unified framework for mRNA codon optimization. ID3 treats trained models as fixed differentiable functions and optimizes input data through continuous probability distributions while preserving the encoded amino acid sequence through three constraint mechanisms. The framework shows strong performance in both accessibility optimization and joint accessibility-CAI optimization across diverse protein targets. We also provide convergence analyses from the perspective of trained model input optimization.

**Availability and implementation:**

Code, datasets, and reproduction scripts are available at https://github.com/Li-Hongmin/ID3.git and archived on Zenodo (DOI: 10.5281/zenodo.18917770).

## 1 Introduction

Rational RNA sequence optimization underpins a wide range of transformative biomedical applications, from mRNA vaccines and protein replacement therapies to next-generation cancer immunotherapies (Sahin *et al*. 2014, Pardi *et al*. 2018, Sample *et al*. 2019). Yet, designing functional mRNA sequences remains a fundamental challenge in computational biology. Effective optimization must simultaneously balance multiple, often competing, biological objectives: ensuring efficient ribosome binding and translation initiation (Kozak 1999), maintaining codon usage appropriate for the host organism (Sharp and Li 1987), and preserving the encoded amino acid sequence required for correct protein function (Presnyak *et al*. 2015).

Codon optimization is particularly critical in practical applications, as synonymous codon choices can dramatically affect protein expression levels (Plotkin and Kudla 2011), mRNA stability (Presnyak *et al*. 2015), and immunogenicity (Karikó *et al*. 2005)—factors that directly influence efficacy and safety of mRNA therapeutics (Pardi *et al*. 2018). However, traditional codon optimization methods face practical challenges in multi-objective settings. Many rely on heuristic search or fixed codon-usage rules, which can become difficult to calibrate when multiple competing objectives must be balanced and when pre-trained predictive models need to be incorporated into the design loop (Gould *et al.* 2014; Linder and Seelig 2021). Moreover, these approaches are difficult to integrate with modern deep learning models that predict mRNA accessibility or stability (Wayment-Steele *et al*. 2022). A central challenge arises from the inherently discrete nature of RNA sequences: conventional gradient-based optimization techniques require continuous and differentiable objective functions (Nocedal and Wright 2006), creating a fundamental gap between powerful predictive models and practical sequence design.

The discrete-continuous optimization gap is a well-studied challenge in mathematical optimization more broadly—integer linear programming relaxation (Schrijver 1986), reinforcement learning for discrete game playing such as AlphaGo (Silver *et al*. 2016), generative flow networks for combinatorial structure generation (Bengio *et al*. 2021), and Transformer-based language models that represent discrete sequences in continuous embedding spaces through self-attention (Vaswani *et al*. 2017) have all demonstrated successful strategies. Within RNA sequence design, several approaches have partially addressed this gap. Continuous relaxation methods (Killoran *et al*. 2017) approximate discrete sequences with continuous representations but suffer from predictor pathology when models receive out-of-distribution inputs. Activation maximization with Straight-Through Estimation (Linder and Seelig 2021) enables gradient flow through discrete operations but introduces gradient bias and lacks codon-level constraints. Differentiable folding models based on partition functions (Krueger and Ward 2025) enable joint optimization of thermodynamic stability and codon adaptation index (CAI) but remain coupled to specific physics-based energy models. Most recently, probabilistic lattice methods (Dai *et al*. 2025) employ projection-based constraint handling coupled with ViennaRNA energy models, but cannot leverage pre-trained neural network predictors.

These approaches demonstrate that the discrete-continuous gap is tractable, but they involve different trade-offs. ID3 targets the combination of pre-trained neural network predictor compatibility and systematic codon constraint handling, a setting not fully addressed by prior work (Table S15, available as supplementary data at *Bioinformatics* online).

**Figure 1:**
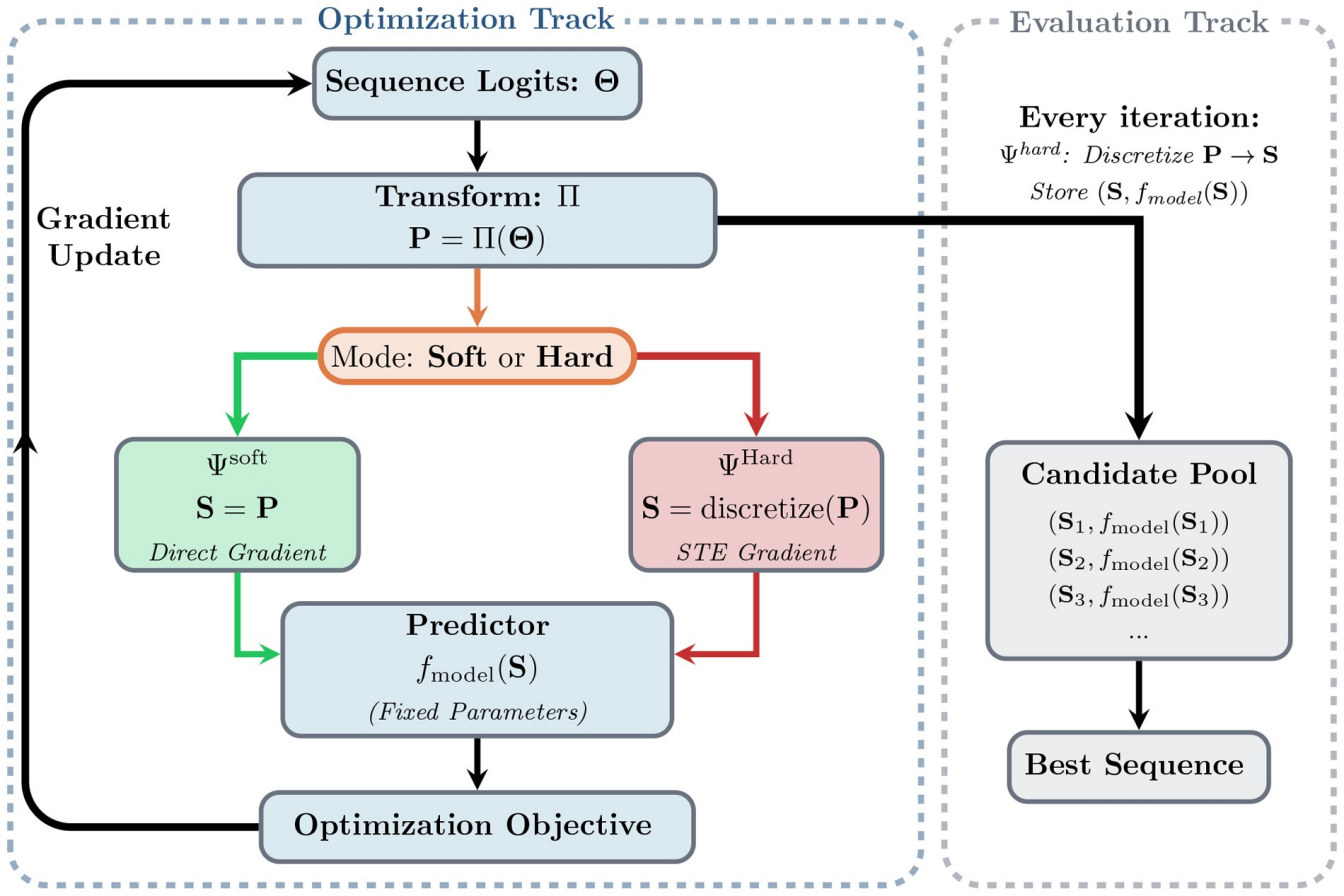
ID3 Framework Overview. The decoupled optimization-evaluation architecture showing the optimization track (Soft/Hard modes) and the independent evaluation track for biological validity assessment.

To address these limitations—including the discrete-continuous optimization gap, the lack of codon-level constraints in gradient-based optimization, and the difficulty of reusing existing predictive models—we introduce the Input Data Differentiable Designer (ID3) framework. ID3 employs a decoupled optimization-evaluation architecture, shown in Fig. 1, that separates the optimization process from candidate evaluation. The optimization track transforms sequence logits into probabilities and supports two modes: *Soft mode* uses continuous probabilities for gradient optimization, while *Hard mode* uses discretized sequences with Straight-Through Estimator (STE) gradients. The evaluation track independently collects sequence-prediction pairs at every iteration, enabling models to accept probability distributions as input while maintaining biological validity through discrete candidate evaluation.

By treating pre-trained predictors as fixed differentiable functions and optimizing over continuous probability distributions, ID3 integrates with existing deep learning models without requiring retraining. This formulation supports conditional convergence analysis under stated local assumptions and enables performance improvements in RNA design tasks such as mRNA accessibility and joint CAI optimization.

## 2 Methods

### 2.1 The mathematical framework of ID3

ID3 provides a systematic approach to mRNA sequence optimization. A discrete RNA sequence is represented by a matrix of learnable continuous parameters Θ. The Θ is transformed to a matrix of probabilities P by Π, and the P is converted to a generalized sequence S by Ψ. The objective function L is computed by a fixed pre-trained model $f_{\text{model}}(T(\Theta ))$ (T=Ψ ° Π). The core mathematical objective of the optimization is:


(1) $$\underset{\Theta }{\min}L=\underset{\Theta }{\min}f_{\text{model}}(T(\Theta )).$$

The Θ is optimized for L by the gradient-based updates ∇:


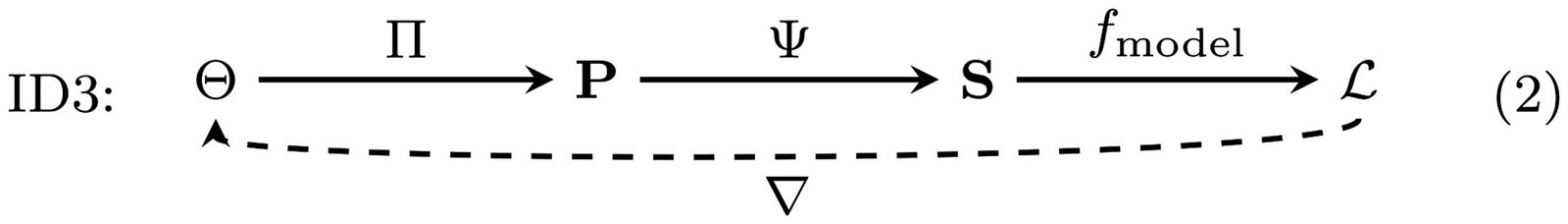


Figure 1 shows the decoupled optimization-evaluation architecture where the optimization track supports both Soft mode (continuous gradient optimization) and Hard mode (discrete sequence optimization via STE), while the evaluation track independently collects and evaluates candidates every iteration.

#### 2.1.1 Transformation functions and ID3 variants

The ID3 pipeline is implemented by two fixed algorithmic mappings (not learned neural network layers): Π converts the learnable logit matrix Θ into a codon probability distribution P, and Ψ converts P into a generalized sequence S (either a continuous probability matrix in soft mode or a discrete one-hot sequence in hard mode).

##### 2.1.1.1 Parameter-to-Probability Transformation (Π)

This transformation converts the parameter matrix Θ to a probability matrix P representing the probabilities at each position of the sequence, using Gumbel-Softmax (Gumbel 1958, Jang *et al*. 2017):


(3) $$\begin{array}{c} P^{\text{det}}=\Pi^{\text{det}\;}(\Theta )=\text{softmax}(\Theta /\sigma )\\ P^{\text{sto}}=\Pi^{\text{sto}}(\Theta )=\text{softmax}((\Theta +g)/\sigma ) \end{array}$$

where *g* is a sampled noise from Gumbel distribution, and σ is the temperature parameter. In all experiments, we fixed the relaxation temperature at σ=1.0 and did not use temperature annealing.

##### 2.1.1.2 Probability-to-Sequence Transformation (Ψ)

This transformation converts the codon probability distribution P to a generalized sequence S, supporting both soft and hard modes:


(4) $$\begin{array}{c} \Psi^{\text{soft}}(P)=P\\ \Psi^{\text{hard}}(P)=\text{discretize}(P) \end{array}$$

where discretize(P) converts probability distributions to valid discrete sequences through constraint-aware strategies.

These transformations enable two mutually exclusive optimization modes: the Soft mode, which performs stable gradient optimization over continuous probability distributions, and the Hard mode, which applies the Straight-Through Estimator (STE) (Bengio *et al*. 2013) to discrete sequences to maintain gradient flow.

The unified transformation function *T* is defined as:


(5) T=Ψ(Π(Θ))

where $\Pi \in \{\Pi^{\text{det}},\Pi^{\text{sto}}\}$ and $\Psi \in \{\Psi^{\text{soft}},\Psi^{\text{hard}}\}$, yielding four base operational modes as detailed in Table 1:

**Table 1:** ID3 base operational modes.

Mode	Parameter type	Output type	Notation
Det.Soft	Deterministic	Soft	$T^{\text{det}.\text{soft}}(\Theta )$
Det.Hard	Deterministic	Hard	$T^{\text{det}.\text{hard}}(\Theta )$
Sto.Soft	Stochastic	Soft	$T^{\text{sto}.\text{soft}}(\Theta )$
Sto.Hard	Stochastic	Hard	$T^{\text{sto}.\text{hard}}(\Theta )$

The deterministic/stochastic distinction is expressed through the choice of probability transformation $\Pi^{\text{det}/\text{sto}}$, while the soft/hard distinction occurs through the sequence transformation $\Psi^{\text{soft}/\text{hard}}$. This separation allows constraint mechanisms to focus on their core functionality without duplicating mode-specific behavior.

These four base operational modes from Table 1 combine with three constraint mechanisms to create 12 constrained variants for comprehensive biological sequence optimization.

### 2.2 Biological constraints and convergence analysis

#### 2.2.1 Codon constraint challenge in mRNA design

The central challenge in computational mRNA optimization lies in balancing two competing requirements: optimizing RNA sequence properties while maintaining the exact amino acid sequence needed for protein function. This arises from codon degeneracy—most amino acids can be encoded by multiple synonymous codons, each affecting different RNA properties yet preserving protein function.

##### 2.2.1.1 Mathematical Problem Formulation

Given a target amino acid sequence $y=[y_{1},y_{2},\ldots ,y_{M}]$ where *M* is the number of amino acids, we seek to optimize RNA properties while maintaining perfect amino acid sequence preservation:


(6) $$\begin{array}{cc} \underset{\Theta }{\min} & f_{\text{model}}(T(\Theta ))\\ s.t. & \text{AA}(T^{\text{hard}}(\Theta ))=y \end{array}$$

Here $\Theta \in R^{L\times 4}$ represents learnable RNA parameters with L=3M, $f_{\text{model}}$ is the fixed trained predictor, and AA(·) denotes the amino acid sequence function that requires discrete input. Constraints are enforced through three mechanisms: Codon Profile Constraint, Amino Matching Softmax, and Lagrangian multipliers.

#### 2.2.2 Three constraint integration methods

The ID3 framework supports amino acid constraint preservation through three distinct integration approaches:

##### 2.2.2.1 Codon Profile Constraint

This approach fundamentally redesigns the parameter space to operate directly on codon probabilities rather than nucleotide probabilities. The method achieves constraint satisfaction by construction—ensuring amino acid constraint satisfaction since invalid codons are excluded from the parameter space.


(7) $$\Theta_{\text{codon}}=\{\theta_{j,c}:c\in C(y_{j})\}$$

where each parameter corresponds only to codons that encode the target amino acid. The probability computation ensures only valid codons receive non-zero probabilities:


(8) $$P_{\text{codon}}^{\left(j\right)}=\Pi_{\text{codon}}(\theta_{j})=\text{softmax}{\left(\theta_{j,c}/\sigma \right)}_{c\in C(y_{j})}$$

This approach reduces parameter dimensionality from L×4 to $\sum_{j=1}^{M}\left|C(y_{j})\right|$, typically yielding 40–60% parameter reduction while providing the strongest constraint guarantee.

##### 2.2.2.2 Amino Matching Softmax

This method preserves the full L×4 RNA parameterization, but codon probabilities are not obtained by freely combining independent nucleotide probabilities across positions. Instead, for the *j*th amino acid position, probability is assigned only over the valid synonymous codons in $C(y_{j})$. The parameter matrix $\Theta^{\left(j\right)}\in R^{3\times 4}$ represents learnable logits for the three nucleotide positions of the codon. For each codon $c\in C(y_{j})$, the corresponding codon-specific logit is defined as the position-wise sum of the matching nucleotide logits:


(9) $$\langle \Theta^{\left(j\right)},E[c]\rangle =\sum_{p=1}^{3}\Theta_{p,c_{p}}^{\left(j\right)}$$

where $c_{p}$ denotes the nucleotide at position *p* of codon *c* and satisfies $c_{p}\in \{A,U,G,C\}$; E[c] is the one-hot encoding of codon *c*. This is *not* a Hamming-distance measure; rather, each nucleotide position contributes its own logit value to the codon-specific logit. The codon probability is then computed as:


(10) $$P_{\text{codon}}^{\left(j\right)}=\Pi_{\text{amino}}(\Theta^{\left(j\right)})=\text{softmax}\;{\left(\langle \Theta^{\left(j\right)},E[c]\rangle /\sigma \right)}_{c\in C(y_{j})}$$

where the softmax is computed only over valid codons $C(y_{j})$ for the target amino acid $y_{j}$, and σ is the relaxation temperature. Invalid codons are therefore excluded by construction and receive zero probability. If two valid codons have the same codon-specific logit, they receive the same probability, so the method can naturally represent two equally likely valid codons rather than forcing a one-hot solution. Unlike Codon Profile, this approach allows gradient flow through the full RNA parameter space while restricting the logit-to-probability mapping to valid synonymous codons.

##### 2.2.2.3 Lagrangian Multiplier

This approach adopts the classical optimization strategy of soft penalty constraints, operating on the full RNA parameter space without structural modifications. The constraint violation is measured using L2 distance to the closest valid codon encoding:


(11) $$C(P)=\frac{1}{M}\sum_{j=1}^{M}\underset{c\in C(y_{j})}{\text{min}}\|P^{\left(j\right)}-E[c]{\|}^{2}$$

The total optimization objective combines the original model loss with the constraint penalty:


(12) $$L_{\text{total}}=f_{\text{model}}(T(\Theta ))+\lambda \cdot C(P)$$

where λ is the Lagrangian multiplier that controls the penalty strength. The optimization objective becomes minimizing $L_{\text{total}}$, balancing model performance with constraint adherence. The Lagrangian multiplier is adaptively updated using subgradient method with decaying step size to ensure convergence:


(13) $$\begin{array}{c} \lambda^{\left(t+1\right)}=\text{max}(0,\lambda^{\left(t\right)}+\eta_{t}\cdot g^{\left(t\right)})\\ \eta_{t}=\eta_{0}/\sqrt{t+1} \end{array}$$

where $g^{\left(t\right)}$ is the subgradient of the constraint at iteration *t*, and $\eta_{t}$ is the decaying step size. This soft constraint approach allows temporary constraint violations during optimization while gradually enforcing compliance, providing the most flexible optimization landscape but requiring careful tuning of the penalty coefficient.

#### 2.2.3 Soft and hard mode transformation patterns

After constraint integration, we employ transformation functions to convert codon probabilities into continuous probability distributions (soft mode) or discrete sequences (hard mode). Both Codon Profile and Amino Matching constraints operate on codon probability space $P_{\text{codon}}$, applying $R_{\text{nuc}}(P_{\text{codon}})$ for soft mode and $\text{Onehot}(P_{\text{codon}})$ for hard mode, where:


(14) $$\begin{array}{c} R_{\text{nuc}}(P_{\text{codon}})=\{{\sum_{c\in C(y_{j})}{\left(P_{\text{codon}}^{\left(j\right)}\right)}_{c}\cdot E[c]\}}_{j=1}^{M}\\ \text{Onehot}(P_{\text{codon}})={\left\{E[\text{arg}\underset{c\in C(y_{j})}{\max}{\left(P_{\text{codon}}^{\left(j\right)}\right)}_{c}]\right\}}_{j=1}^{M} \end{array}$$

Their fundamental difference lies in probability generation: Codon Profile directly parameterizes codon space, while Amino Matching computes valid-codon logits from nucleotide-level parameters through similarity matching. In contrast, Lagrangian operates on RNA probability space P, using P directly in soft mode but requiring conversion to valid codon probabilities before discretization in hard mode. Table 2 summarizes these patterns.

**Table 2:** Soft and hard mode transformation patterns.

Constraint type	$\Psi^{\text{soft}}$	$\Psi^{\text{hard}}$
Codon Profile	$R_{\text{nuc}}(P_{\text{codon}})$	$\text{Onehot}(P_{\text{codon}})$
Amino Matching	$R_{\text{nuc}}(P_{\text{codon}})$	$\text{Onehot}(P_{\text{codon}})$
Lagrangian	P	$\text{Onehot}(\Pi_{\text{amino}}(P))$

#### 2.2.4 Conditional convergence analysis

We provide, to our knowledge, the first theoretical convergence analysis for sequence optimization from the trained model input optimization perspective. We show that the ID3 framework admits convergence results under explicit assumptions about predictor regularity and transformation behavior. These convergence results are conditional on local regularity assumptions of the fixed predictor and transformation maps. Because these assumptions may not hold uniformly across all predictors, variants, and input regions, we complement the theory with empirical diagnostics and interpret the guarantees as local rather than global. The theoretical framework systematically covers all 12 ID3 variants through hierarchical theorems with detailed proofs in Supplementary Section S5, available as supplementary data at *Bioinformatics* online, providing theoretical foundation for practical RNA sequence design applications.

### 2.3 Applications

To demonstrate the ID3 framework’s optimization performance and multi-objective extension capability, we conducted comprehensive evaluations on two complementary tasks: mRNA accessibility optimization and mRNA accessibility-CAI joint optimization.

#### 2.3.1 Accessibility optimization

RNA accessibility represents the thermodynamic energy required to make a region accessible, measured in kcal/mol. Lower accessibility values indicate more easily accessed regions, facilitating ribosome binding and translation initiation (Bernhart *et al*. 2011). We use DeepRaccess (Hara *et al*. 2023), a deep learning model that accelerates the classical Raccess algorithm, as the accessibility predictor $f_{\text{access}}$ in the ID3 framework.

##### 2.3.1.1 Model architecture integration

**Figure 2:**
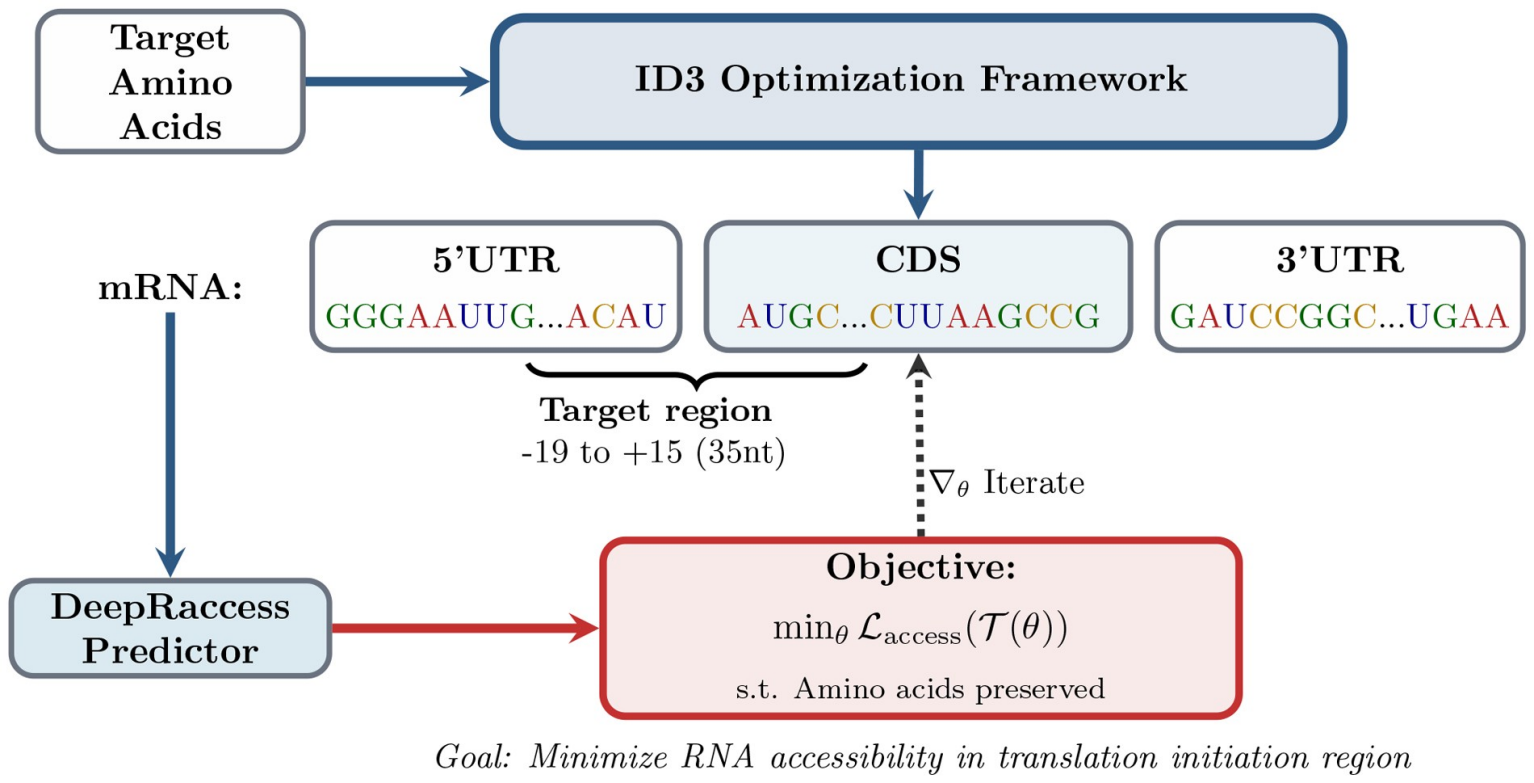
DeepRaccess Integration Framework. The ID3 optimization framework integrates with DeepRaccess predictor to minimize RNA accessibility in the translation initiation region while maintaining target amino acid sequences. The workflow shows the optimization pipeline from target amino acids through mRNA design to accessibility prediction and iterative refinement via gradient updates.

Without requiring architectural modifications or fine-tuning of trained models, the ID3 framework extends token-based neural architectures like DeepRaccess to support probabilistic sequences through a standard soft-embedding (expected-embedding) construction for continuous categorical relaxations (Jang *et al*. 2017) that enables gradient-based optimization:


(15) $${\text{softEmbedding}}^{\left(i\right)}=\sum_{b\in \{A,U,G,C\}}S_{i,b}\cdot \text{embedding}[b]$$

where $S_{i,b}\in [0,1]$ represents the probability of nucleotide *b* at position *i* from the ID3 sequence representation, and $\text{embedding}[b]\in R^{d}$ is the trained embedding vector for nucleotide *b* (Fig. 2).

##### 2.3.1.2 mRNA structure and CDS focus

The framework targets CDS optimization within the complete mRNA structure:


(16) $$\text{mRNA}=5^{\prime }\text{UTR}|\text{CDS}|3^{\prime }\text{UTR}$$

##### 2.3.1.3 Loss function design

The ribosome binding site and start codon region from position −19 to +15 relative to the ATG start codon determines translation initiation efficiency (Terai and Asai 2020). We reformulate the general constrained optimization framework (Equation (6)) for the accessibility optimization task as:


(17) Laccess(T(Θ))=faccess(T(Θ))ATG−19:ATG+15 s.t.    AA(Thard(Θ))=ytarget 

where the objective function focuses on minimizing RNA accessibility in the critical translation initiation region, and the amino acid constraint is enforced through one of the three constraint mechanisms described in Section 2.2.

#### 2.3.2 Accessibility-CAI joint optimization

The Codon Adaptation Index (CAI) (Sharp and Li 1987) measures codon usage bias relative to highly expressed genes in the target organism, reflecting the degree of adaptation to organism-specific translational machinery. To enable simultaneous optimization of RNA accessibility and CAI, we extend Equation (17) through two complementary technical modifications:

##### 2.3.2.1 CAI-aware Discretization

The base evaluation-stage CAI mechanism in all CAI experiments enhances the discretization process to consider both accessibility and CAI objectives for both soft and hard modes. The optimal sequence is found by solving:


(18) $$\begin{array}{c} \Psi_{\text{CAI}}^{\text{hard}}(P_{\text{codon}})=\text{argmax}\;P(S|P_{\text{codon}})\\ s.t.\text{CAI}(S)\geq \text{threshold} \end{array}$$

where *S* is the discrete sequence. The CAI threshold is set based on organism-specific requirements (e.g., 0.8 for *E. coli*). In the experimental tables, configurations without the + Penalty suffix use this base CAI-aware discretization mechanism alone. To solve this constrained optimization, we introduce a linear interpolation of the codon probability distribution with the target organism’s codon usage frequency vector:


(19) $$P(\gamma )=\gamma \cdot w+(1-\gamma )\cdot P_{\;\text{codon}}$$

where w is the codon usage frequency vector for the target organism, and γ∈[0,1] controls the trade-off between model probability and codon adaptation. By adjusting γ, we can find a sequence that maximizes P(S|P(γ)) while satisfying the CAI constraint. We solve this constrained optimization problem through a binary search-based method that iteratively finds the optimal sequence by balancing probability consistency and CAI requirements. The detailed algorithm is in Supplementary Section S8, available as supplementary data at *Bioinformatics* online. For the Lagrangian constraint mechanism, this approach requires first converting RNA probabilities to codon probabilities using $\Pi_{\text{amino}}$ before applying the CAI enhancement.

###### 2.3.2.2 Multi-objective Joint Loss Option

As an additional enhancement option for both soft and hard modes, CAI constraints can also be integrated through multi-objective joint loss:


(20) $$L_{\text{total}}=L_{\text{access}}+\lambda_{\text{CAI}}\cdot L_{\text{CAI}}$$

where $L_{\text{CAI}}$ measures deviation from the target CAI threshold. For probabilistic optimization with codon distributions, we compute the expected CAI as a weighted average of codon weights according to their probabilities, enabling gradient-based optimization of the CAI penalty term (see Supplementary Section S9.1, available as supplementary data at *Bioinformatics* online for formulation). The penalty coefficient $\lambda_{\text{CAI}}$ controls the relative importance of the two objectives, offering flexible multi-objective optimization.

## 3 Results

### 3.1 Computational experiment setup

We evaluated ID3 across 12 diverse proteins (64–1273 amino acids) with 20 independent runs per protein-variant combination. The dataset spans multiple functional categories and organisms (Table 3) for comprehensive framework assessment. The UTR sequences are derived from the pET-11a expression vector (Supplementary Section S10, available as supplementary data at *Bioinformatics* online).

**Table 3:** Protein dataset.

UniProt ID	Protein Name	Organism	Length (aa)
O15263	Defensin beta 4A	*H. sapiens*	64
P99999	Cytochrome c	*H. sapiens*	105
P00004	Cytochrome c	*E. caballus*	105
P01308	Insulin	*H. sapiens*	110
P01825	Immunoglobulin heavy chain	*H. sapiens*	116
P31417	Fatty acid-binding protein 2	*M. sexta*	132
P61626	Lysozyme C	*H. sapiens*	148
P42212	Green fluorescent protein	*A. victoria*	238
P04637	Tumor suppressor p53	*H. sapiens*	393
P0DTC9	SARS-CoV-2 Nucleoprotein	*SARS-CoV-2*	419
P0CG48	Polyubiquitin-C	*H. sapiens*	685
P0DTC2	SARS-CoV-2 Spike protein	*SARS-CoV-2*	1273

We conducted comprehensive computational experiments across two optimization scenarios: accessibility-only optimization (144 experiments across 12 variants × 12 proteins) focusing solely on minimizing mRNA accessibility, and CAI joint optimization incorporating additional codon adaptation constraints. For Accessibility-CAI joint optimization, we set the CAI target threshold to 0.8.

#### 3.1.1 Evaluation budget

For fair comparison, all methods used exactly 1000 DeepRaccess forward-pass evaluations per run, where one *evaluation* corresponds to one forward pass through the DeepRaccess model on a discrete RNA sequence. In ID3, this budget counts the discrete-sequence evaluations performed in the evaluation track, rather than the internal continuous optimization computations used to update the logits. As shown in Fig. 1, ID3 employs a dual-track architecture: the *optimization track* updates continuous parameters via gradient descent, while the *evaluation track* independently discretizes the current parameters into one discrete RNA candidate at each iteration and evaluates that candidate through a full DeepRaccess forward pass. Thus, 1000 ID3 iterations correspond to 1000 candidate evaluations—directly comparable to GA’s 1000 evaluations (20 individuals × 50 generations) and SA’s 1000 sequential evaluations.

For performance validation, we implemented baseline optimization algorithms: Genetic Algorithm (GA) with population size 20 and 50 generations (20×50=1000 evaluations), and Simulated Annealing (SA) with exponential cooling schedule over 1000 iterations. We also evaluated partial optimization variants (GA-10, SA-10) and the exhaustive combinatorial optimum (C10). These represent a natural alternative to consider when full-sequence gradient-based optimization is unavailable: without a differentiable predictor, one straightforward approach is to restrict optimization to the translation initiation region (the first ∼10 codons downstream of the AUG start codon), where local secondary structure most directly influences ribosomal scanning efficiency (Kozak 1999, Terai and Asai 2020). GA-10 and SA-10 apply stochastic search within this window; C10 exhaustively enumerates all synonymous codon combinations in the same window to establish the combinatorial ceiling. All remaining codon positions are assigned to the most frequent codon for each amino acid. All methods were evaluated across the same 12 proteins with 20 independent runs each, totaling 480 baseline experiments.

### 3.2 Performance analysis

#### 3.2.1 Accessibility optimization

**Figure 3:**
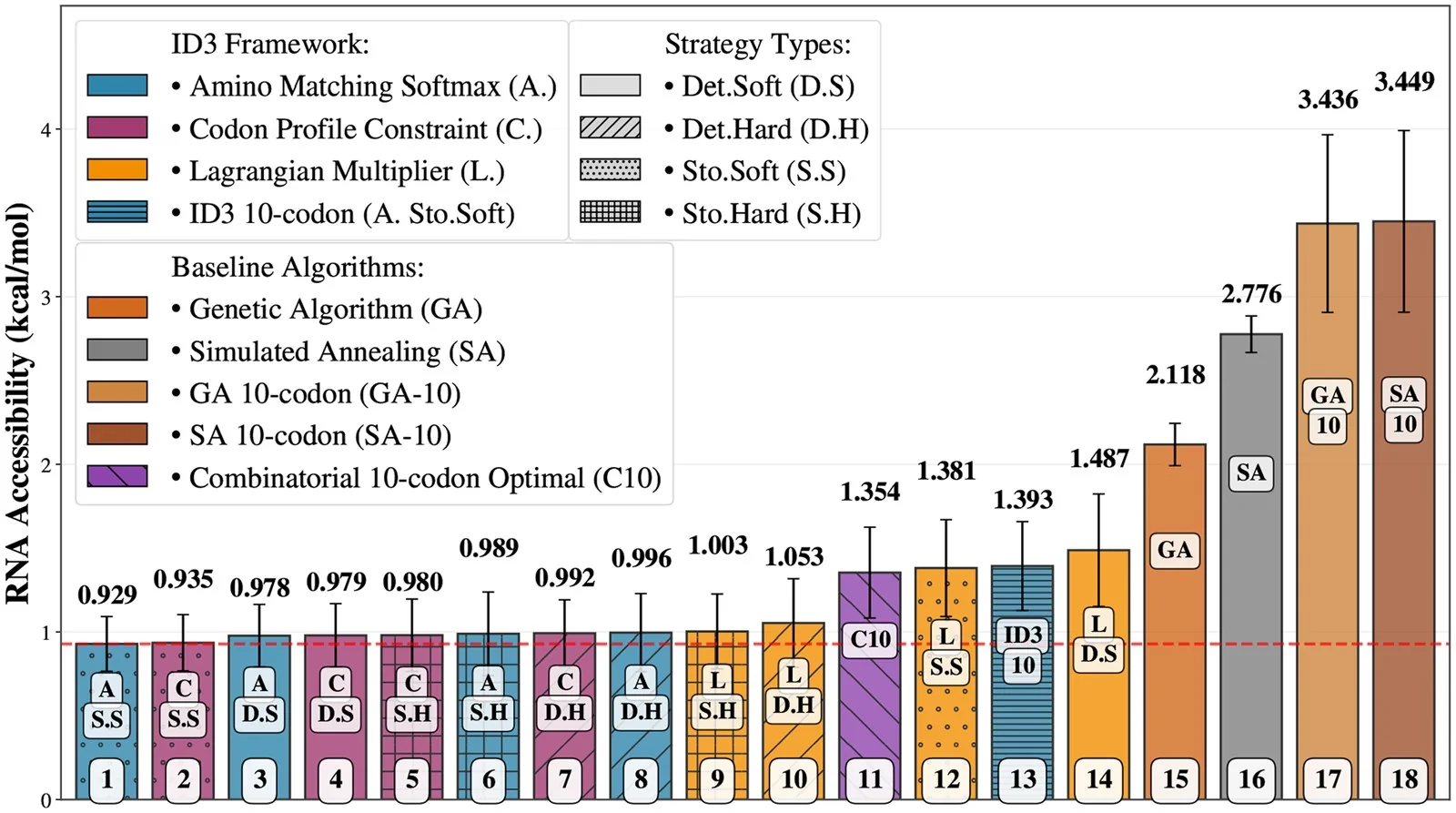
Performance Ranking of ID3 Variants, ID3 10-codon, and Baseline Methods. All 18 methods ranked by mean RNA accessibility (kcal/mol, lower = better). The 12 full-sequence ID3 variants occupy ranks 1–10, 12, and 14, and Amino.Sto.Soft achieves optimal full-sequence performance at 0.929 kcal/mol. The exhaustive combinatorial baseline C10 ranks 11th (1.354 kcal/mol). *ID3 10-codon* (Amino Sto.Soft restricted to the first 10 codons; rank 13, 1.393 kcal/mol) is statistically indistinguishable from C10 in the matched-window comparison. The full-sequence ID3 advantage should therefore be interpreted as a benefit of expanding the optimization scope to the full coding sequence, not as same-window superiority over exhaustive search. Traditional full-sequence methods (GA rank 15; SA rank 16) and 10-codon stochastic baselines (GA-10 rank 17; SA-10 rank 18) follow.

Our computational evaluation establishes Amino.Sto.Soft as the optimal configuration, achieving 0.929 kcal/mol accessibility scores. As shown in Fig. 3, all 12 ID3 variants significantly outperform GA and SA, and 9 of 12 significantly outperform the restricted-window combinatorial optimum C10 after Holm-Bonferroni correction. These comparisons should be interpreted with their optimization scopes in mind: C10 exhaustively searches the first 10 codons, whereas the leading ID3 variants optimize the full coding sequence.

#### 3.2.2 Accessibility-CAI joint optimization

**Figure 4:**
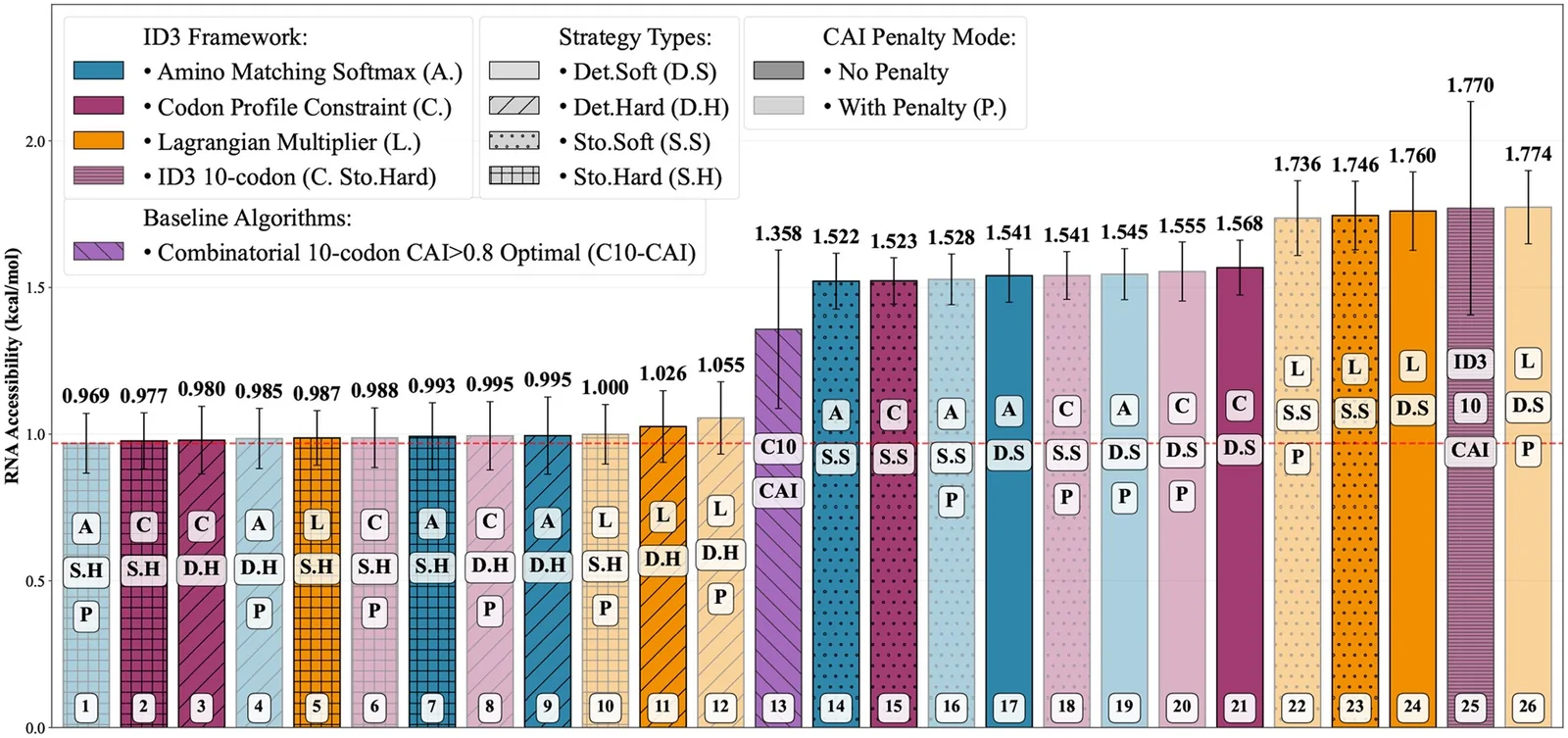
Performance Ranking of ID3 Variants, ID3 10-codon, and Baseline for CAI Joint Optimization. All 26 methods ranked by mean RNA accessibility under CAI≥0.8 constraint. Hard constraint variants lead: Amino.Sto.Hard + Penalty optimal at 0.969 kcal/mol. C10-CAI (exhaustive 10-codon combinatorial with CAI>0.8, rank 13, 1.358 kcal/mol) is the theoretical partial-sequence ceiling. In the matched 10-codon CAI-constrained setting, *ID3 10-codon* (Codon Sto.Hard restricted to the same 10-codon window; rank 25, 1.770 kcal/mol) underperforms C10-CAI, indicating that gradient-based optimization does not replace exhaustive enumeration when the search space is sufficiently small. Full-sequence ID3 performance in the CAI joint task reflects optimization of a much larger context where exhaustive enumeration is infeasible.

**Figure 5:**
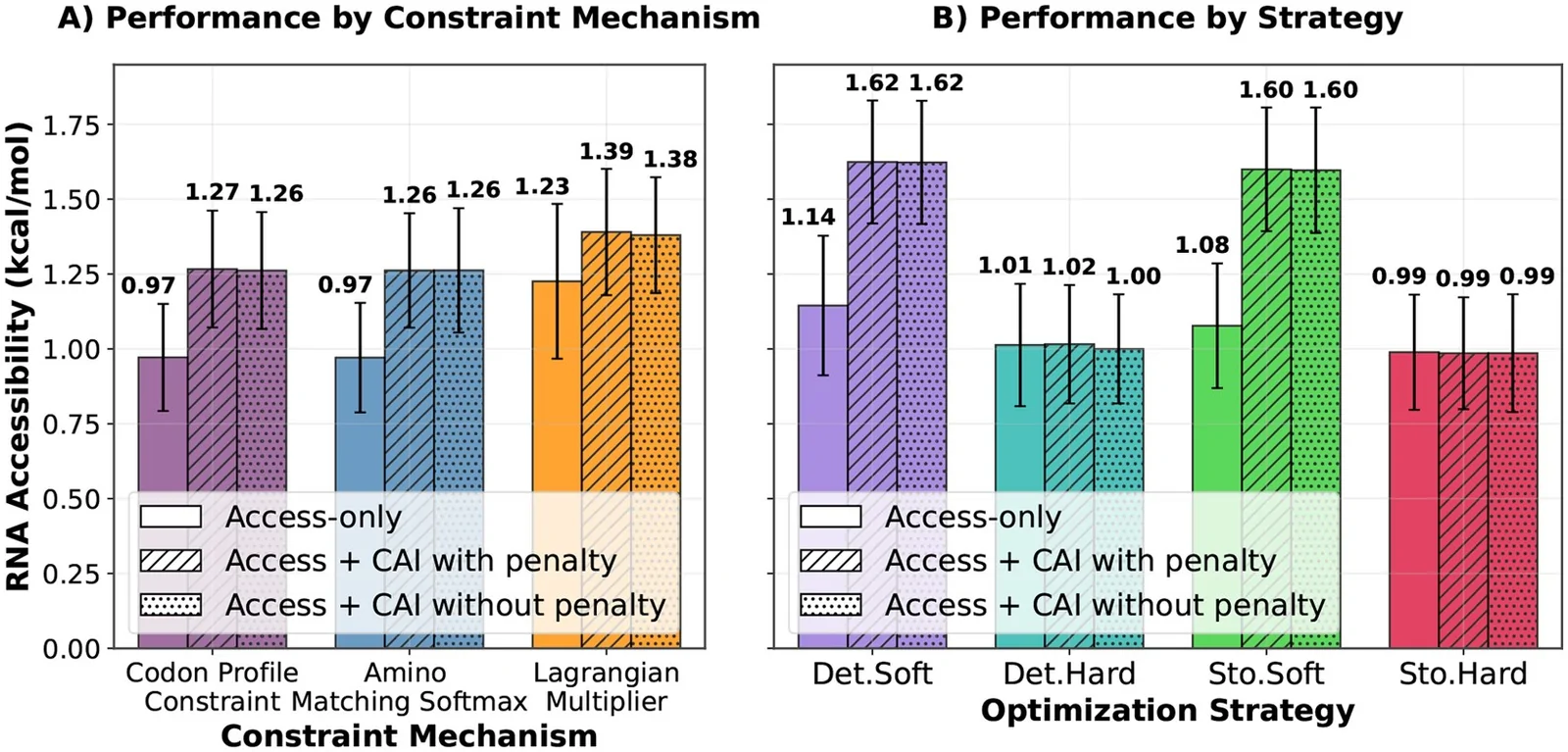
Performance by Constraint and Strategy. Codon Profile and Amino Matching achieve similar performance. Stochastic strategies (Sto.Soft, Sto.Hard) outperform deterministic approaches.

**Figure 6:**
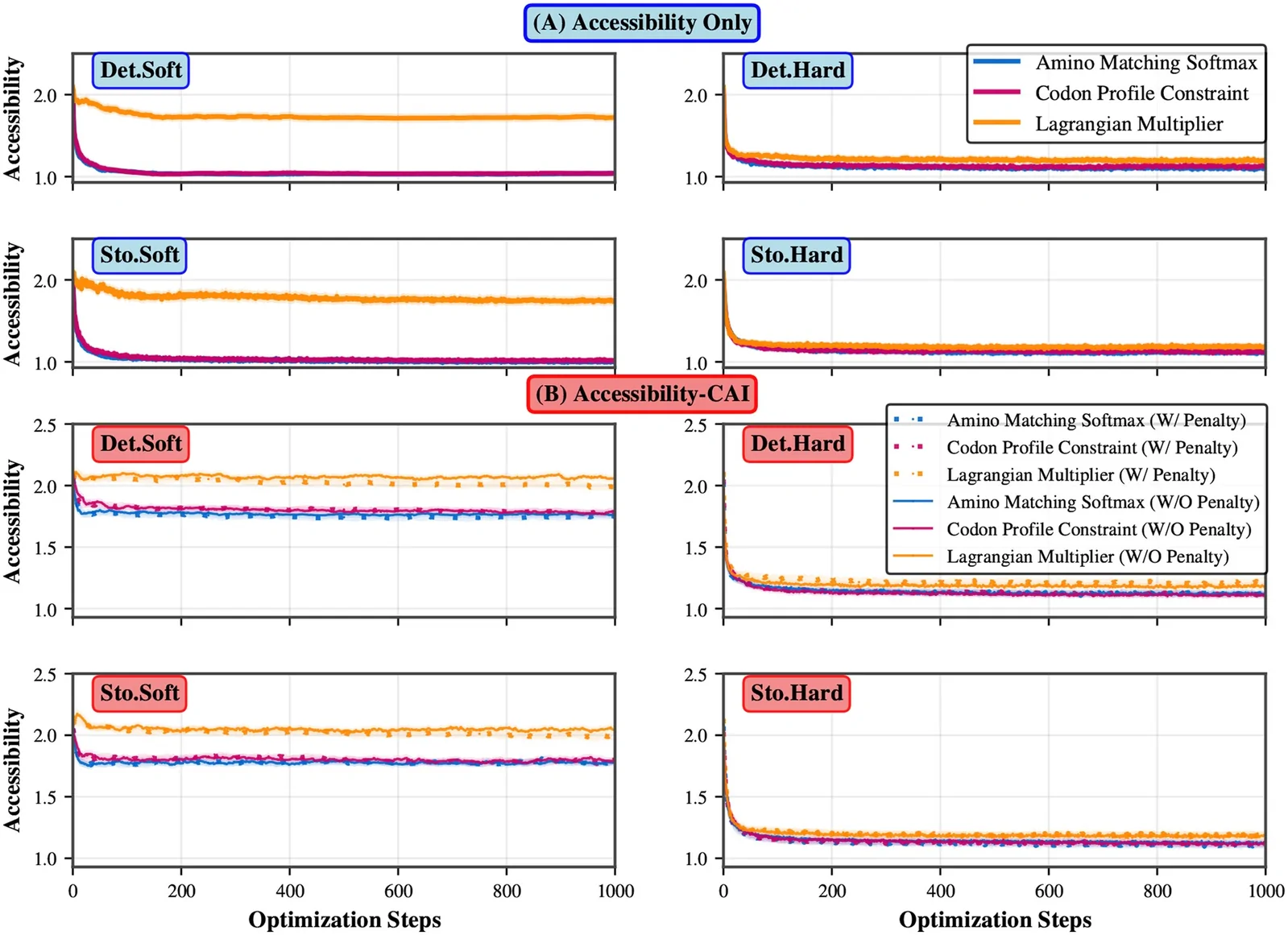
Convergence Analysis by Strategy Type. Comparison across constraint mechanisms and strategy variants The upper panel shows accessibility-only optimization with three convergence trajectories and comparable performance between soft and hard constraints. The lower panel reveals CAI joint optimization where hard constraint variants consistently outperform soft variants.

CAI joint optimization computational experiments reveal accessibility-adaptation trade-offs, with hard constraint variants achieving superior performance in multi-objective scenarios, as shown in Fig. 4. Amino.Sto.Hard with CAI penalty achieves optimal performance (0.969 kcal/mol), while soft constraint methods show degraded performance under codon adaptation requirements. Performance analysis shows no significant difference between Codon Profile and Amino Matching constraint mechanisms (see Supplementary Section S2.2.2, available as supplementary data at *Bioinformatics* online), while stochastic hard strategies consistently outperform soft approaches in CAI joint optimization (Figs 5 and 6).

#### 3.2.3 Constraint mechanism analysis

Figure 6 reveals a fundamental insight into single-objective versus multi-objective optimization dynamics. The accessibility-only optimization (upper panel) demonstrates that soft and hard constraint variants achieve comparable performance, indicating effective exploration within the unified optimization landscape where accessibility serves as the singular objective. However, CAI joint optimization (lower panel) alters this dynamic: the introduction of penalty-based objectives creates target misalignment between accessibility improvement and codon adaptation, causing soft constraint methods to become trapped in suboptimal regions due to conflicting gradient signals. In contrast, hard constraint variants using Straight-Through Estimation maintain optimization efficacy by preserving exact constraint satisfaction without penalty-induced gradient interference, explaining their superior performance in multi-objective scenarios. This is confirmed by computational experiments showing Amino.Sto.Hard with CAI penalty achieving optimal CAI joint optimization performance (0.969 kcal/mol) compared to Amino.Sto.Soft variants ranking significantly lower. This observation establishes that constraint satisfaction mechanisms become important when optimization objectives exhibit spatial or temporal misalignment in the parameter space. Codon Profile and Amino Matching show statistically indistinguishable performance in the present benchmark; this indicates overlap in the tested task, not mathematical equivalence or proven functional complementarity. Codon Profile directly parameterizes synonymous-codon simplexes, whereas Amino Matching parameterizes sequence preferences through nucleotide-level logits followed by an amino-acid compatibility transformation. We retain Amino Matching as a nucleotide-level interface for future objectives, not because this benchmark shows a consistent advantage over Codon Profile. The C10 comparison further clarifies the claim boundary. ID3 does not replace exhaustive search when exhaustive search is feasible: ID3-10 is statistically indistinguishable from C10 in the matched accessibility-only window and underperforms C10-CAI in the matched CAI-constrained window. ID3’s value is enabling full-sequence, constraint-preserving, gradient-based optimization when exhaustive enumeration is infeasible.

Detailed numerical results and statistical comparisons are in Supplementary Section S1 and S2, available as supplementary data at *Bioinformatics* online.

### 3.3 Case study: O15263 protein optimization

**Figure 7:**
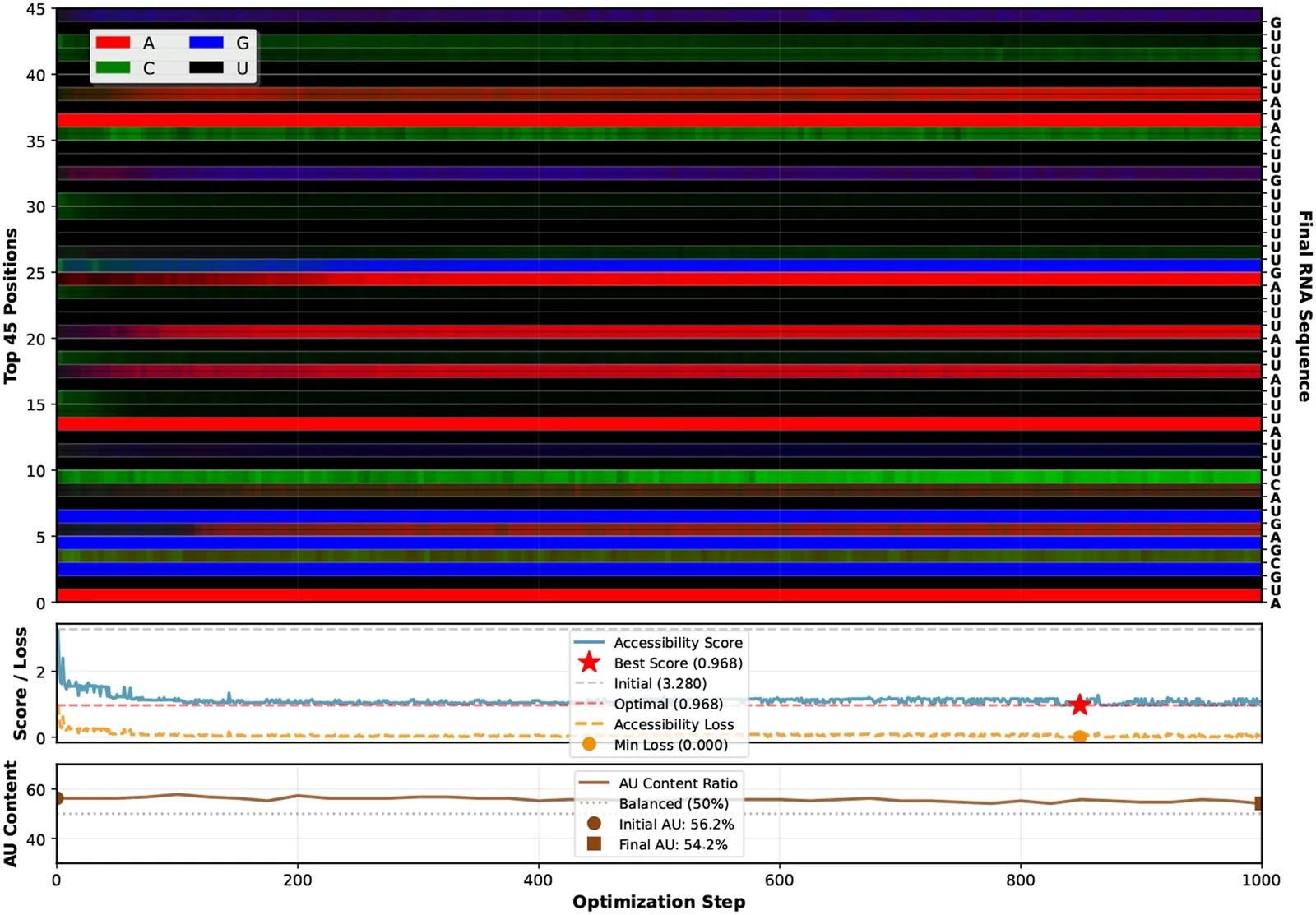
Case Study: O15263 Optimization Trajectory. Dynamic optimization trajectory showing nucleotide probability progression (top), rapid convergence of accessibility from 3.28 to 0.97 kcal/mol (middle). In this illustrative run, higher AU content is associated with weaker base pairing and improved accessibility.

We demonstrate framework effectiveness through O15263 (Defensin beta 4A) optimization using Amino.Sto.Soft. As shown in Fig. 7, the case study reveals dynamic progression of nucleotide probabilities, rapid convergence of sequence probabilities and loss values to stable states, and, in this illustrative run, modest fluctuations in AU content alongside improved accessibility. The observed convergence behavior illustrates the framework’s ability to rapidly improve accessibility in this example, supporting practical applicability for therapeutic mRNA design. As a complementary single-run contrast, Fig. S1, available as supplementary data at *Bioinformatics* online shows an O15263 run under Lagrangian.Det.Soft. The optimizer reaches its best accessibility (1.479 kcal/mol) at step 6 of 1000, then regresses to 1.944 kcal/mol by termination. This early-peak-then-regression pattern is consistent with gradient interference from the soft Lagrangian constraint term and with the broader underperformance of Lagrangian soft variants in Table S1 (see Supplementary Section S2.2.2, available as supplementary data at *Bioinformatics* online).

## 4 Conclusion

We present the ID3 framework for mRNA sequence design, which bridges the discrete–continuous optimization gap through a decoupled optimization–evaluation architecture. ID3 separates continuous probability optimization from discrete sequence evaluation, allowing models to accept probabilistic inputs while preserving the corresponding amino acid sequences of the mRNAs. This design removes the need for model retraining or architectural modification, enabling direct use of existing predictors with probabilistic inputs.

The framework systematically supports 12 constrained variants formed by combining four operational modes (deterministic/stochastic and soft/hard) with three distinct mathematical constraint mechanisms for amino acid preservation, and provides conditional convergence results from the trained model input optimization perspective. Through computational evaluations on mRNA accessibility optimization and Accessibility-CAI joint optimization across diverse protein sequences, ID3 demonstrates superior performance over traditional optimization methods, establishing a principled and practical foundation for gradient-based optimization of discrete biological sequences. Future work could incorporate gradient-conflict mitigation methods (Liu *et al*. 2021, 2025) to improve Pareto efficiency in multi-objective scenarios such as accessibility-CAI joint optimization.

## Supplementary Material

btag667_Supplementary_Data

## Data Availability

The ID3 framework source code, datasets, and all scripts to reproduce the computational experiments are available at https://github.com/Li-Hongmin/ID3.git. Pre-computed results and protein sequence data are archived on Zenodo (DOI: 10.5281/zenodo.18917770).
