# Supplementary material for: Gradient-based optimization for mRNA sequence design
Source: Bioinformatics. 2026 Sep 10;42(9):btag667. doi: 10.1093/bioinformatics/btag667 (PMC13604831; doi:10.1093/bioinformatics/btag667)
Supplement: Supplementary file 1 [file btag667_supplementary_data.pdf]

Bioinformatics, 2025, 1–39
doi:
Advance Access Publication Date: Day Month Year
Computational Biology

Supplementary Material: Gradient-based Optimization for mRNA Sequence Design

Hongmin Li[1,2,*], Goro Terai[1,3], Takumi Otagaki[1,†] and Kiyoshi Asai[1,2,3]

[1]Department of Computational Biology and Medical Sciences, Graduate School of Frontier Sciences, University of Tokyo, 5-1-5 Kashiwanoha, Kashiwa-shi, Chiba 277-8561, Japan
[2]School of Life Science and Technology, Institute of Science Tokyo, 2-12-1 Ookayama, Meguro-ku, Tokyo 152-8550, Japan
[3]AI-Empowered Life Science Initiative (ALIS), Research Organization of Information and Systems (ROIS), 4-1-1 Toranomon, Tokyo 105-6929, Japan
*Corresponding author. lihongmin@edu.k.u-tokyo.ac.jp
†Present address: School of Life Science and Technology, Institute of Science Tokyo, 2-12-1 Ookayama, Meguro-ku, Tokyo 152-8550, Japan.

FOR PUBLISHER ONLY Received on Date Month Year; revised on Date Month Year; accepted on Date Month Year

Abstract

This supplementary material provides comprehensive theoretical analysis, methodological details, and computational validation for the ID3 framework for mRNA sequence design.

Key words: convergence analysis, optimization theory, mRNA design, CAI optimization, constraint mechanisms, statistical validation

S1. Detailed Performance Results

This section provides comprehensive numerical results for all computational evaluations referenced in the main paper.

S1.1. Complete Performance Comparison Table

Table S1 presents the detailed numerical results for all 12 ID3 variants compared with baseline optimization algorithms (GA and SA) across 12 diverse proteins. Each cell shows mean accessibility scores with standard deviations from 20 independent runs.

Table S1. Performance comparison of ID3 variants and baseline algorithms for RNA accessibility optimization (kcal/mol, mean_{std})

Method	O15263	P00004	P01308	P01825	P04637	P0CG48	P0DTC2	P0DTC9	P31417	P42212	P61626	P99999	Avg	Rank
Baseline Methods														
Genetic Algorithm	$1.900_{0.14}$	$1.278_{0.09}$	$2.440_{0.18}$	$1.901_{0.10}$	$2.454_{0.14}$	$2.314_{0.08}$	$2.680_{0.10}$	$2.360_{0.12}$	$2.172_{0.12}$	$2.034_{0.11}$	$2.620_{0.14}$	$1.266_{0.10}$	2.118	14
Simulated Annealing	$2.570_{0.17}$	$1.910_{0.10}$	$3.289_{0.11}$	$2.636_{0.16}$	$3.036_{0.07}$	$2.807_{0.07}$	$3.113_{0.04}$	$2.915_{0.09}$	$3.040_{0.08}$	$2.717_{0.09}$	$3.358_{0.15}$	$1.924_{0.10}$	2.776	15
Genetic Algorithm (10-codon)	$3.563_{0.004}$	$2.789_{0.002}$	$4.480_{0.005}$	$3.184_{0.005}$	$3.481_{0.001}$	$3.124_{0.001}$	$3.582_{0.000}$	$3.548_{0.000}$	$3.970_{0.003}$	$3.203_{0.003}$	$4.565_{0.005}$	$2.887_{0.003}$	3.531	16
Simulated Annealing (10-codon)	$3.583_{0.009}$	$2.791_{0.003}$	$4.515_{0.01}$	$3.194_{0.006}$	$3.486_{0.003}$	$3.125_{0.001}$	$3.583_{0.000}$	$3.549_{0.001}$	$3.982_{0.005}$	$3.210_{0.005}$	$4.587_{0.009}$	$2.895_{0.005}$	3.542	17
ID3 10-codon (Amino Sto.Soft)	$1.341_{0.01}$	$0.985_{0.00}$	$1.822_{0.00}$	$1.561_{0.01}$	$1.691_{0.04}$	$1.082_{0.01}$	$1.654_{0.00}$	$1.679_{0.02}$	$1.148_{0.03}$	$1.272_{0.00}$	$1.294_{0.01}$	$1.189_{0.02}$	1.393	13
Combinatorial 10-codon Optimal	1.256	0.987	1.822	1.521	1.657	1.079	1.645	1.538	1.093	1.270	1.218	1.159	1.354	11
ID3 Framework Variants														
Codon.Sto.Soft	$1.030_{0.09}$	**$0.778_{0.01}$**	$1.227_{0.02}$	$1.167_{0.04}$	$1.012_{0.04}$	$0.748_{0.01}$	$0.912_{0.02}$	$0.751_{0.05}$	$1.060_{0.03}$	$0.882_{0.03}$	$0.917_{0.07}$	$0.731_{0.003}$	0.935	2
Codon.Sto.Hard	$1.299_{0.39}$	$0.905_{0.06}$	$1.222_{0.08}$	$1.193_{0.07}$	$0.992_{0.04}$	$0.806_{0.07}$	$1.038_{0.17}$	$0.814_{0.09}$	$0.966_{0.07}$	**$0.815_{0.05}$**	$0.833_{0.07}$	$0.858_{0.10}$	0.978	4
Codon.Det.Soft	$1.117_{0.09}$	$0.793_{0.02}$	$1.254_{0.02}$	$1.295_{0.05}$	$1.043_{0.02}$	$0.778_{0.02}$	$0.933_{0.02}$	$0.766_{0.05}$	$1.138_{0.05}$	$0.909_{0.04}$	$1.000_{0.04}$	$0.736_{0.004}$	0.980	5
Codon.Det.Hard	$1.247_{0.30}$	$0.924_{0.06}$	$1.247_{0.09}$	$1.235_{0.09}$	$1.029_{0.06}$	$0.809_{0.04}$	$1.113_{0.15}$	$0.788_{0.06}$	$0.990_{0.09}$	$0.823_{0.08}$	$0.859_{0.09}$	$0.853_{0.08}$	0.993	8
Amino.Sto.Soft	**$0.998_{0.02}$**	$0.778_{0.01}$	$1.245_{0.02}$	**$1.156_{0.05}$**	**$0.989_{0.05}$**	**$0.747_{0.009}$**	**$0.891_{0.02}$**	**$0.740_{0.02}$**	$1.018_{0.06}$	$0.910_{0.03}$	$0.945_{0.06}$	**$0.730_{0.004}$**	0.929	1
Amino.Det.Soft	$1.053_{0.06}$	$0.795_{0.02}$	$1.270_{0.01}$	$1.281_{0.04}$	$1.033_{0.02}$	$0.762_{0.008}$	$0.940_{0.02}$	$0.757_{0.06}$	$1.117_{0.08}$	$0.941_{0.03}$	$1.043_{0.05}$	$0.735_{0.01}$	0.977	3
Amino.Sto.Hard	$1.439_{0.48}$	$0.922_{0.05}$	$1.214_{0.15}$	$1.181_{0.10}$	$1.008_{0.07}$	$0.808_{0.03}$	$1.011_{0.14}$	$0.791_{0.05}$	**$0.960_{0.09}$**	$0.817_{0.04}$	**$0.831_{0.10}$**	$0.861_{0.08}$	0.987	6
Amino.Det.Hard	$1.302_{0.42}$	$0.914_{0.07}$	**$1.188_{0.15}$**	$1.223_{0.08}$	$1.009_{0.09}$	$0.834_{0.04}$	$1.045_{0.15}$	$0.781_{0.06}$	$1.043_{0.18}$	$0.819_{0.06}$	$0.846_{0.07}$	$0.885_{0.08}$	0.991	7
Lagrangian.Sto.Hard	$1.356_{0.26}$	$0.802_{0.01}$	$1.240_{0.14}$	$1.280_{0.10}$	$1.041_{0.06}$	$0.805_{0.04}$	$1.047_{0.13}$	$0.859_{0.08}$	$1.061_{0.10}$	$0.880_{0.08}$	$0.852_{0.09}$	$0.799_{0.04}$	1.002	9
Lagrangian.Det.Hard	$1.650_{0.39}$	$0.868_{0.09}$	$1.319_{0.08}$	$1.261_{0.11}$	$1.042_{0.07}$	$0.793_{0.05}$	$1.002_{0.11}$	$0.930_{0.08}$	$1.167_{0.15}$	$0.881_{0.05}$	$0.887_{0.10}$	$0.866_{0.11}$	1.055	10
Lagrangian.Sto.Soft	$1.513_{0.15}$	$1.010_{0.08}$	$1.964_{0.13}$	$1.635_{0.05}$	$1.380_{0.15}$	$1.031_{0.06}$	$1.472_{0.20}$	$1.258_{0.10}$	$1.474_{0.21}$	$1.342_{0.12}$	$1.264_{0.11}$	$1.078_{0.11}$	1.368	12
Lagrangian.Det.Soft	$1.680_{0.35}$	$1.059_{0.11}$	$2.091_{0.18}$	$1.692_{0.08}$	$1.516_{0.16}$	$1.086_{0.06}$	$1.672_{0.23}$	$1.343_{0.10}$	$1.583_{0.20}$	$1.481_{0.15}$	$1.380_{0.14}$	$1.144_{0.11}$	1.477	13

Note: Bold values indicate lowest (best) score per protein. Rankings based on average performance.

S1.2. Accessibility-CAI Results

This section provides detailed results for Accessibility-CAI computational experiments, including both CAI scores achieved and corresponding accessibility performance. Each variant is evaluated in two configurations: the base CAI-aware discretization mechanism alone (no-penalty) and the same base mechanism with an additional penalty-based joint loss during optimization (+Penalty).

© The Author 2025. Published by Oxford University Press. All rights reserved. For permissions, please e-mail: journals.permissions@oup.com

S1.2.1. Combined Accessibility Performance

Table S2 shows the accessibility performance for all 12 variants under both CAI integration configurations. Each variant displays two rows: the row without the +Penalty suffix shows performance with the base CAI-aware discretization mechanism alone, and the +Penalty row shows the same base mechanism combined with penalty-based joint loss during optimization. Rankings are calculated across all 24 entries (12 variants × 2 modes).

Table S2. Accessibility-CAI joint optimization performance (accessibility, kcal/mol, mean_{std})

Method	O15263	P00004	P01308	P01825	P04637	P0CG48	P0DTC2	P0DTC9	P31417	P42212	P61626	P99999	Avg	Rank
Baseline Methods														
ID3 10-codon+CAI (Codon Sto.Hard)	$1.817_{0.06}$	$1.286_{0.00}$	$2.527_{0.01}$	$1.865_{0.02}$	$2.111_{0.00}$	$1.673_{0.02}$	$2.009_{0.01}$	$1.929_{0.05}$	$1.425_{0.01}$	$1.768_{0.01}$	$1.589_{0.05}$	$1.242_{0.01}$	1.770	25
Combinatorial 10-codon Optimal	1.302	0.987	1.822	1.521	1.657	1.079	1.645	1.538	1.093	1.270	1.218	1.159	1.358	13
ID3 Framework Variants														
Codon.Det.Soft	$1.811_{0.11}$	$1.247_{0.11}$	$1.948_{0.09}$	$1.507_{0.06}$	$1.639_{0.09}$	$1.270_{0.08}$	$1.704_{0.15}$	$1.646_{0.07}$	$1.575_{0.07}$	$1.425_{0.06}$	$1.589_{0.11}$	$1.452_{0.11}$	1.568	21
Codon.Det.Soft +Penalty	$1.778_{0.10}$	$1.257_{0.11}$	$1.909_{0.07}$	$1.504_{0.04}$	$1.567_{0.11}$	$1.267_{0.11}$	$1.698_{0.16}$	$1.618_{0.11}$	$1.601_{0.07}$	$1.364_{0.08}$	$1.629_{0.11}$	$1.464_{0.13}$	1.555	20
Codon.Det.Hard	$1.230_{0.31}$	$0.930_{0.06}$	$1.195_{0.23}$	$1.224_{0.08}$	$1.004_{0.07}$	$0.803_{0.04}$	$0.995_{0.13}$	$0.811_{0.07}$	$1.032_{0.16}$	$0.836_{0.06}$	$0.837_{0.09}$	$0.859_{0.09}$	0.980	3
Codon.Det.Hard +Penalty	$1.339_{0.44}$	$0.937_{0.05}$	$1.255_{0.26}$	$1.230_{0.06}$	$1.015_{0.04}$	$0.804_{0.05}$	$0.987_{0.14}$	$0.798_{0.05}$	$0.997_{0.11}$	$0.818_{0.05}$	$0.862_{0.07}$	$0.893_{0.08}$	0.995	8
Codon.Sto.Soft	$1.752_{0.08}$	$1.186_{0.11}$	$1.874_{0.07}$	$1.508_{0.04}$	$1.578_{0.06}$	$1.143_{0.08}$	$1.777_{0.03}$	$1.645_{0.07}$	$1.569_{0.08}$	$1.310_{0.07}$	$1.566_{0.10}$	$1.366_{0.14}$	1.523	15
Codon.Sto.Soft +Penalty	$1.783_{0.12}$	$1.265_{0.06}$	$1.908_{0.08}$	$1.514_{0.03}$	$1.560_{0.07}$	$1.197_{0.10}$	$1.767_{0.08}$	$1.619_{0.08}$	$1.594_{0.06}$	$1.315_{0.09}$	$1.583_{0.07}$	$1.386_{0.12}$	1.541	18
Codon.Sto.Hard	$1.293_{0.32}$	$0.903_{0.06}$	$1.226_{0.11}$	$1.185_{0.10}$	$1.010_{0.05}$	$0.802_{0.05}$	**$0.976_{0.12}$**	**$0.779_{0.06}$**	$1.002_{0.09}$	$0.853_{0.06}$	$0.820_{0.07}$	$0.879_{0.07}$	0.977	2
Codon.Sto.Hard +Penalty	$1.320_{0.32}$	$0.898_{0.06}$	$1.184_{0.13}$	$1.239_{0.09}$	**$0.987_{0.06}$**	$0.808_{0.05}$	$1.033_{0.15}$	$0.787_{0.06}$	$1.038_{0.11}$	$0.825_{0.05}$	$0.861_{0.08}$	$0.872_{0.07}$	0.988	6
Amino.Det.Soft	$1.816_{0.09}$	$1.242_{0.12}$	$1.886_{0.07}$	$1.546_{0.05}$	$1.550_{0.09}$	$1.278_{0.06}$	$1.771_{0.08}$	$1.626_{0.13}$	$1.522_{0.11}$	$1.349_{0.08}$	$1.613_{0.13}$	$1.289_{0.09}$	1.541	17
Amino.Det.Soft +Penalty	$1.800_{0.10}$	$1.235_{0.11}$	$1.869_{0.07}$	$1.499_{0.05}$	$1.579_{0.09}$	$1.286_{0.04}$	$1.781_{0.11}$	$1.629_{0.08}$	$1.556_{0.10}$	$1.328_{0.07}$	$1.608_{0.09}$	$1.375_{0.11}$	1.545	19
Amino.Det.Hard	$1.387_{0.49}$	$0.892_{0.07}$	**$1.180_{0.14}$**	**$1.172_{0.13}$**	$1.014_{0.08}$	$0.814_{0.04}$	$1.084_{0.15}$	$0.812_{0.07}$	$0.990_{0.14}$	$0.853_{0.08}$	$0.869_{0.11}$	$0.872_{0.08}$	0.995	9
Amino.Det.Hard +Penalty	$1.214_{0.27}$	$0.923_{0.05}$	$1.201_{0.13}$	$1.227_{0.16}$	$1.041_{0.07}$	$0.829_{0.03}$	$1.041_{0.14}$	$0.803_{0.06}$	$0.985_{0.08}$	$0.835_{0.08}$	$0.848_{0.09}$	$0.875_{0.07}$	0.985	4
Amino.Sto.Soft	$1.746_{0.12}$	$1.204_{0.15}$	$1.836_{0.12}$	$1.530_{0.06}$	$1.557_{0.10}$	$1.255_{0.05}$	$1.768_{0.09}$	$1.596_{0.11}$	$1.536_{0.13}$	$1.309_{0.06}$	$1.643_{0.06}$	$1.281_{0.10}$	1.522	14
Amino.Sto.Soft +Penalty	$1.772_{0.08}$	$1.244_{0.10}$	$1.867_{0.09}$	$1.521_{0.04}$	$1.541_{0.07}$	$1.250_{0.07}$	$1.749_{0.08}$	$1.646_{0.11}$	$1.523_{0.12}$	$1.311_{0.04}$	$1.573_{0.11}$	$1.337_{0.11}$	1.528	16
Amino.Sto.Hard	$1.487_{0.49}$	$0.898_{0.07}$	$1.237_{0.11}$	$1.214_{0.14}$	$0.997_{0.05}$	$0.805_{0.04}$	$0.995_{0.09}$	$0.787_{0.07}$	**$0.975_{0.09}$**	**$0.796_{0.04}$**	$0.874_{0.10}$	$0.847_{0.08}$	0.993	7
Amino.Sto.Hard +Penalty	**$1.187_{0.32}$**	$0.911_{0.07}$	$1.208_{0.10}$	$1.187_{0.14}$	$0.997_{0.06}$	**$0.802_{0.06}$**	$1.015_{0.12}$	$0.799_{0.06}$	$0.987_{0.08}$	$0.831_{0.05}$	$0.831_{0.08}$	$0.872_{0.07}$	0.969	1
Lagrangian.Det.Soft	$1.828_{0.11}$	$1.454_{0.12}$	$2.044_{0.15}$	$1.689_{0.05}$	$1.766_{0.10}$	$1.459_{0.09}$	$1.953_{0.19}$	$1.767_{0.14}$	$1.916_{0.19}$	$1.969_{0.21}$	$1.691_{0.10}$	$1.590_{0.15}$	1.760	24
Lagrangian.Det.Soft +Penalty	$1.853_{0.10}$	$1.431_{0.13}$	$2.014_{0.09}$	$1.675_{0.09}$	$1.710_{0.12}$	$1.447_{0.10}$	$2.084_{0.04}$	$1.834_{0.12}$	$1.997_{0.27}$	$1.890_{0.23}$	$1.721_{0.09}$	$1.625_{0.12}$	1.774	26
Lagrangian.Det.Hard	$1.333_{0.30}$	$0.833_{0.08}$	$1.270_{0.19}$	$1.282_{0.10}$	$1.036_{0.08}$	$0.810_{0.05}$	$1.135_{0.14}$	$0.920_{0.10}$	$1.169_{0.21}$	$0.872_{0.07}$	$0.846_{0.06}$	$0.809_{0.08}$	1.026	11
Lagrangian.Det.Hard +Penalty	$1.453_{0.28}$	$0.851_{0.09}$	$1.389_{0.18}$	$1.288_{0.12}$	$1.022_{0.06}$	$0.805_{0.06}$	$1.149_{0.22}$	$0.941_{0.08}$	$1.198_{0.18}$	$0.894_{0.06}$	$0.836_{0.07}$	$0.840_{0.08}$	1.055	12
Lagrangian.Sto.Soft	$1.837_{0.15}$	$1.484_{0.11}$	$1.985_{0.06}$	$1.677_{0.06}$	$1.679_{0.13}$	$1.491_{0.07}$	$1.967_{0.11}$	$1.810_{0.11}$	$1.789_{0.16}$	$1.966_{0.24}$	$1.718_{0.07}$	$1.543_{0.13}$	1.746	23
Lagrangian.Sto.Soft +Penalty	$1.839_{0.13}$	$1.430_{0.12}$	$2.032_{0.13}$	$1.646_{0.08}$	$1.746_{0.12}$	$1.507_{0.06}$	$2.022_{0.10}$	$1.796_{0.12}$	$1.798_{0.23}$	$1.774_{0.22}$	$1.720_{0.08}$	$1.528_{0.15}$	1.736	22
Lagrangian.Sto.Hard	$1.279_{0.25}$	**$0.783_{0.02}$**	$1.230_{0.13}$	$1.285_{0.10}$	$1.023_{0.08}$	$0.806_{0.05}$	$1.063_{0.14}$	$0.859_{0.09}$	$1.077_{0.10}$	$0.865_{0.06}$	**$0.812_{0.07}$**	**$0.768_{0.03}$**	0.987	5
Lagrangian.Sto.Hard +Penalty	$1.384_{0.27}$	$0.800_{0.02}$	$1.213_{0.16}$	$1.294_{0.12}$	$1.006_{0.07}$	$0.804_{0.05}$	$1.095_{0.18}$	$0.834_{0.08}$	$1.076_{0.11}$	$0.883_{0.06}$	$0.827_{0.08}$	$0.781_{0.02}$	1.000	10

Note: Each ID3 variant shows two rows: default (No Penalty) and +Penalty. C10 = Combinatorial 10-codon optimal with CAI¿0.8 constraint. Bold = best per protein. First 20 runs.

S1.2.2. Combined CAI Scores

Table S3 presents the CAI scores achieved by each variant under both integration methods, demonstrating the effectiveness of both penalty-based and discretization-based CAI integration approaches. The table follows the same format as Table S2, with each variant showing two rows for the two integration methods.

Table S3. Accessibility-CAI joint optimization scores (CAI values, mean_{std})

Method	O15263	P00004	P01308	P01825	P04637	P0CG48	P0DTC2	P0DTC9	P31417	P42212	P61626	P99999	Avg
Baseline Methods													
ID3 10-codon+CAI (Codon Sto.Hard)	$0.820_{0.02}$	$0.835_{0.05}$	$0.824_{0.00}$	$0.817_{0.02}$	$0.856_{0.01}$	$0.809_{0.02}$	$0.909_{0.02}$	$0.844_{0.04}$	$0.809_{0.01}$	$0.834_{0.01}$	$0.837_{0.04}$	$0.903_{0.02}$	0.842
Combinatorial 10-codon Optimal	0.803	0.903	0.878	0.892	0.979	0.979	0.990	0.978	0.920	0.945	0.891	0.924	0.923
ID3 Framework Variants													
Codon.Det.Soft	$0.809_{0.008}$	$0.807_{0.01}$	$0.804_{0.003}$	$0.807_{0.010}$	$0.801_{0.001}$	$0.800_{0.000}$	$0.800_{0.000}$	$0.805_{0.006}$	$0.804_{0.003}$	$0.803_{0.002}$	$0.802_{0.003}$	$0.806_{0.005}$	0.804
Codon.Det.Soft +Penalty	$0.809_{0.009}$	$0.806_{0.005}$	$0.804_{0.003}$	$0.802_{0.002}$	$0.801_{0.002}$	$0.800_{0.000}$	$0.800_{0.000}$	$0.802_{0.002}$	$0.804_{0.004}$	$0.804_{0.006}$	$0.806_{0.007}$	$0.806_{0.008}$	0.804
Codon.Det.Hard	$0.807_{0.007}$	$0.804_{0.002}$	$0.803_{0.003}$	$0.804_{0.002}$	$0.801_{0.002}$	$0.800_{0.000}$	$0.801_{0.002}$	$0.803_{0.003}$	$0.802_{0.002}$	$0.803_{0.003}$	$0.805_{0.004}$	$0.803_{0.004}$	0.803
Codon.Det.Hard +Penalty	$0.809_{0.009}$	$0.805_{0.003}$	$0.803_{0.003}$	$0.805_{0.004}$	$0.803_{0.004}$	$0.800_{0.000}$	$0.800_{0.000}$	$0.804_{0.008}$	$0.802_{0.002}$	$0.804_{0.005}$	$0.805_{0.004}$	$0.804_{0.003}$	0.804
Codon.Sto.Soft	$0.808_{0.009}$	$0.805_{0.005}$	$0.804_{0.003}$	$0.802_{0.002}$	$0.801_{0.001}$	$0.800_{0.000}$	$0.800_{0.000}$	$0.803_{0.006}$	$0.803_{0.002}$	$0.805_{0.006}$	$0.803_{0.003}$	$0.805_{0.007}$	0.803
Codon.Sto.Soft +Penalty	$0.807_{0.008}$	$0.804_{0.004}$	$0.806_{0.004}$	$0.802_{0.003}$	$0.802_{0.003}$	$0.800_{0.000}$	$0.800_{0.000}$	$0.801_{0.002}$	$0.803_{0.002}$	$0.802_{0.003}$	$0.804_{0.005}$	$0.804_{0.004}$	0.803
Codon.Sto.Hard	$0.806_{0.006}$	$0.805_{0.004}$	$0.802_{0.002}$	$0.802_{0.002}$	$0.801_{0.001}$	$0.800_{0.000}$	$0.800_{0.001}$	$0.803_{0.006}$	$0.802_{0.002}$	$0.801_{0.002}$	$0.803_{0.002}$	$0.803_{0.003}$	0.802
Codon.Sto.Hard +Penalty	$0.807_{0.007}$	$0.803_{0.003}$	$0.805_{0.009}$	$0.803_{0.003}$	$0.801_{0.001}$	$0.800_{0.001}$	$0.800_{0.000}$	$0.801_{0.001}$	$0.801_{0.001}$	$0.801_{0.001}$	$0.803_{0.003}$	$0.803_{0.003}$	0.802
Amino.Det.Soft	$0.812_{0.01}$	$0.809_{0.009}$	$0.805_{0.004}$	$0.809_{0.01}$	$0.801_{0.001}$	$0.801_{0.001}$	$0.800_{0.000}$	$0.808_{0.01}$	$0.806_{0.004}$	$0.803_{0.003}$	$0.805_{0.005}$	$0.807_{0.006}$	0.806
Amino.Det.Soft +Penalty	$0.806_{0.006}$	$0.806_{0.006}$	$0.807_{0.006}$	$0.807_{0.009}$	$0.802_{0.002}$	$0.801_{0.001}$	$0.801_{0.004}$	$0.802_{0.002}$	$0.805_{0.004}$	$0.805_{0.006}$	$0.804_{0.004}$	$0.809_{0.009}$	0.805
Amino.Det.Hard	$0.809_{0.006}$	$0.805_{0.004}$	$0.805_{0.003}$	$0.804_{0.003}$	$0.802_{0.003}$	$0.801_{0.001}$	$0.800_{0.000}$	$0.804_{0.004}$	$0.803_{0.003}$	$0.805_{0.008}$	$0.805_{0.003}$	$0.804_{0.004}$	0.804
Amino.Det.Hard +Penalty	$0.809_{0.006}$	$0.807_{0.005}$	$0.804_{0.002}$	$0.806_{0.005}$	$0.803_{0.002}$	$0.801_{0.001}$	$0.801_{0.000}$	$0.802_{0.001}$	$0.803_{0.002}$	$0.805_{0.008}$	$0.804_{0.002}$	$0.804_{0.003}$	0.804
Amino.Sto.Soft	$0.809_{0.008}$	$0.807_{0.006}$	$0.806_{0.006}$	$0.805_{0.004}$	$0.802_{0.001}$	$0.801_{0.001}$	$0.801_{0.001}$	$0.801_{0.002}$	$0.804_{0.003}$	$0.804_{0.006}$	$0.805_{0.004}$	$0.807_{0.006}$	0.804
Amino.Sto.Soft +Penalty	$0.807_{0.009}$	$0.808_{0.008}$	$0.805_{0.007}$	$0.804_{0.004}$	$0.802_{0.002}$	$0.801_{0.001}$	$0.800_{0.000}$	$0.802_{0.004}$	$0.804_{0.005}$	$0.802_{0.002}$	$0.804_{0.003}$	$0.806_{0.006}$	0.804
Amino.Sto.Hard	$0.807_{0.006}$	$0.804_{0.004}$	$0.805_{0.003}$	$0.805_{0.006}$	$0.802_{0.002}$	$0.801_{0.001}$	$0.801_{0.000}$	$0.804_{0.003}$	$0.803_{0.003}$	$0.804_{0.002}$	$0.804_{0.004}$	$0.804_{0.004}$	0.804
Amino.Sto.Hard +Penalty	$0.806_{0.005}$	$0.805_{0.004}$	$0.803_{0.003}$	$0.806_{0.004}$	$0.802_{0.001}$	$0.800_{0.000}$	$0.801_{0.000}$	$0.804_{0.004}$	$0.803_{0.003}$	$0.804_{0.003}$	$0.804_{0.003}$	$0.803_{0.003}$	0.803
Lagrangian.Det.Soft	$0.812_{0.01}$	$0.810_{0.010}$	$0.808_{0.007}$	$0.804_{0.005}$	$0.803_{0.003}$	$0.801_{0.001}$	$0.802_{0.002}$	$0.806_{0.01}$	$0.803_{0.003}$	$0.805_{0.006}$	$0.826_{0.03}$	$0.808_{0.009}$	0.807
Lagrangian.Det.Soft +Penalty	$0.810_{0.01}$	$0.806_{0.005}$	$0.807_{0.008}$	$0.807_{0.007}$	$0.804_{0.004}$	$0.802_{0.002}$	$0.803_{0.003}$	$0.802_{0.002}$	$0.806_{0.005}$	$0.804_{0.003}$	$0.806_{0.007}$	$0.807_{0.004}$	0.805
Lagrangian.Det.Hard	$0.809_{0.008}$	$0.804_{0.004}$	$0.807_{0.008}$	$0.803_{0.002}$	$0.801_{0.000}$	$0.803_{0.008}$	$0.800_{0.000}$	$0.801_{0.003}$	$0.803_{0.003}$	$0.801_{0.000}$	$0.803_{0.004}$	$0.805_{0.005}$	0.803
Lagrangian.Det.Hard +Penalty	$0.809_{0.007}$	$0.805_{0.004}$	$0.806_{0.006}$	$0.804_{0.004}$	$0.802_{0.002}$	$0.802_{0.005}$	$0.800_{0.000}$	$0.801_{0.002}$	$0.802_{0.002}$	$0.802_{0.002}$	$0.803_{0.004}$	$0.803_{0.003}$	0.803
Lagrangian.Sto.Soft	$0.811_{0.01}$	$0.809_{0.009}$	$0.808_{0.006}$	$0.806_{0.008}$	$0.802_{0.003}$	$0.801_{0.001}$	$0.802_{0.002}$	$0.803_{0.003}$	$0.802_{0.003}$	$0.804_{0.004}$	$0.814_{0.02}$	$0.810_{0.009}$	0.806
Lagrangian.Sto.Soft +Penalty	$0.808_{0.009}$	$0.805_{0.006}$	$0.806_{0.008}$	$0.807_{0.007}$	$0.802_{0.002}$	$0.801_{0.001}$	$0.801_{0.000}$	$0.802_{0.002}$	$0.803_{0.002}$	$0.802_{0.002}$	$0.803_{0.002}$	$0.806_{0.005}$	0.804
Lagrangian.Sto.Hard	$0.806_{0.006}$	$0.804_{0.003}$	$0.805_{0.004}$	$0.803_{0.003}$	$0.801_{0.001}$	$0.800_{0.000}$	$0.800_{0.000}$	$0.801_{0.000}$	$0.803_{0.003}$	$0.801_{0.001}$	$0.802_{0.003}$	$0.804_{0.003}$	0.803
Lagrangian.Sto.Hard +Penalty	$0.807_{0.006}$	$0.804_{0.003}$	$0.804_{0.003}$	$0.803_{0.002}$	$0.801_{0.000}$	$0.800_{0.000}$	$0.800_{0.000}$	$0.801_{0.001}$	$0.801_{0.001}$	$0.801_{0.001}$	$0.802_{0.002}$	$0.803_{0.003}$	0.802

Note: Each ID3 variant shows two rows: default (No Penalty) and +Penalty. C10 = Combinatorial 10-codon optimal (CAI scores at CAI¿0.8 constrained sequences). First 20 runs.

S2. Computational Experiment Setup and Results Analysis

S2.1. Supplementary Computational Experiment Setup

This section details the configurations and implementations for all baseline algorithms evaluated in this study. All computational experiments use normalized datasets with exactly 20 independent runs per configuration to ensure statistical reliability.

S2.1.1. Experimental Scope

The experimental design encompasses 10,080 optimization runs across ID3 framework variants, two ID3 10-codon control experiments, and baseline algorithms, plus 1,364,736 C10 combinatorial evaluations. Table S4 summarizes the complete experimental scope, including one accessibility-only ID3 10-codon control experiment and one accessibility-CAI ID3 10-codon control experiment, both based on the best-performing full-sequence ID3 configurations, as well as three categories of baseline methods: full-sequence optimization (GA, SA), partial-sequence optimization (GA-10, SA-10), and exhaustive combinatorial search (C10).

Table S4. Experimental Design and Scope

Category	Configuration	Runs
**ID3 Framework**		
Accessibility	12 proteins × 3 constraints × 4 variants × 20 runs	2,880
Accessibility-CAI (Penalty)	12 proteins × 3 constraints × 4 variants × 20 runs	2,880
Accessibility-CAI (No-Penalty)	12 proteins × 3 constraints × 4 variants × 20 runs	2,880
Accessibility (10-codon)	12 proteins × 1 best-variant setting × 20 runs	240
Accessibility-CAI (10-codon)	12 proteins × 1 best-variant setting × 20 runs	240
ID3 Subtotal		*9,120*
**Baseline Algorithm**		
Full-sequence (GA, SA)	12 proteins × 2 methods × 20 runs	480
10-codon (GA-10, SA-10)	12 proteins × 2 methods × 20 runs	480
C10 Combinatorial	Exhaustive search: 1,364,736 combinations (6,144–331,776 per protein)	Theoretical
Baseline Subtotal		*960 + C10*
**Total Runs**		**10,080**

S2.1.2. Baseline Algorithm Implementation

All baseline algorithms preserve the target amino acid sequence through synonymous codon substitutions only. The objective function minimizes DeepRaccess accessibility scores for the ATG−19:+15 region across 12 proteins.

Full-Sequence Optimization (GA, SA)

These methods optimize all codon positions throughout the entire sequence. Both algorithms were configured to use exactly 1000 DeepRaccess evaluations per run for fair comparison.

Genetic Algorithm. The GA employs a population of 20 individuals evolving over 49 generations (yielding $20 \times 50 = 1000$ total evaluations). Tournament selection (size 3) identifies parents for reproduction. Offspring are generated through uniform crossover at the codon level (rate 0.8) and synonymous codon mutation (rate 0.1, affecting approximately 10% of optimizable codons). Elitism preserves the top 2 individuals across generations.

Simulated Annealing. The SA performs 999 iterations plus 1 initial evaluation. Temperature decreases exponentially from $T_{\text{init}} = 5.0$ to $T_{\text{final}} = 0.01$ with cooling rate $\alpha = (T_{\text{final}}/T_{\text{init}})^{1/999} \approx 0.9938$. Each iteration randomly replaces 2 codons with synonymous alternatives. Metropolis acceptance criterion $P(\text{accept}) = \exp(-\Delta E/T)$ governs uphill moves. To escape local minima, the algorithm restarts with elevated temperature ($2 \times T_{\text{init}}$) after 100 iterations without improvement (maximum 3 restarts).

Partial-Sequence Optimization (GA-10, SA-10)

These constrained variants optimize only the first 10 codons following the start codon (positions 1-10), while fixing all remaining codons to the highest-probability synonymous codon from E.coli BL21(DE3) codon usage table. This design tests whether localized optimization can achieve comparable performance to full-sequence methods.

The GA-10 and SA-10 variants use identical algorithmic parameters as their full-sequence counterparts (population size 20, 49 generations for GA-10; 999 iterations for SA-10), but restrict all operations (crossover, mutation, neighbor generation) to the 10-codon optimization window. Each method was evaluated with 20 independent runs per protein (240 total runs per method).

Combinatorial Exhaustive Search (C10)

The C10 baseline represents the theoretical optimum achievable through exhaustive combinatorial search over a limited codon window. This method enumerates all possible synonymous codon combinations for the first 10 codons following the start codon, fixing remaining positions to default codons.

The search space comprises 1,364,736 total combinations across 12 proteins, ranging from 6,144 to 331,776 combinations per protein depending on synonymous degeneracy. For each protein, all combinations are evaluated to identify the global optimum within this constrained 10-codon subspace. This baseline establishes an upper bound on partial-sequence optimization performance and enables direct comparison between gradient-based full-sequence methods (ID3) and exhaustive local search.

S2.2. Results Analysis

S2.2.1. Variant Performance Rankings

We evaluated 12 ID3 variants in accessibility and 24 variants in Accessibility-CAI. Complete rankings are provided in Tables S1 and S2. The following analysis focuses on statistically validated performance tiers and identifies mode-specific patterns.

Multiple Comparison Correction

All ranked method-to-method pairwise comparisons in this subsection use Holm-Bonferroni sequential correction [Holm, 1979] to control the family-wise error rate (FWER) at $\alpha = 0.05$. Raw p-values from paired t-tests are ordered from smallest to largest; the k-th smallest p-value is compared against $\alpha/(m-k+1)$, where m is the number of comparisons. For transparency, we report both the unadjusted paired t-test p-values and the Holm-Bonferroni-adjusted p-values for each comparison family. The adjusted values are obtained by applying the Holm-Bonferroni correction to the raw p-values from the paired t-tests. Because many pairwise tests are performed within the same comparison family, a raw p-value can appear significant before correction but become non-significant after Holm-Bonferroni adjustment. We therefore interpret ranked-method statistical significance only from the adjusted p-values reported in the adjusted pairwise matrices; the raw matrices are provided for transparency only. Aggregate analyses of constraint mechanisms, base modes, and CAI integration strategies are reported separately with their own test statistics.

Pairwise Test Definition

For each method pair (A, B), we first average the 20 independent runs within each protein, yielding one protein-level mean accessibility value per method. Let $\bar{x}_{A,i}$ and $\bar{x}_{B,i}$ denote these means for protein $i \in \{1, \ldots, 12\}$, and define the paired difference

$$d_i = \bar{x}_{A,i} - \bar{x}_{B,i}.$$

We then perform a two-sided paired t-test across the 12 proteins:

$$\bar{d} = \frac{1}{12}\sum_{i=1}^{12} d_i, \qquad s_d = \sqrt{\frac{1}{11}\sum_{i=1}^{12}(d_i - \bar{d})^2}, \qquad t = \frac{\bar{d}}{s_d/\sqrt{12}}.$$

Pairing by protein controls for protein-specific difficulty and tests whether one method is consistently better across the shared protein set, rather than comparing absolute score levels without accounting for target-specific variation. The resulting raw p-values are then corrected within each comparison family using the Holm-Bonferroni sequential procedure. If a comparison family contains n methods, then the number of pairwise tests is

$$m = \binom{n}{2} = \frac{n(n-1)}{2}.$$

For the accessibility-only family, $n = 16$ methods (12 ID3 variants + GA + SA + C10 + ID3 10-codon), so

$$m = \binom{16}{2} = \frac{16 \times 15}{2} = 120.$$

For the Accessibility-CAI family, $n = 26$ methods (12 no-penalty ID3 variants + 12 penalty ID3 variants + C10 + ID3 10-codon+CAI), so

$$m = \binom{26}{2} = \frac{26 \times 25}{2} = 325.$$

Let the ordered raw p-values be $p_{(1)} \leq \cdots \leq p_{(m)}$. The Holm-Bonferroni decision rule compares the k-th smallest p-value against

$$p_{(k)} \leq \frac{\alpha}{m-k+1}, \qquad k = 1, \ldots, m.$$

Equivalently, the adjusted values reported in the matrices are the Holm-Bonferroni corrected p-values.

Accessibility-Only Optimization

Tables S5 and S6 present the complete pairwise matrices for all 16 methods (12 ID3 variants + GA + SA + C10 + ID3 10-codon). Table S5 reports the corresponding unadjusted paired t-test p-values for transparency; Table S6 reports Holm-Bonferroni-adjusted p-values and serves as the inferential basis for all ranked-method claims below. Diagonal self-comparisons are omitted. Complete performance rankings appear in Table S1.

Table S5. Complete Pairwise Unadjusted Paired t-Test p-Values (Accessibility-Only Optimization, 16 methods)

	A.Sto.Soft	C.Sto.Soft	A.Det.Soft	C.Sto.Hard	C.Det.Soft	A.Sto.Hard	A.Det.Hard	C.Det.Hard	Lag.Sto.Hrd	Lag.Det.Hrd	C10	Lag.Sto.Sft	ID3-10c	Lag.Det.Sft	GA	SA
**A.Sto.Soft**		0.395	0.001	0.171	0.004	0.200	0.092	0.064	0.047	0.033	<0.001	<0.001	<0.001	<0.001	<0.001	<0.001
**C.Sto.Soft**	0.395		0.004	0.186	0.002	0.215	0.088	0.062	0.038	0.029	<0.001	<0.001	<0.001	<0.001	<0.001	<0.001
**A.Det.Soft**	0.001	0.004		0.978	0.705	0.840	0.724	0.662	0.488	0.170	<0.001	<0.001	<0.001	<0.001	<0.001	<0.001
**C.Sto.Hard**	0.171	0.186	0.978		0.955	0.509	0.172	0.146	0.183	0.033	<0.001	<0.001	<0.001	<0.001	<0.001	<0.001
**C.Det.Soft**	0.004	0.002	0.705	0.955		0.877	0.753	0.694	0.463	0.138	<0.001	<0.001	<0.001	<0.001	<0.001	<0.001
**A.Sto.Hard**	0.200	0.215	0.840	0.509	0.877		0.808	0.762	0.474	0.017	<0.001	<0.001	<0.001	<0.001	<0.001	<0.001
**A.Det.Hard**	0.092	0.088	0.724	0.172	0.753	0.808		0.831	0.529	0.072	<0.001	<0.001	<0.001	<0.001	<0.001	<0.001
**C.Det.Hard**	0.064	0.062	0.662	0.146	0.694	0.762	0.831		0.655	0.132	<0.001	<0.001	<0.001	<0.001	<0.001	<0.001
**Lag.Sto.Hrd**	0.047	0.038	0.488	0.183	0.463	0.474	0.529	0.655		0.062	<0.001	<0.001	<0.001	<0.001	<0.001	<0.001
**Lag.Det.Hrd**	0.033	0.029	0.170	0.033	0.138	0.017	0.072	0.132	0.062		0.006	<0.001	0.003	<0.001	<0.001	<0.001
**C10**	<0.001	<0.001	<0.001	<0.001	<0.001	<0.001	<0.001	<0.001	<0.001	0.006		0.804	0.010	0.064	<0.001	<0.001
**Lag.Sto.Sft**	<0.001	<0.001	<0.001	<0.001	<0.001	<0.001	<0.001	<0.001	<0.001	<0.001	0.804		0.691	<0.001	<0.001	<0.001
**ID3-10c**	<0.001	<0.001	<0.001	<0.001	<0.001	<0.001	<0.001	<0.001	<0.001	0.003	0.010	0.691		0.204	<0.001	<0.001
**Lag.Det.Sft**	<0.001	<0.001	<0.001	<0.001	<0.001	<0.001	<0.001	<0.001	<0.001	<0.001	0.064	<0.001	0.204		<0.001	<0.001
**GA**	<0.001	<0.001	<0.001	<0.001	<0.001	<0.001	<0.001	<0.001	<0.001	<0.001	<0.001	<0.001	<0.001	<0.001		<0.001
**SA**	<0.001	<0.001	<0.001	<0.001	<0.001	<0.001	<0.001	<0.001	<0.001	<0.001	<0.001	<0.001	<0.001	<0.001	<0.001	

Diagonal: self-comparisons omitted. Upper triangle: unadjusted paired t-test p-value. Lower triangle: gray symmetric duplicate. Reported for transparency only; no multiple-comparison correction.

Table S6. Complete Pairwise Holm-Bonferroni Adjusted p-Values (Accessibility-Only Optimization, 16 methods)

	A.Sto.Soft	C.Sto.Soft	A.Det.Soft	C.Sto.Hard	C.Det.Soft	A.Sto.Hard	A.Det.Hard	C.Det.Hard	Lag.Sto.Hrd	Lag.Det.Hrd	C10	Lag.Sto.Sft	ID3-10c	Lag.Det.Sft	GA	SA
**A.Sto.Soft**		1.000	0.066	1.000	0.185	1.000	1.000	1.000	1.000	1.000	0.003*	<0.001*	0.002*	<0.001*	<0.001*	<0.001*
**C.Sto.Soft**	1.000		0.185	1.000	0.088	1.000	1.000	1.000	1.000	1.000	0.004*	<0.001*	0.003*	<0.001*	<0.001*	<0.001*
**A.Det.Soft**	0.066	0.185		1.000	1.000	1.000	1.000	1.000	1.000	1.000	0.015*	<0.001*	0.009*	<0.001*	<0.001*	<0.001*
**C.Sto.Hard**	1.000	1.000	1.000		1.000	1.000	1.000	1.000	1.000	1.000	0.015*	<0.001*	0.009*	<0.001*	<0.001*	<0.001*
**C.Det.Soft**	0.185	0.088	1.000	1.000		1.000	1.000	1.000	1.000	1.000	0.017*	<0.001*	0.011*	<0.001*	<0.001*	<0.001*
**A.Sto.Hard**	1.000	1.000	1.000	1.000	1.000		1.000	1.000	1.000	0.783	0.038*	0.003*	0.021*	<0.001*	<0.001*	<0.001*
**A.Det.Hard**	1.000	1.000	1.000	1.000	1.000	1.000		1.000	1.000	1.000	0.029*	0.002*	0.017*	<0.001*	<0.001*	<0.001*
**C.Det.Hard**	1.000	1.000	1.000	1.000	1.000	1.000	1.000		1.000	1.000	0.015*	<0.001*	0.010*	<0.001*	<0.001*	<0.001*
**Lag.Sto.Hrd**	1.000	1.000	1.000	1.000	1.000	1.000	1.000	1.000		1.000	0.021*	<0.001*	0.011*	<0.001*	<0.001*	<0.001*
**Lag.Det.Hrd**	1.000	1.000	1.000	1.000	1.000	0.783	1.000	1.000	1.000		0.280	0.010*	0.127	0.002*	0.002*	<0.001*
**C10**	0.003*	0.004*	0.015*	0.015*	0.017*	0.038*	0.029*	0.015*	0.021*	0.280		1.000	0.442	1.000	0.002*	<0.001*
**Lag.Sto.Sft**	<0.001*	<0.001*	<0.001*	<0.001*	<0.001*	0.003*	0.002*	<0.001*	<0.001*	0.010*	1.000		1.000	<0.001*	0.006*	<0.001*
**ID3-10c**	0.002*	0.003*	0.009*	0.009*	0.011*	0.021*	0.017*	0.010*	0.011*	0.127	0.442	1.000		1.000	0.003*	<0.001*
**Lag.Det.Sft**	<0.001*	<0.001*	<0.001*	<0.001*	<0.001*	<0.001*	<0.001*	<0.001*	<0.001*	0.002*	1.000	<0.001*	1.000		0.016*	<0.001*
**GA**	<0.001*	<0.001*	<0.001*	<0.001*	<0.001*	<0.001*	<0.001*	<0.001*	<0.001*	0.002*	0.002*	0.006*	0.003*	0.016*		<0.001*
**SA**	<0.001*	<0.001*	<0.001*	<0.001*	<0.001*	<0.001*	<0.001*	<0.001*	<0.001*	<0.001*	<0.001*	<0.001*	<0.001*	<0.001*	<0.001*	

Diagonal: self-comparisons omitted. Upper triangle: Holm-Bonferroni adjusted p-value ($^*p \leq 0.05$). Lower triangle: gray symmetric duplicate.

Of 120 total comparisons, 68 are significant at $\alpha = 0.05$ after Holm-Bonferroni correction. The top two variants, Amino.Sto.Soft (rank 1, 0.929 kcal/mol) and Codon.Sto.Soft (rank 2, 0.935 kcal/mol), are statistically indistinguishable (adjusted $p = 1.00$); both employ stochastic soft discretization. More broadly, the top-8 ID3 variants (ranks 1–8, 0.929–0.993 kcal/mol) form a statistically indistinguishable elite cluster, reflecting the robustness of gradient-based optimization across architectural variants. All 12 ID3 variants significantly outperform GA and SA (adjusted $p < 0.001$ in all 24 comparisons). For C10, 9 of the 12 ID3 variants are significantly better; the exceptions are Lagrangian.Det.Hard (rank 10, adjusted $p = 0.262$), Lagrangian.Sto.Soft (rank 11, adjusted $p = 1.00$), and Lagrangian.Det.Soft (rank 12, adjusted $p = 1.00$), consistent with the broader weakness of Lagrangian soft variants discussed below. For ID3 10-codon (rank 13, mean 1.393 kcal/mol): 9 of 12 full-sequence ID3 variants significantly outperform it (adjusted $p \leq 0.021$); the exceptions are Lagrangian.Det.Hard, Lagrangian.Det.Soft, and Lagrangian.Sto.Soft, which already underperform relative to C10. ID3 10-codon significantly outperforms GA (adjusted $p = 0.003$) and SA (adjusted $p < 0.001$). The comparison between ID3 10-codon and C10 is not significant (adjusted $p = 0.442$), indicating that gradient-based search approaches the exhaustive combinatorial optimum within the matched 10-codon window.

Accessibility-CAI Joint Optimization

Tables S7 and S8 present the complete pairwise matrices for all 26 methods in the CAI joint optimization experiment (12 no-penalty ID3 variants + 12 penalty ID3 variants + C10 + ID3 10-codon+CAI). Table S7 reports the corresponding unadjusted paired t-test p-values for transparency; Table S8 reports Holm-Bonferroni-adjusted p-values and serves as the inferential basis for all ranked-method claims below. Complete performance rankings appear in Table S2.

Table S7. Complete Pairwise Unadjusted Paired t-Test p-Values (Accessibility-CAI Joint Optimization, 26 methods)

	A.Sto.Hrd+P	C.Sto.Hard	C.Det.Hard	C.Sto.Hrd+P	Lag.Sto.Hrd	A.Sto.Hard	C.Det.Hrd+P	A.Det.Hard	A.Det.Hrd+P	Lg.St.Hrd+P	Lag.Det.Hrd	Lg.Dt.Hrd+P	C10	A.Sto.Soft	C.Sto.Soft	A.Sto.Sft+P	C.Sto.Sft+P	A.Det.Soft	A.Det.Sft+P	C.Det.Sft+P	C.Det.Soft	Lg.St.Sft+P	Lag.Sto.Sft	Lag.Det.Sft	ID3-10c+CAI	Lg.Dt.Sft+P
**A.Sto.Hrd+P**		0.972	0.726	0.494	0.608	0.441	0.031	0.169	0.005	0.342	0.045	0.012	<0.001	<0.001	<0.001	<0.001	<0.001	<0.001	<0.001	<0.001	<0.001	<0.001	<0.001	<0.001	<0.001	<0.001
**C.Sto.Hard**	0.972		0.800	0.578	0.630	0.419	0.053	0.209	0.062	0.357	0.056	0.012	<0.001	<0.001	<0.001	<0.001	<0.001	<0.001	<0.001	<0.001	<0.001	<0.001	<0.001	<0.001	<0.001	<0.001
**C.Det.Hard**	0.726	0.800		0.776	0.686	0.591	0.235	0.384	0.027	0.419	0.049	0.019	<0.001	<0.001	<0.001	<0.001	<0.001	<0.001	<0.001	<0.001	<0.001	<0.001	<0.001	<0.001	<0.001	<0.001
**C.Sto.Hrd+P**	0.494	0.578	0.776		0.776	0.539	0.129	0.265	0.090	0.379	0.047	0.011	<0.001	<0.001	<0.001	<0.001	<0.001	<0.001	<0.001	<0.001	<0.001	<0.001	<0.001	<0.001	<0.001	<0.001
**Lag.Sto.Hrd**	0.608	0.630	0.686	0.776		0.851	0.793	0.739	0.620	0.322	<0.001	0.002	<0.001	<0.001	<0.001	<0.001	<0.001	<0.001	<0.001	<0.001	<0.001	<0.001	<0.001	<0.001	<0.001	<0.001
**A.Sto.Hard**	0.441	0.419	0.591	0.539	0.851		0.973	0.881	0.863	0.826	0.260	0.030	0.001	<0.001	<0.001	<0.001	<0.001	<0.001	<0.001	<0.001	<0.001	<0.001	<0.001	<0.001	<0.001	<0.001
**C.Det.Hrd+P**	0.031	0.053	0.235	0.129	0.793	0.973		0.882	0.731	0.827	0.182	0.032	<0.001	<0.001	<0.001	<0.001	<0.001	<0.001	<0.001	<0.001	<0.001	<0.001	<0.001	<0.001	<0.001	<0.001
**A.Det.Hard**	0.169	0.209	0.384	0.265	0.739	0.881	0.882		0.904	0.880	0.192	0.033	<0.001	<0.001	<0.001	<0.001	<0.001	<0.001	<0.001	<0.001	<0.001	<0.001	<0.001	<0.001	<0.001	<0.001
**A.Det.Hrd+P**	0.005	0.062	0.027	0.090	0.620	0.863	0.731	0.904		0.956	0.179	0.052	<0.001	<0.001	<0.001	<0.001	<0.001	<0.001	<0.001	<0.001	<0.001	<0.001	<0.001	<0.001	<0.001	<0.001
**Lg.St.Hrd+P**	0.342	0.357	0.419	0.379	0.322	0.826	0.827	0.880	0.956		0.038	0.004	<0.001	<0.001	<0.001	<0.001	<0.001	<0.001	<0.001	<0.001	<0.001	<0.001	<0.001	<0.001	<0.001	<0.001
**Lag.Det.Hrd**	0.045	0.056	0.049	0.047	<0.001	0.260	0.182	0.192	0.179	0.038		0.041	<0.001	<0.001	<0.001	<0.001	<0.001	<0.001	<0.001	<0.001	<0.001	<0.001	<0.001	<0.001	<0.001	<0.001
**Lg.Dt.Hrd+P**	0.012	0.012	0.019	0.011	0.002	0.030	0.032	0.033	0.052	0.004	0.041		0.002	<0.001	<0.001	<0.001	<0.001	<0.001	<0.001	<0.001	<0.001	<0.001	<0.001	<0.001	<0.001	<0.001
**C10**	<0.001	<0.001	<0.001	<0.001	<0.001	0.001	<0.001	<0.001	<0.001	<0.001	<0.001	0.002		0.011	0.009	0.008	0.008	0.006	0.006	0.005	0.002	<0.001	<0.001	<0.001	<0.001	<0.001
**A.Sto.Soft**	<0.001	<0.001	<0.001	<0.001	<0.001	<0.001	<0.001	<0.001	<0.001	<0.001	<0.001	<0.001	0.011		0.941	0.395	0.320	0.042	0.028	0.111	0.044	<0.001	<0.001	<0.001	0.007	<0.001
**C.Sto.Soft**	<0.001	<0.001	<0.001	<0.001	<0.001	<0.001	<0.001	<0.001	<0.001	<0.001	<0.001	<0.001	0.009	0.941		0.510	0.128	0.303	0.120	0.082	0.018	<0.001	<0.001	<0.001	0.008	<0.001
**A.Sto.Sft+P**	<0.001	<0.001	<0.001	<0.001	<0.001	<0.001	<0.001	<0.001	<0.001	<0.001	<0.001	<0.001	0.008	0.395	0.510		0.410	0.222	0.061	0.110	0.024	<0.001	<0.001	<0.001	0.009	<0.001
**C.Sto.Sft+P**	<0.001	<0.001	<0.001	<0.001	<0.001	<0.001	<0.001	<0.001	<0.001	<0.001	<0.001	<0.001	0.008	0.320	0.128	0.410		0.832	0.561	0.171	0.038	<0.001	<0.001	<0.001	0.013	<0.001
**A.Det.Soft**	<0.001	<0.001	<0.001	<0.001	<0.001	<0.001	<0.001	<0.001	<0.001	<0.001	<0.001	<0.001	0.006	0.042	0.303	0.222	0.832		0.729	0.491	0.170	<0.001	<0.001	<0.001	0.009	<0.001
**A.Det.Sft+P**	<0.001	<0.001	<0.001	<0.001	<0.001	<0.001	<0.001	<0.001	<0.001	<0.001	<0.001	<0.001	0.006	0.028	0.120	0.061	0.561	0.729		0.474	0.137	<0.001	<0.001	<0.001	0.013	<0.001
**C.Det.Sft+P**	<0.001	<0.001	<0.001	<0.001	<0.001	<0.001	<0.001	<0.001	<0.001	<0.001	<0.001	<0.001	0.005	0.111	0.082	0.110	0.171	0.491	0.474		0.109	<0.001	<0.001	<0.001	0.020	<0.001
**C.Det.Soft**	<0.001	<0.001	<0.001	<0.001	<0.001	<0.001	<0.001	<0.001	<0.001	<0.001	<0.001	<0.001	0.002	0.044	0.018	0.024	0.038	0.170	0.137	0.109		<0.001	0.001	<0.001	0.019	<0.001
**Lg.St.Sft+P**	<0.001	<0.001	<0.001	<0.001	<0.001	<0.001	<0.001	<0.001	<0.001	<0.001	<0.001	<0.001	<0.001	<0.001	<0.001	<0.001	<0.001	<0.001	<0.001	<0.001	<0.001		0.471	0.204	0.615	0.045
**Lag.Sto.Sft**	<0.001	<0.001	<0.001	<0.001	<0.001	<0.001	<0.001	<0.001	<0.001	<0.001	<0.001	<0.001	<0.001	<0.001	<0.001	<0.001	<0.001	<0.001	<0.001	<0.001	0.001	0.471		0.354	0.768	0.212
**Lag.Det.Sft**	<0.001	<0.001	<0.001	<0.001	<0.001	<0.001	<0.001	<0.001	<0.001	<0.001	<0.001	<0.001	<0.001	<0.001	<0.001	<0.001	<0.001	<0.001	<0.001	<0.001	<0.001	0.204	0.354		0.909	0.408
**ID3-10c+CAI**	<0.001	<0.001	<0.001	<0.001	<0.001	<0.001	<0.001	<0.001	<0.001	<0.001	<0.001	<0.001	<0.001	0.007	0.008	0.009	0.013	0.009	0.013	0.020	0.019	0.615	0.768	0.909		0.956
**Lg.Dt.Sft+P**	<0.001	<0.001	<0.001	<0.001	<0.001	<0.001	<0.001	<0.001	<0.001	<0.001	<0.001	<0.001	<0.001	<0.001	<0.001	<0.001	<0.001	<0.001	<0.001	<0.001	<0.001	0.045	0.212	0.408	0.956	

Diagonal: self-comparisons omitted. Upper triangle: unadjusted paired t-test p-value. Lower triangle: gray symmetric duplicate. Reported for transparency only; no multiple-comparison correction.

Table S8. Complete Pairwise Holm-Bonferroni Adjusted p-Values (Accessibility-CAI Joint Optimization, 26 methods)

	A.Sto.Hrd+P	C.Sto.Hard	C.Det.Hard	C.Sto.Hrd+P	Lag.Sto.Hrd	A.Sto.Hard	C.Det.Hrd+P	A.Det.Hard	A.Det.Hrd+P	Lg.St.Hrd+P	Lag.Det.Hrd	Lg.Dt.Hrd+P	C10	A.Sto.Soft	C.Sto.Soft	A.Sto.Sft+P	C.Sto.Sft+P	A.Det.Soft	A.Det.Sft+P	C.Det.Sft+P	C.Det.Soft	Lg.St.Sft+P	Lag.Sto.Sft	Lag.Det.Sft	ID3-10c+CAI	Lg.Dt.Sft+P
**A.Sto.Hrd+P**		1.000	1.000	1.000	1.000	1.000	1.000	1.000	0.535	1.000	1.000	1.000	0.037*	<0.001*	<0.001*	<0.001*	<0.001*	<0.001*	<0.001*	<0.001*	<0.001*	<0.001*	<0.001*	<0.001*	<0.001*	<0.001*
**C.Sto.Hard**	1.000		1.000	1.000	1.000	1.000	1.000	1.000	1.000	1.000	1.000	1.000	0.048*	<0.001*	<0.001*	<0.001*	<0.001*	<0.001*	<0.001*	<0.001*	<0.001*	<0.001*	<0.001*	<0.001*	<0.001*	<0.001*
**C.Det.Hard**	1.000	1.000		1.000	1.000	1.000	1.000	1.000	1.000	1.000	1.000	1.000	0.044*	<0.001*	<0.001*	<0.001*	<0.001*	<0.001*	<0.001*	<0.001*	<0.001*	<0.001*	<0.001*	<0.001*	<0.001*	<0.001*
**C.Sto.Hrd+P**	1.000	1.000	1.000		1.000	1.000	1.000	1.000	1.000	1.000	1.000	1.000	0.062	<0.001*	<0.001*	<0.001*	<0.001*	<0.001*	<0.001*	<0.001*	<0.001*	<0.001*	<0.001*	<0.001*	<0.001*	<0.001*
**Lag.Sto.Hrd**	1.000	1.000	1.000	1.000		1.000	1.000	1.000	1.000	1.000	0.099	0.290	0.030*	<0.001*	<0.001*	<0.001*	<0.001*	<0.001*	<0.001*	<0.001*	<0.001*	<0.001*	<0.001*	<0.001*	<0.001*	<0.001*
**A.Sto.Hard**	1.000	1.000	1.000	1.000	1.000		1.000	1.000	1.000	1.000	1.000	1.000	0.128	<0.001*	<0.001*	<0.001*	<0.001*	<0.001*	<0.001*	<0.001*	<0.001*	<0.001*	<0.001*	<0.001*	0.001*	<0.001*
**C.Det.Hrd+P**	1.000	1.000	1.000	1.000	1.000	1.000		1.000	1.000	1.000	1.000	1.000	0.069	<0.001*	<0.001*	<0.001*	<0.001*	<0.001*	<0.001*	<0.001*	<0.001*	<0.001*	<0.001*	<0.001*	<0.001*	<0.001*
**A.Det.Hard**	1.000	1.000	1.000	1.000	1.000	1.000	1.000		1.000	1.000	1.000	1.000	0.070	<0.001*	<0.001*	<0.001*	<0.001*	<0.001*	<0.001*	<0.001*	<0.001*	<0.001*	<0.001*	<0.001*	<0.001*	<0.001*
**A.Det.Hrd+P**	0.535	1.000	1.000	1.000	1.000	1.000	1.000	1.000		1.000	1.000	1.000	0.050	<0.001*	<0.001*	<0.001*	<0.001*	<0.001*	<0.001*	<0.001*	<0.001*	<0.001*	<0.001*	<0.001*	<0.001*	<0.001*
**Lg.St.Hrd+P**	1.000	1.000	1.000	1.000	1.000	1.000	1.000	1.000	1.000		1.000	0.518	0.070	<0.001*	<0.001*	<0.001*	<0.001*	<0.001*	<0.001*	<0.001*	<0.001*	<0.001*	<0.001*	<0.001*	<0.001*	<0.001*
**Lag.Det.Hrd**	1.000	1.000	1.000	1.000	0.099	1.000	1.000	1.000	1.000	1.000		1.000	0.077	<0.001*	<0.001*	<0.001*	<0.001*	<0.001*	<0.001*	<0.001*	<0.001*	<0.001*	<0.001*	<0.001*	<0.001*	<0.001*
**Lg.Dt.Hrd+P**	1.000	1.000	1.000	1.000	0.290	1.000	1.000	1.000	1.000	0.518	1.000		0.256	<0.001*	<0.001*	<0.001*	<0.001*	<0.001*	<0.001*	<0.001*	<0.001*	<0.001*	<0.001*	<0.001*	0.001*	<0.001*
**C10**	0.037*	0.048*	0.044*	0.062	0.030*	0.128	0.069	0.070	0.050	0.070	0.077	0.256		1.000	0.952	0.875	0.842	0.684	0.654	0.536	0.243	0.004*	0.009*	0.007*	<0.001*	0.009*
**A.Sto.Soft**	<0.001*	<0.001*	<0.001*	<0.001*	<0.001*	<0.001*	<0.001*	<0.001*	<0.001*	<0.001*	<0.001*	<0.001*	1.000		1.000	1.000	1.000	1.000	1.000	1.000	1.000	0.004*	0.049*	0.044*	0.764	0.016*
**C.Sto.Soft**	<0.001*	<0.001*	<0.001*	<0.001*	<0.001*	<0.001*	<0.001*	<0.001*	<0.001*	<0.001*	<0.001*	<0.001*	0.952	1.000		1.000	1.000	1.000	1.000	1.000	1.000	0.006*	0.062	0.037*	0.891	0.010*
**A.Sto.Sft+P**	<0.001*	<0.001*	<0.001*	<0.001*	<0.001*	<0.001*	<0.001*	<0.001*	<0.001*	<0.001*	<0.001*	<0.001*	0.875	1.000	1.000		1.000	1.000	1.000	1.000	1.000	0.005*	0.058	0.047*	0.918	0.017*
**C.Sto.Sft+P**	<0.001*	<0.001*	<0.001*	<0.001*	<0.001*	<0.001*	<0.001*	<0.001*	<0.001*	<0.001*	<0.001*	<0.001*	0.842	1.000	1.000	1.000		1.000	1.000	1.000	1.000	0.015*	0.097	0.063	1.000	0.025*
**A.Det.Soft**	<0.001*	<0.001*	<0.001*	<0.001*	<0.001*	<0.001*	<0.001*	<0.001*	<0.001*	<0.001*	<0.001*	<0.001*	0.684	1.000	1.000	1.000	1.000		1.000	1.000	1.000	0.009*	0.075	0.074	0.945	0.037*
**A.Det.Sft+P**	<0.001*	<0.001*	<0.001*	<0.001*	<0.001*	<0.001*	<0.001*	<0.001*	<0.001*	<0.001*	<0.001*	<0.001*	0.654	1.000	1.000	1.000	1.000	1.000		1.000	1.000	0.006*	0.083	0.068	1.000	0.024*
**C.Det.Sft+P**	<0.001*	<0.001*	<0.001*	<0.001*	<0.001*	<0.001*	<0.001*	<0.001*	<0.001*	<0.001*	<0.001*	<0.001*	0.536	1.000	1.000	1.000	1.000	1.000	1.000		1.000	0.023*	0.122	0.073	1.000	0.041*
**C.Det.Soft**	<0.001*	<0.001*	<0.001*	<0.001*	<0.001*	<0.001*	<0.001*	<0.001*	<0.001*	<0.001*	<0.001*	<0.001*	0.243	1.000	1.000	1.000	1.000	1.000	1.000	1.000		0.024*	0.134	0.070	1.000	0.048*
**Lg.St.Sft+P**	<0.001*	<0.001*	<0.001*	<0.001*	<0.001*	<0.001*	<0.001*	<0.001*	<0.001*	<0.001*	<0.001*	<0.001*	0.004*	0.004*	0.006*	0.005*	0.015*	0.009*	0.006*	0.023*	0.024*		1.000	1.000	1.000	1.000
**Lag.Sto.Sft**	<0.001*	<0.001*	<0.001*	<0.001*	<0.001*	<0.001*	<0.001*	<0.001*	<0.001*	<0.001*	<0.001*	<0.001*	0.009*	0.049*	0.062	0.058	0.097	0.075	0.083	0.122	0.134	1.000		1.000	1.000	1.000
**Lag.Det.Sft**	<0.001*	<0.001*	<0.001*	<0.001*	<0.001*	<0.001*	<0.001*	<0.001*	<0.001*	<0.001*	<0.001*	<0.001*	0.007*	0.044*	0.037*	0.047*	0.063	0.074	0.068	0.073	0.070	1.000	1.000		1.000	1.000
**ID3-10c+CAI**	<0.001*	<0.001*	<0.001*	<0.001*	<0.001*	0.001*	<0.001*	<0.001*	<0.001*	<0.001*	<0.001*	0.001*	<0.001*	0.764	0.891	0.918	1.000	0.945	1.000	1.000	1.000	1.000	1.000	1.000		1.000
**Lg.Dt.Sft+P**	<0.001*	<0.001*	<0.001*	<0.001*	<0.001*	<0.001*	<0.001*	<0.001*	<0.001*	<0.001*	<0.001*	<0.001*	0.009*	0.016*	0.010*	0.017*	0.025*	0.037*	0.024*	0.041*	0.048*	1.000	1.000	1.000	1.000	

Diagonal: self-comparisons omitted. Upper triangle: Holm-Bonferroni adjusted p-value ($^*p \leq 0.05$). Lower triangle: gray symmetric duplicate.

Of 325 total comparisons, 185 are significant at $\alpha = 0.05$. The top three methods all employ hard discretization: Amino.Sto.Hard+Penalty (rank 1, 0.969 kcal/mol), Codon.Sto.Hard (rank 2, 0.977 kcal/mol), and Codon.Det.Hard (rank 3, 0.980 kcal/mol). These three are mutually statistically indistinguishable (adjusted $p \geq 0.05$), forming an elite performance tier. The added same-window method ID3 10-codon+CAI ranks 25th (1.770 kcal/mol) and is significantly worse than C10-CAI (adjusted $p = 4.03 \times 10^{-4}$), confirming a substantial optimization gap for the joint accessibility-CAI objective within the restricted 10-codon window. Relative to C10, four full-sequence hard-mode ID3 variants significantly outperform it after Holm-Bonferroni correction (Amino.Sto.Hard+Penalty, Codon.Sto.Hard, Codon.Det.Hard, and Lagrangian.Sto.Hard), four Lagrangian soft variants significantly underperform it, and the remaining 16 full-sequence variants are statistically indistinguishable from it.

S2.2.2. Constraint Mechanism Comparisons

The ID3 framework enforces amino acid conservation through three distinct mathematical approaches: *Codon Profile Constraint* directly parameterizes probabilities over valid synonymous codons; *Amino Matching Softmax* retains nucleotide-level parameters but maps them to valid codon probabilities through a restricted softmax; *Lagrangian Multiplier* uses soft penalty-based constraint enforcement in the

full nucleotide space. Despite these mathematical differences, we investigate whether these mechanisms yield distinguishable optimization performance in the present benchmark.

Accessibility

Table S9 presents statistical comparison of the three constraint mechanisms (960 runs each: 48 configurations × 20 runs).

Table S9. Constraint Mechanism Statistical Comparison (Accessibility)

Mechanism	Mean (kcal/mol)	Std	vs. Codon Diff	Cohen's d	p-value
Codon Profile	0.972	0.195	–	–	–
Amino Matching	0.973	0.212	-0.001	-0.005	0.922
Lagrangian	1.231	0.349	-0.259	-0.918	<0.001
Omnibus tests: ANOVA F=314.31 (p<0.001), Kruskal-Wallis H=420.44 (p<0.001)					

In this accessibility benchmark, Codon Profile and Amino Matching are statistically indistinguishable ($p = 0.922$; mean difference 0.001 kcal/mol), whereas Lagrangian is 0.259 kcal/mol worse on average ($p<0.001$, Cohen's d $\approx$ -0.9). We interpret the Codon-versus-Amino result as empirical indistinguishability, with partial benchmark-level redundancy in the present task, rather than proof of universal mathematical equivalence or demonstrated functional complementarity. The similar performance is expected because both mechanisms restrict optimization to valid synonymous-codon choices and evaluate the resulting codon distributions through the same reconstruction and DeepRaccess pipeline. The present experiments do not identify a tested scenario in which Amino Matching consistently outperforms Codon Profile; under the accessibility-only objective, the two mechanisms should therefore be viewed as benchmark-level alternatives with overlapping performance. They remain distinct framework components because they expose different parameter spaces. Codon Profile directly parameterizes the synonymous-codon simplex for each amino-acid position. Amino Matching Softmax instead parameterizes sequence preferences through nucleotide-level logits followed by an amino-acid compatibility transformation. This nucleotide-level interface can expose shared structure across synonymous codons that is absent from direct codon-simplex parameterization. We retain Amino Matching as an extensible nucleotide-level interface for future objectives or regularizers that operate at nucleotide resolution, not because the current benchmark proves a consistent performance advantage over Codon Profile. Thus, the present benchmark supports empirical overlap, not mathematical equivalence or a general redundancy claim.

To complement the successful O15263 example in the main manuscript, Figure S1 shows one accessibility-only Lagrangian.Det.Soft run on the same protein (1000 iterations). The run reaches its best discrete accessibility of **1.479 kcal/mol at iteration 6**, then regresses to 1.944 kcal/mol by termination.

This figure is included as an illustrative single-run contrast rather than as a summary over runs. The directly observable pattern is that a good discrete candidate appears early, but subsequent updates do not preserve it. We therefore present it as qualitative evidence consistent with gradient interference from the soft Lagrangian penalty term, rather than as standalone proof of the mechanism. This interpretation is also consistent with the broader underperformance of Lagrangian soft variants in Table S1. Panel (c) reports AU content only as a descriptive sequence-composition trace; AU content is not an explicit optimization objective in this experiment.

Accessibility-CAI

Table S10 presents statistical comparison combining both CAI integration methods ($n = 1{,}920$ per constraint).

Table S10. Constraint Mechanism Statistical Comparison (Accessibility-CAI)

Mechanism	Mean (kcal/mol)	Std	vs. Codon Diff	Cohen's d	p-value
Codon Profile	1.266	0.357	–	–	–
Amino Matching	1.260	0.354	-0.006	-0.017	0.602
Lagrangian	1.386	0.437	-0.120	-0.30	<0.001
Combining 960 penalty-based + 960 no-penalty runs per mechanism					

Codon Profile and Amino Matching remain statistically indistinguishable in the CAI benchmark ($p = 0.602$). Lagrangian's gap shrinks from 26% (accessibility) to 9–10% (CAI mode) but remains significant ($p<0.001$).

S2.2.3. Base Mode Comparisons

Accessibility

Table S11 compares the four base operational modes (720 runs each: 60 configurations × 20 runs).

Stochastic hard mode achieves best performance (0.991 kcal/mol), with significant differences among all four modes (ANOVA F=45.88, $p<0.001$).

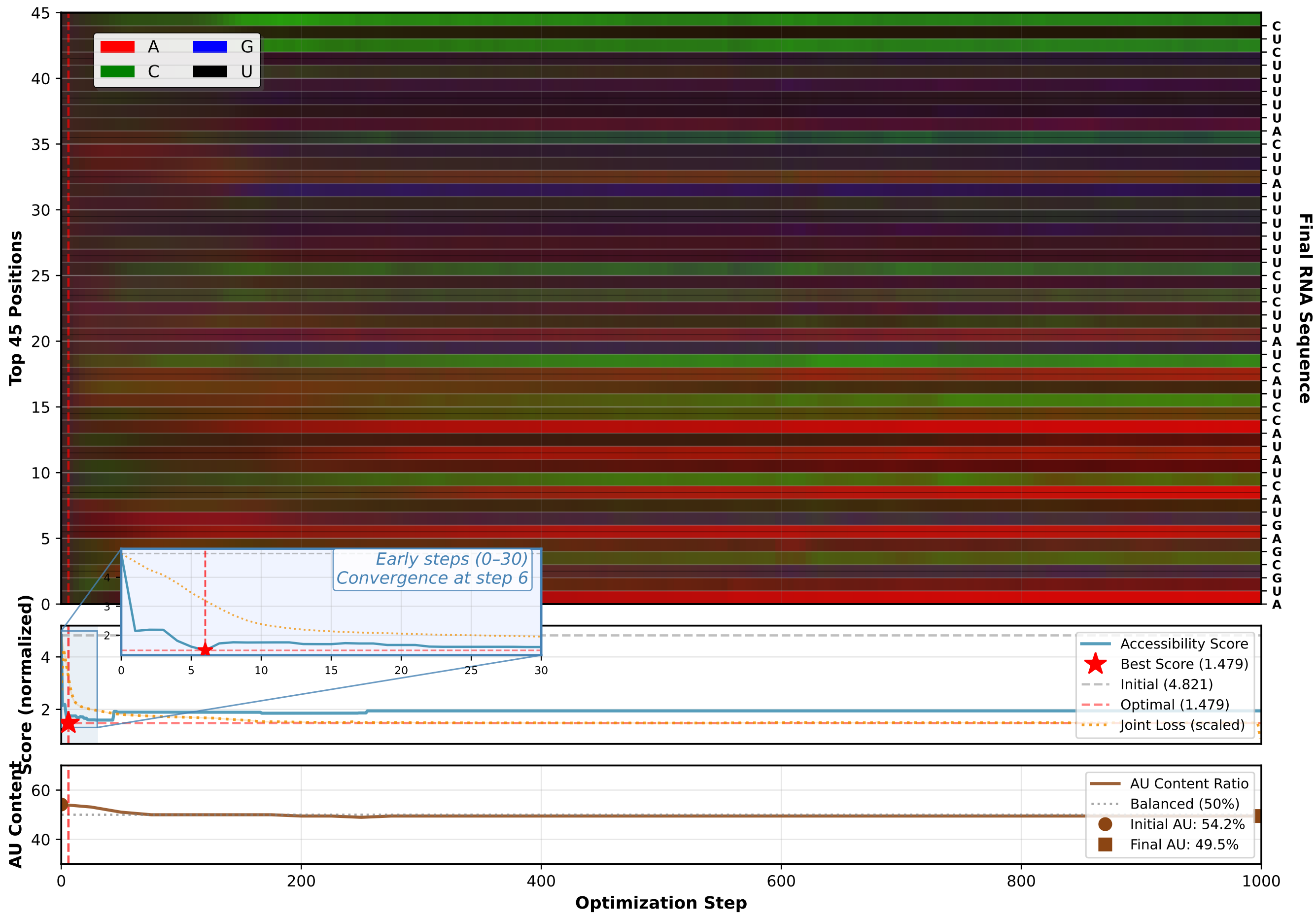

Fig. S1: Illustrative single-run contrast: Lagrangian.Det.Soft optimization of O15263 (1000 iterations). This figure complements the successful O15263 example in the main manuscript and is not intended as an average trajectory over runs. **(a)** Nucleotide probability evolution across iterations. **(b)** Accessibility score and total loss trajectory: best discrete accessibility of 1.479 kcal/mol is achieved at step 6, then worsens to 1.944 kcal/mol by termination. **(c)** AU-content trajectory shown as a descriptive sequence-composition summary; AU content is not an optimization objective.

Table S11. Base Mode Statistical Comparison (Accessibility)

Base Mode	Mean (kcal/mol)	Std	Rank
Sto.Hard	0.991	0.230	1 (best)
Det.Hard	1.014	0.238	2
Sto.Soft	1.082	0.301	3
Det.Soft	1.148	0.344	4 (worst)
ANOVA: F=45.88, p<0.001 (significant differences among modes)			

Accessibility-CAI

Table S12 compares the four base modes in CAI optimization (combining penalty + no-penalty runs).

Hard modes significantly outperform soft modes. Stochastic vs. deterministic difference is marginal and not significant.

S2.2.4. Baseline Algorithm Comparisons

ID3 vs. Genetic Algorithm and Simulated Annealing

Genetic Algorithm (GA) and Simulated Annealing (SA) are widely-used stochastic optimization methods for sequence design. Both operate by iteratively proposing and evaluating sequence mutations without gradient information. In the accessibility-only ranking (Table S1), all 12 full-sequence ID3 variants outperform both GA and SA after Holm-Bonferroni correction in the complete pairwise

Table S12. Base Mode Statistical Comparison (Accessibility-CAI)

Base Mode	Mean (kcal/mol)	Std	Rank
Sto.Hard	0.986	0.011	1 (best)
Det.Hard	1.006	0.029	2
Sto.Soft	1.599	0.110	3
Det.Soft	1.624	0.111	4 (worst)

Combining penalty + no-penalty runs (6 variants per mode)

matrix (Table S6). Accessibility-CAI results are reported separately in Table S2; GA and SA are not treated as matched CAI-joint baselines in this study.

ID3 vs. C10 Combinatorial Optimal

The C10 baseline represents the theoretical optimum found by exhaustive combinatorial search over the first 10 codon positions (1,364,736 total combinations across 12 proteins, with search space ranging from 6,144 to 331,776 combinations per protein). Remaining positions were fixed to default codons.

95.6% of combinations satisfy CAI > 0.8, with only O15263 showing a measurable CAI constraint penalty (0.046 kcal/mol). This minimal average penalty (1.354 → 1.358 kcal/mol, +0.004) demonstrates that accessibility optimization and codon adaptation are largely compatible objectives.

Accessibility-CAI Trade-off Landscape: Figures S2 and S3 visualize the complete optimization landscape from 1,364,736 combinations. The scatter plot uses stratified sampling to show the relationship between CAI and accessibility, with red vertical line marking the CAI = 0.8 threshold. The distribution histograms show accessibility ranges for each protein across all combinations.

Performance Comparison

Descriptively, the two best full-sequence ID3 variants achieve lower accessibility scores than the C10 restricted-window optimum by 0.425 kcal/mol (Amino.Sto.Soft: 0.929 vs. 1.354) and 0.419 kcal/mol (Codon.Sto.Soft: 0.935 vs. 1.354), while the mean of the top-10 ID3 variants is 0.983 kcal/mol, 0.371 kcal/mol below C10. Formal significance claims against C10 are taken from the complete Holm-Bonferroni matrix in Table S6: 9 of the 12 full-sequence ID3 variants significantly outperform C10 after correction, whereas Lagrangian.Det.Hard and the two soft Lagrangian variants do not. This comparison is cross-scope: the full-sequence ID3 variants optimize the full coding sequence, whereas C10 exhaustively searches the first 10 codons. Same-window interpretation should instead rely on the ID3 10-codon comparison, where ID3 10-codon is statistically indistinguishable from C10 for accessibility-only optimization and worse than C10-CAI in the CAI-constrained matched-window setting. For the CAI-constrained restricted-window optimum, C10 with CAI > 0.8 has mean 1.358 kcal/mol and ranks below all top-12 full-sequence ID3 variants in Table S2.

S2.2.5. Computational Cost Analysis

All timing measurements were conducted on the same hardware environment: NVIDIA H100 GPU on the miyabi HPC cluster. Each ID3 run performs 1,000 gradient-descent iterations; each GA/SA run performs 1,000 fitness evaluations (population_size = 20, num_generations = 49 for GA; max_iterations = 999 for SA). Timing is reported as wall-clock optimization time per protein.

Per-Protein Timing Comparison

Table S13 reports measured optimization time for each algorithm across all 12 proteins. We additionally report wall-clock timing for ID3 in access-only, CAI no-penalty, and CAI penalty settings, together with GA/SA baseline timing on the same H100 node (1,000 evaluations each).

Cost Structure Analysis

Optimization time scales approximately linearly with sequence length for all methods (Spearman $\rho \approx 0.98$, $p < 0.001$). The cost differential between methods arises from their computational structure:

- **GA/SA**: Table S13 reports mean per-protein times of 18.1 ± 18.6 s for GA and 17.8 ± 18.2 s for SA. Each evaluation calls DeepRaccess once on a discrete sequence. At 1,000 evaluations, the total cost is dominated by 1,000 forward passes through the FCN.
- **ID3 Access-Only**: Table S13 reports a mean per-protein time of 63.3 ± 57.7 s. Each gradient-descent iteration performs one differentiable forward pass with backpropagation. The higher cost per iteration stems from gradient computation; however, fewer iterations are required for convergence because gradients guide the search directly.
- **ID3 CAI-Joint**: Table S13 reports mean per-protein times of 452.2 ± 500.3 s without the penalty term and 440.0 ± 478.6 s with the penalty term. The CAI loss requires an additional codon probability computation per iteration. Because the CAI loss operates jointly over all codon positions, its gradient computation involves the full codon probability tensor, making these runs substantially slower than access-only mode. GA and SA do not support joint CAI optimization, so no matched-task quality baseline is available in this setting.

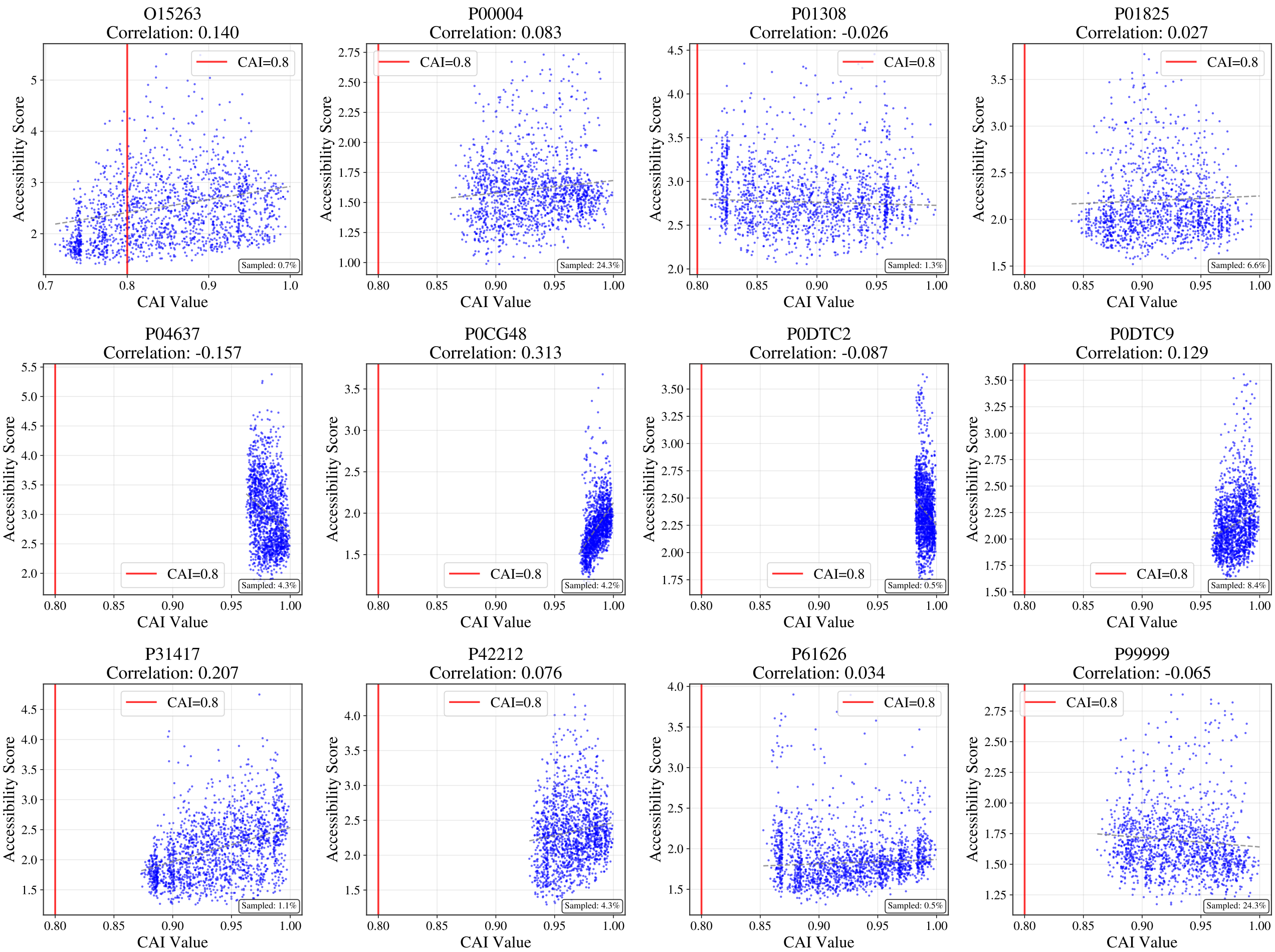

Fig. S2: Accessibility-CAI trade-off from C10 combinatorial exploration across 12 proteins. Each subplot shows a stratified sample representing the complete combinatorial space (total: 1,364,736 combinations). Red vertical line marks CAI = 0.8 threshold. Stratified sampling preserves distribution characteristics while maintaining visual clarity.

S2.2.6. CAI Integration Methods: Penalty vs. No-Penalty

Two orthogonal dimensions control CAI integration: (1) whether penalty-based joint loss is used during optimization, and (2) CAI-aware discretization during the evaluation phase (applied in all CAI experiments). Table S14 compares with-penalty vs. without-penalty configurations across all 12 base variants (n = 2,880 each).

Both CAI integration methods achieve statistically indistinguishable performance (difference = 0.005 kcal/mol, p = 0.661), with no significant differences across constraint types or base modes. Detailed ablation study and methodological comparison is provided in Section S9.

S2.2.7. Summary of Statistical Findings

1. **Constraint Mechanism Comparison**: Codon Profile and Amino Matching are statistically indistinguishable in this accessibility benchmark (difference ¡ 0.1%, $p = 0.922$, Cohen's $d = -0.005$). We interpret this as similar performance and partial redundancy in the tested setting, not as proof of universal equivalence. The two mechanisms remain mathematically distinct because Codon Profile directly parameterizes synonymous-codon simplexes, whereas Amino Matching uses a nucleotide-level sequence-preference interface followed by an amino-acid compatibility transformation.
2. **Lagrangian Underperformance**: Lagrangian Multiplier performs significantly worse than both Codon and Amino mechanisms (0.258-0.259 kcal/mol worse representing 26% degradation, large effect size $d \approx -0.9$, $p < 0.001$), consistent with optimization difficulty introduced by soft penalty-based constraint coupling.
3. **Top Variant Similarity**: Rank 1 (Amino.Sto.Soft, 0.929 kcal/mol) and Rank 2 (Codon.Sto.Soft, 0.935 kcal/mol) are statistically indistinguishable after Holm-Bonferroni correction (adjusted $p = 1.00$). More broadly, the top-8 ID3 variants form a statistically indistinguishable elite cluster in the complete pairwise matrix.
4. **Base Mode Performance**: Stochastic hard mode achieves best overall performance (0.991 kcal/mol) compared to other modes (Det.Hard: 1.014, Sto.Soft: 1.082, Det.Soft: 1.148), with significant differences confirmed by ANOVA ($F = 45.88$, $p < 0.001$).

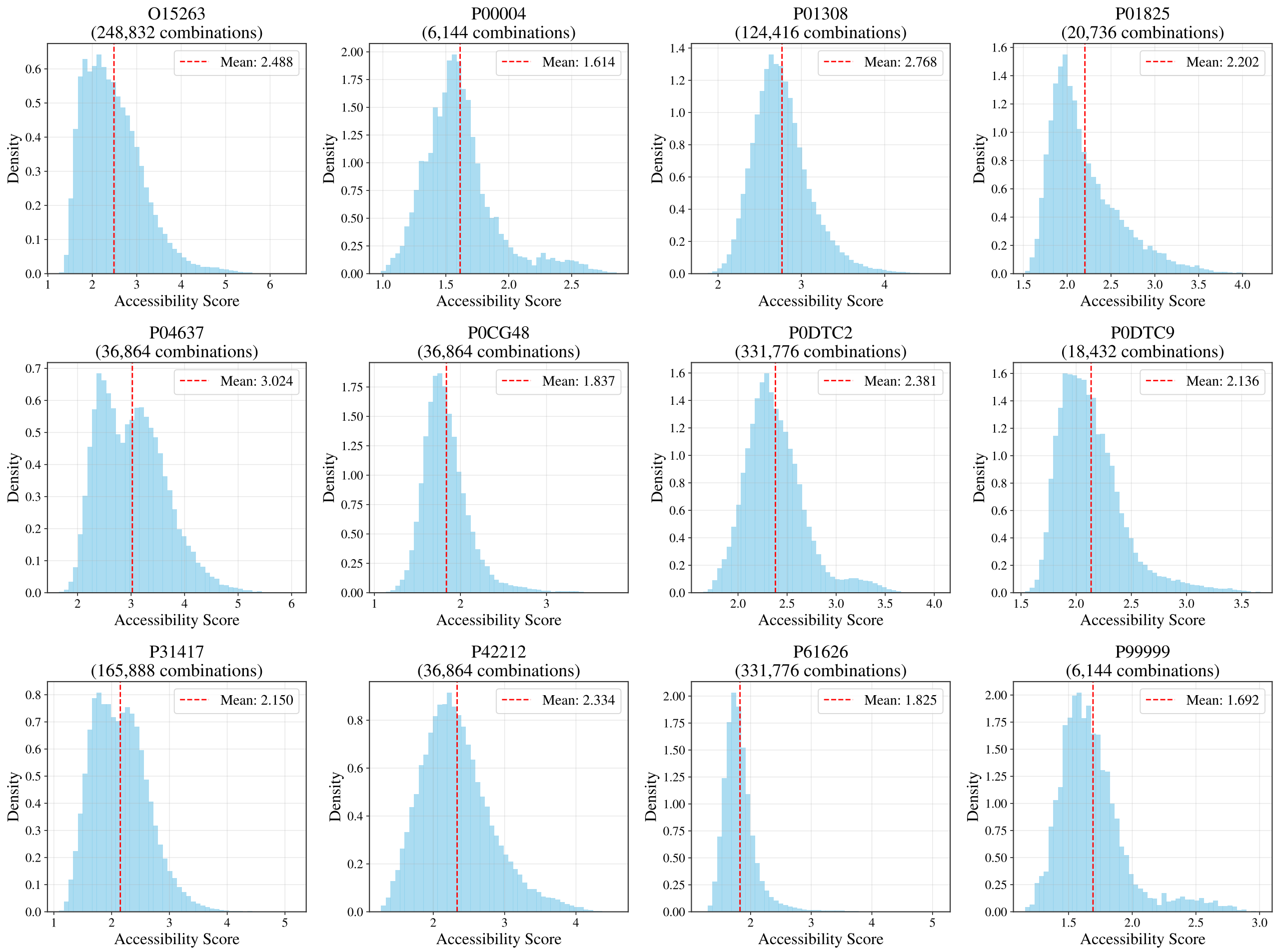

Fig. S3: Accessibility distributions from C10 combinatorial exploration across 12 proteins. Histograms show the complete accessibility landscape for all synonymous combinations of the first 10 codons (total: 1,364,736 combinations). Vertical lines indicate minimum accessibility values found for each protein.

5. **Framework Superiority over Baselines**: In the complete accessibility-only pairwise matrix, all 12 full-sequence ID3 variants significantly outperform both Genetic Algorithm and Simulated Annealing after Holm-Bonferroni correction.
6. **Full-Sequence Optimization Advantage**: Nine of the 12 full-sequence ID3 variants significantly outperform the C10 combinatorial baseline after Holm-Bonferroni correction, while ID3-10 remains statistically indistinguishable from C10 in the same 10-codon window (adjusted $p = 0.442$). Together, these results show that same-window gradient search approaches the exhaustive ceiling, whereas the leading full-sequence variants benefit from optimizing a larger sequence context where exhaustive enumeration is infeasible.

S3. Systematic Comparison with Prior Approaches

Table S15 provides a systematic comparison of existing approaches to the discrete-continuous optimization gap in RNA sequence design. We evaluate each approach across four key dimensions required for practical mRNA codon optimization: (1) high-quality gradient flow for efficient optimization, (2) compatibility with models that require discrete inputs, (3) systematic codon-level constraints for amino acid preservation, and (4) model-agnostic reusability without retraining.

Key distinctions. Continuous relaxation [Killoran et al., 2017] suffers from predictor pathology when models receive out-of-distribution probabilistic inputs. STE-based activation maximization [Linder and Seelig, 2021] enables gradient flow through discrete operations but lacks codon-level constraints for amino acid preservation. Differentiable folding [Krueger and Ward, 2025] provides exact gradients but is coupled to specific physics-based partition function models. The probabilistic lattice method [Dai et al., 2025] incorporates codon constraints via projection but remains coupled to ViennaRNA energy models. ID3 uniquely provides all four capabilities through its decoupled optimization-evaluation architecture and three complementary constraint mechanisms.

Table S13. Per-protein optimization time (seconds) on H100 GPU. ID3 times are summarized from production logs for each protein; GA/SA are from single benchmark runs (1,000 evaluations each). Sequence length in nucleotides. Mean row shows average across 12 proteins; ±std reflects protein-to-protein variability driven by sequence length.

Protein	Seq.Len (nt)	GA (s)	SA (s)	ID3 Access-Only mean (s)	ID3 CAI-Joint	
					No-Penalty (s)	Penalty (s)
O15263	192	6.65	6.09	22.5	100.5	103.6
P00004	315	6.11	6.12	30.3	150.2	149.7
P01308	330	6.23	6.15	30.5	175.5	175.3
P01825	348	6.22	6.12	30.7	186.4	185.4
P04637	1179	24.06	23.56	61.3	555.5	557.2
P0CG48	2055	35.95	35.28	114.1	966.7	965.7
P0DTC2	3819	71.47	70.07	228.3	1819.3	1729.0
P0DTC9	1257	24.05	23.54	70.3	586.9	562.5
P31417	396	6.26	6.15	34.8	194.3	187.2
P42212	714	12.25	11.95	56.6	323.7	310.3
P61626	444	11.98	11.89	41.1	217.2	208.1
P99999	315	6.19	6.11	39.5	150.8	145.8
**Mean**	–	**18.1**$_{\pm 18.6}$	**17.8**$_{\pm 18.2}$	**63.3**$_{\pm 57.7}$	**452.2**$_{\pm 500.3}$	**440.0**$_{\pm 478.6}$

Table S14. Penalty vs. No-Penalty CAI Integration Comparison

Method	Mean (kcal/mol)	Std	Cohen's d	p-value
Penalty-based	1.306	0.387	0.012	0.661
No-Penalty	1.301	0.390		
**By Constraint**: Codon Δ=+0.008, Amino Δ=-0.006, Lagrangian Δ=+0.011 (all p>0.05)				
**By Base Mode**: Det.Soft Δ=-0.008, Det.Hard Δ=+0.012, Sto.Soft Δ=+0.008, Sto.Hard Δ=+0.004 (all p>0.05)				

Table S15. Comparison of approaches to the discrete-continuous optimization gap in RNA design.

Approach	Gradient Flow	Discrete Input	Codon Constraint	Model Reuse
Continuous Relaxation [Killoran et al., 2017]	Biased	No	No	Limited
STE Activation Max. [Linder and Seelig, 2021]	Biased (STE)	Yes	No	Yes
Differentiable Folding [Krueger and Ward, 2025]	Exact	No	Implicit	No
Probabilistic Lattice [Dai et al., 2025]	Projected	Yes	Yes	No
**ID3 (this work)**	**Soft/STE**	**Yes**	**Yes (3 mechanisms)**	**Yes**

S4. Notation and Symbols

This section provides a comprehensive reference for all mathematical symbols used throughout the main paper and supplementary material, organized by functional categories.

Table S16. ID3 Framework Core Symbols

Symbol	Definition	Dimension
Θ	Learnable parameters (dimension varies by constraint)	See constraint-specific definitions
Θ_{codon}	Codon-level parameters (Codon Profile)	$\mathbb{R}^{\sum_j \lvert\mathcal{C}(y_j)\rvert}$
$\mathbf{P}$	RNA probability distribution (sequence profile)	$[0,1]^{L\times 4}$
$\mathbf{S}$	RNA sequence (discrete or soft)	$\{A,U,G,C\}^L$ or $[0,1]^{L\times 4}$
$\mathbf{P}_{\text{codon}}$	Codon probability distribution	$[0,1]^M$
T	Unified transformation function	$\Theta \mapsto \mathbf{S}$
Π	Parameter-to-probability transformation	$\Theta \mapsto \mathbf{P}$
Ψ	Probability-to-sequence transformation	$\mathbf{P} \mapsto \mathbf{S}$
σ	Temperature parameter	$\mathbb{R}^+$
$T^{\text{det.soft}}(\Theta)$	Deterministic soft transformation	$\Theta \mapsto \mathbf{S}$
$T^{\text{det.hard}}(\Theta)$	Deterministic hard transformation	$\Theta \mapsto \mathbf{S}$
$T^{\text{sto.soft}}(\Theta)$	Stochastic soft transformation	$\Theta \mapsto \mathbf{S}$
$T^{\text{sto.hard}}(\Theta)$	Stochastic hard transformation	$\Theta \mapsto \mathbf{S}$
T^{soft}	Generic soft transformation	$\mathbf{P} \mapsto \mathbf{S}$
T^{hard}	Generic hard transformation	$\mathbf{P} \mapsto \mathbf{S}$

Table S17. Biological Sequence Symbols

Symbol	Definition	Dimension
$\mathbf{y}$	Target amino acid sequence	$\{\text{AA}\}^M$
M	Number of amino acids (sequence length)	$\mathbb{N}$
L	RNA sequence length ($L = 3M$)	$\mathbb{N}$
$\mathcal{C}(y_j)$	Set of valid codons for amino acid y_j	$\{\text{codon}\}$
$\mathcal{E}[c]$	One-hot encoding of codon c	$\{0,1\}^{3\times 4}$
g	Gumbel noise	$\mathbb{R}$
$\theta_{j,c}$	Parameter for codon c at position j (Codon Profile)	$\mathbb{R}$
$\Theta^{(j)}$	RNA parameters for position j (Amino Matching)	$\mathbb{R}^{3\times 4}$

Table S18. Application-Specific Symbols

Symbol	Definition	Dimension
f_{model}	Pre-trained predictor (DeepRaccess)	$\mathbb{R}^L \mapsto \mathbb{R}$
f_{access}	RNA accessibility prediction function	$\mathbb{R}^L \mapsto \mathbb{R}$
$\mathcal{L}_{\text{access}}$	Accessibility loss function	$\mathbb{R}$
CAI	Codon Adaptation Index	$[0,1]$
ECAI	Expected CAI	$[0,1]$
w_c	CAI weight for codon c (see Table S2_additional)	$[0,1]$
γ	Interpolation parameter (binary search)	$[0,1]$
threshold	Target CAI threshold	$[0,1]$
Π_{codon}	Codon-specific probability function (Codon Profile)	$\Theta_{\text{codon}} \mapsto \mathbf{P}_{\text{codon}}$
Π_{amino}	Amino-based probability function (Amino Matching)	$\Theta \mapsto \mathbf{P}_{\text{codon}}$
$\text{Onehot}(\cdot)$	Vectorized one-hot encoding function	$\mathbf{P}_{\text{codon}} \mapsto \mathbf{S}$
$R_{\text{nuc}}(\cdot)$	Vectorized nucleotide reconstruction function	$\mathbf{P}_{\text{codon}} \mapsto \mathbf{S}$
$\text{soft\_embedding}^{(i)}$	Soft embedding at position i	$\mathbb{R}^d$
$\text{embedding}[\cdot]$	Trained embedding layer	$\mathbb{R}^d$
5'UTR_{70}	5' Untranslated Region (70 nt)	RNA sequence
3'UTR_{63}	3' Untranslated Region (63 nt)	RNA sequence
$\text{ATG} - 19 : +15$	Ribosome binding region for accessibility optimization	RNA region
CDS_{var}	Coding Sequence (variable length)	RNA sequence

Table S19. Optimization and Convergence Symbols

Symbol	Definition	Dimension
$\mathcal{L}$	General loss function	$\mathbb{R}$
$F(\Theta)$	Objective function for optimization	$\mathbb{R}$
∇	Gradient operator	Vector field
η	Learning rate	$\mathbb{R}^+$
λ	Lagrangian multiplier	$\mathbb{R}^+$
$\mathcal{C}(\mathbf{P})$	Constraint penalty function	$\mathbb{R}^+$
$L_{\text{composite}}$	Composite Lipschitz constant	$\mathbb{R}^+$
$L_{\mathcal{L}}$	Loss function Lipschitz constant	$\mathbb{R}^+$
B_{STE}	STE bias bound	$\mathbb{R}^+$
$\Delta_{\max}$	Maximum probability difference	$[0, 1]$
ϵ	Convergence tolerance	$\mathbb{R}^+$
k	Top-k position selection parameter	$\mathbb{N}$
potential_i	Improvement potential at position i	$[0, 1]$
score	Composite evaluation score	$[0, 1]$
$h(\cdot)$	Sequence hash function (duplicate detection)	$\mathbb{N} \mapsto$ String
$\alpha_{\text{CAI}}, \alpha_{\text{prob}}$	Score weighting parameters	$[0, 1]$

Table S20. ID3 Variant Notation (V40 Descriptive Naming)

Notation	Description
**Base Operational Modes**	
Det.Soft	Deterministic parameters with soft (continuous) output
Det.Hard	Deterministic parameters with hard (discrete) output
Sto.Soft	Stochastic parameters with soft (continuous) output
Sto.Hard	Stochastic parameters with hard (discrete) output
**Constraint Mechanisms (ordered by presentation in text)**	
Codon	Codon Profile Constraint variants
Amino	Amino Matching Softmax variants
Lagrangian	Lagrangian Multiplier variants
**Complete Variant Naming**	
Codon.Det.Soft	Codon Profile Constraint + Deterministic Soft
Codon.Det.Hard	Codon Profile Constraint + Deterministic Hard
Codon.Sto.Soft	Codon Profile Constraint + Stochastic Soft
Codon.Sto.Hard	Codon Profile Constraint + Stochastic Hard
Amino.Det.Soft	Amino Matching Softmax + Deterministic Soft
Amino.Det.Hard	Amino Matching Softmax + Deterministic Hard
Amino.Sto.Soft	Amino Matching Softmax + Stochastic Soft
Amino.Sto.Hard	Amino Matching Softmax + Stochastic Hard
Lagrangian.Det.Soft	Lagrangian Multiplier + Deterministic Soft
Lagrangian.Det.Hard	Lagrangian Multiplier + Deterministic Hard
Lagrangian.Sto.Soft	Lagrangian Multiplier + Stochastic Soft
Lagrangian.Sto.Hard	Lagrangian Multiplier + Stochastic Hard

Table S21. Additional Symbols for Convergence Analysis

Symbol	Definition	Dimension
Probability and Distribution Symbols		
$p_k^{(i)}$	Probability of nucleotide $k \in \{A, U, G, C\}$ at position i	$[0, 1]$
$\mathbf{P}_{\text{codon}}$	Codon probability vector	$[0, 1]^M$
$P_{\text{codon}_{j,c}}$	Probability of codon c at amino acid position j	$[0, 1]$
$\mathbf{P}^{(j)}$	Probability matrix for codon at position j	$[0, 1]^{3\times4}$
Optimization Bounds and Constants		
$\epsilon_{\min}$	Minimum probability bound (numerical stability)	$\mathbb{R}^+$
$\sigma_{\min}, \sigma_{\max}$	Temperature parameter bounds	$\mathbb{R}^+$
C_f	Lipschitz constant for function f_{model}	$\mathbb{R}^+$
$C_{\mathcal{L}}$	Lipschitz constant for loss function $\mathcal{L}$	$\mathbb{R}^+$
$C_{\text{composite}}$	Composite Lipschitz constant	$\mathbb{R}^+$
B	Upper bound for gradient magnitude	$\mathbb{R}^+$
B_{STE}	STE gradient bias bound: $\|g_{\text{STE}} - \nabla f\| \leq B_{\text{STE}}$	$\mathbb{R}^+$
C_{STE}	STE constant: $C_{\text{STE}} = C_{\mathcal{L}} \cdot C_f$	$\mathbb{R}^+$
$\Delta_{\max}$	Maximum probability difference in distribution	$[0, 1]$
Iteration and Convergence Symbols		
t	Iteration index	$\mathbb{N}$
N	Total number of iterations	$\mathbb{N}$
$\Theta^{(t)}$	Parameters at iteration t	$\mathbb{R}^n$
Θ^*	Optimal parameters at convergence	$\mathbb{R}^n$
Stochastic Optimization Symbols		
σ_G	Standard deviation of Gumbel noise	$\mathbb{R}^+$
$\xi^{(t)}$	Stochastic noise contribution to gradient at iteration t	$\mathbb{R}^n$
$\nabla F_{\text{det}}(\Theta)$	Deterministic gradient component	$\mathbb{R}^n$
Constraint Optimization Symbols		
μ	Strong convexity constant	$\mathbb{R}^+$
$g^{(t)}$	Subgradient of constraint at iteration t (Lagrangian)	$\mathbb{R}$
η_0	Initial learning rate	$\mathbb{R}^+$
η_t	Learning rate at iteration t	$\mathbb{R}^+$
D_{feas}	Diameter of feasible region	$\mathbb{R}^+$
Mathematical Operators		
δ_{ij}	Kronecker delta: 1 if $i = j$, 0 otherwise	$\{0, 1\}$
$\text{vec}(\mathbf{A})$	Vectorization: stack columns of matrix $\mathbf{A}$	Function
$\langle \mathbf{a}, \mathbf{b} \rangle$	Inner product of vectors $\mathbf{a}$ and $\mathbf{b}$	$\mathbb{R}$
CAI Optimization Symbols		
$\mathbf{w}$	CAI-optimal codon probability distribution	$[0, 1]^M$
w_c	CAI weight for codon c (relative adaptiveness)	$[0, 1]$
$\mathcal{S}_{\text{valid}}$	Valid amino-acid-preserving sequence space	Set
$\mathbf{v}_c$	Feature vector for codon c (Amino Matching)	$\mathbb{R}^{12}$
X_{ij}	Count of codon j for amino acid i in reference	$\mathbb{N}$
n_i	Number of synonymous codons for amino acid i	$\mathbb{N}$

S5. Mathematical Foundations and Assumptions

S5.1. Fundamental Assumptions

The convergence analysis of the ID3 framework relies on three fundamental assumptions that reflect the practical characteristics of pre-trained neural networks and RNA sequence optimization.

Assumption 1 (Pre-trained Model Local Properties) The pre-trained model f_{model} exhibits local regularity in the optimization region. Here, $\mathbf{S} = T(\Theta)$ denotes the model input, i.e., the generalized RNA sequence obtained from parameters Θ via transformation T.

- **Local Lipschitz continuity** [Nesterov, 2004]: There exists a constant $C_f > 0$ such that for any two inputs $\mathbf{S}_1, \mathbf{S}_2$ encountered during optimization:
$$|f_{\text{model}}(\mathbf{S}_1) - f_{\text{model}}(\mathbf{S}_2)| \leq C_f \|\mathbf{S}_1 - \mathbf{S}_2\| \quad (1)$$
- **Gradient boundedness**: The gradient magnitude is bounded: $\|\nabla f_{\text{model}}(\mathbf{S})\| \leq B < \infty$ for finite constant B.

Rationale: Neural networks composed of Lipschitz-continuous layers (e.g., linear layers, ReLU activations, convolutions) are themselves Lipschitz continuous, with the composite constant bounded by the product of per-layer constants [Goodfellow et al., 2016]. DeepRaccess is a fully convolutional network (FCN) using ReLU activations, dropout, and weight decay, which is therefore structurally consistent with the local-regularity assumption.

Empirical verification: The purpose of the empirical analysis is to test whether the local-regularity claim in Assumption 1 is numerically reasonable in the optimization region actually visited by ID3. To do so, two complementary input-space diagnostics are evaluated, and each is shown explicitly in Figure S4.

1. **Gradient metric (Figure S4a).** The first quantity is the input-gradient norm
$$G_t := \|\nabla_{\mathbf{S}} f_{\text{model}}(\mathbf{S}_t)\|. \quad (2)$$
This is the quantity plotted on the y-axis of Figure S4a. It measures infinitesimal local sensitivity of the predictor along the optimization trajectory.
2. **Finite-difference metric (Figure S4b).** Here, t indexes the optimization iteration, ρ the perturbation scale, and s the sampled perturbation replicate. Let i index sequence positions and $k \in \{1, 2, 3, 4\}$ the nucleotide channel. In the soft-mode trajectory analyzed here, each $\mathbf{S}_t$ is a row-wise nucleotide probability matrix. For each iteration t, perturbation scale ρ, and sample index s, we sample a zero-sum perturbation $\mathbf{u}_{t,s}^{(\rho)}$ satisfying
$$\sum_{k=1}^{4} \left(\mathbf{u}_{t,s}^{(\rho)}\right)_{i,k} = 0 \quad \text{for each sequence position } i, \quad (3)$$
and construct a perturbed input by repairing it to the row-wise probability simplex used by the soft input representation:
$$\tilde{\mathbf{S}}_{t,s}^{(\rho)} := \text{Repair}_{\mathcal{S}}(\mathbf{S}_t + \mathbf{u}_{t,s}^{(\rho)}). \quad (4)$$
The per-sample finite-difference ratio is then computed as
$$R_{t,s}^{(\rho)} := \frac{|f_{\text{model}}(\tilde{\mathbf{S}}_{t,s}^{(\rho)}) - f_{\text{model}}(\mathbf{S}_t)|}{\|\tilde{\mathbf{S}}_{t,s}^{(\rho)} - \mathbf{S}_t\|}. \quad (5)$$
For visualization, Figure S4b shows the per-iteration median across sampled perturbations,
$$\bar{R}_t^{(\rho)} := \text{median}_s \; R_{t,s}^{(\rho)}, \quad (6)$$
with a shaded band showing the smoothed full range across sampled perturbations at the same iteration. By construction, $R_{t,s}^{(\rho)}$ evaluates the left-hand side of the Assumption 1 inequality on a sampled nearby pair $(\mathbf{S}_t, \tilde{\mathbf{S}}_{t,s}^{(\rho)})$ in this local soft-input neighborhood, so it serves as a finite-perturbation approximation to the local Lipschitz quotient. In practice, 24 zero-sum perturbation-and-repair samples were used for each $\rho \in \{0.002, 0.005\}$ at every iteration.

Here $\text{Repair}_{\mathcal{S}}$ denotes the row-wise simplex repair operator used in this diagnostic: negative entries are clipped to zero and each row is renormalized to sum to one. This practical normalization step preserves the ambient row-wise simplex structure of the soft input, but it does not attempt to reparameterize the exact Amino Matching feasible manifold. The two diagnostics therefore correspond to the same assumption from complementary viewpoints: G_t measures differential local sensitivity, whereas $R_{t,s}^{(\rho)}$ measures finite local changes under nearby soft-input perturbations. If both remain numerically well behaved along the visited trajectory, then Assumption 1 is supported in the ambient input neighborhood explored by the optimizer.

In the representative O15263 case study (Amino.Sto.Soft, 1000 iterations), G_t was evaluated at every iteration, and $R_{t,s}^{(\rho)}$ was sampled with 24 zero-sum perturbation-and-repair samples for each $\rho \in \{0.002, 0.005\}$ at every iteration. The gradient metric remained small over

most of the trajectory: its average value was 0.655, and it stayed below 1.677 for 95% of the 1000 iterations, although the largest transient spike reached 5.557. The finite-difference metric showed the same overall pattern. Figure S4b visualizes the per-iteration median $\bar{R}_t^{(\rho)}$, while across all sampled perturbations from the full trajectory the underlying per-sample ratios $R_{t,s}^{(\rho)}$ had average value 0.0265, with 95% below 0.0836 and a largest observed ratio of 0.602.

To address whether the O15263 Amino.Sto.Soft diagnostic is representative, we expanded the same local-regularity diagnostics to the complete 12-protein × 12-variant matrix used in the main performance evaluation. The matrix covers all three constraint mechanisms (Amino Matching Softmax, Codon Profile Constraint, and Lagrangian Multiplier) and all four deterministic/stochastic and soft/hard variant classes for each of the 12 proteins. Each diagnostic trajectory used 1000 optimization iterations, 11 local-regularity checkpoints, and 8 zero-sum finite-difference perturbations for each $\rho \in \{0.002, 0.005\}$ at each checkpoint. As summarized in Table S22 and reported case by case in Table S23, the input-gradient norms and finite-difference ratios remain numerically bounded across the full matrix. Table S22 provides a compact per-variant summary, whereas Table S23 lists the unaggregated protein-by-variant diagnostic values used to assess the observed behavior.

These results should not be interpreted as a proof of Assumption 1 itself. Proving Assumption 1 would require establishing its bound uniformly over all admissible local input pairs in the relevant input domain. The present analysis instead evaluates representative optimization trajectories and finitely many nearby perturbation directions in their ambient row-wise simplex neighborhoods. Tables S22 and S23 therefore provide full-matrix trajectory-level empirical support for using Assumption 1 in the tested optimization regimes; Figure S4 remains a representative trajectory-level illustration rather than an additional full-matrix summary.

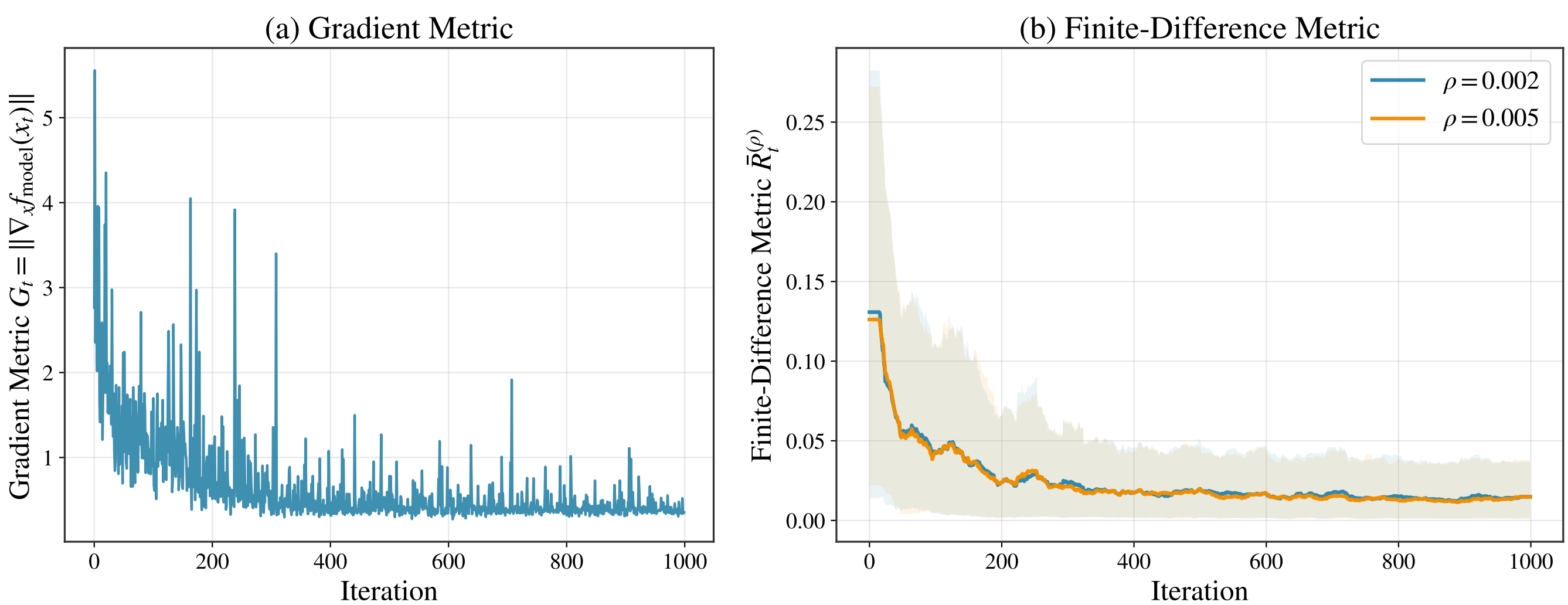

Fig. S4: Empirical support for local regularity of DeepRaccess in the visited optimization region (O15263, Amino.Sto.Soft, 1000 iterations). **(a)** Gradient metric $G_t = \|\nabla_{\mathbf{S}} f_{\text{model}}(\mathbf{S}_t)\|$, measured at all 1000 iterations. **(b)** Finite-difference metric summarized by the per-iteration median $\bar{R}_t^{(\rho)} = \text{median}_s\, R_{t,s}^{(\rho)}$, where t indexes optimization iterations, ρ the perturbation scale, and s the sampled perturbation replicate. Here, $R_{t,s}^{(\rho)} = |f_{\text{model}}(\tilde{\mathbf{S}}_{t,s}^{(\rho)}) - f_{\text{model}}(\mathbf{S}_t)|/\|\tilde{\mathbf{S}}_{t,s}^{(\rho)} - \mathbf{S}_t\|$ is the finite-difference ratio under zero-sum perturbations followed by row-wise simplex repair, with $\rho \in \{0.002, 0.005\}$, and the shaded band denotes the smoothed full range across sampled perturbations. The figure provides trajectory-level evidence for local regularity in the optimization region visited by ID3 rather than a uniform proof over the full admissible input domain.

Table S23: Case-level full-matrix local-regularity diagnostics for all 144 protein-by-variant cases. Each case used 1000 optimization iterations; G_t and finite-difference ratios were evaluated at the 11 diagnostic checkpoints $\{0, 1, 2, 5, 10, 20, 50, 100, 250, 500, 999\}$. The finite-difference sample count is $11 \times 2 \times 8 = 176$ for each case, corresponding to two perturbation radii and eight zero-sum perturbations per radius at each checkpoint. Median columns summarize within-trajectory checkpoint behavior, and maximum columns report the largest observed checkpoint or finite-difference-probe value for the case. These entries are the unaggregated case-level values summarized in Table S22.

Protein	Mechanism	Variant	Checkpoints	FD samples	G_t med.	G_t max	R med.	R max
O15263	Amino	Det.Soft	11	176	0.647	2.515	0.328	2.192
O15263	Amino	Det.Hard	11	176	1.645	10.15	0.459	3.930
O15263	Amino	Sto.Soft	11	176	1.427	4.578	0.383	3.621

Continued on next page

Table S23: Case-level full-matrix local-regularity diagnostics for all 144 protein-by-variant cases (continued).

Protein	Mechanism	Variant	Checkpoints	FD samples	G_t med.	G_t max	R med.	R max
O15263	Amino	Sto.Hard	11	176	2.596	5.482	0.495	2.622
O15263	Codon	Det.Soft	11	176	0.609	2.036	0.586	4.175
O15263	Codon	Det.Hard	11	176	1.050	1.924	0.553	3.387
O15263	Codon	Sto.Soft	11	176	1.375	4.691	0.403	3.185
O15263	Codon	Sto.Hard	11	176	2.026	4.106	0.442	3.595
O15263	Lagrangian	Det.Soft	11	176	1.952	6.507	0.272	2.575
O15263	Lagrangian	Det.Hard	11	176	3.294	7.985	0.471	3.570
O15263	Lagrangian	Sto.Soft	11	176	3.162	5.612	0.413	2.326
O15263	Lagrangian	Sto.Hard	11	176	2.220	5.425	0.575	3.754
P00004	Amino	Det.Soft	11	176	0.676	1.694	0.423	2.744
P00004	Amino	Det.Hard	11	176	0.997	2.624	0.314	2.555
P00004	Amino	Sto.Soft	11	176	0.804	2.122	0.433	2.578
P00004	Amino	Sto.Hard	11	176	1.145	1.931	0.513	3.785
P00004	Codon	Det.Soft	11	176	0.602	1.989	0.391	2.389
P00004	Codon	Det.Hard	11	176	0.929	2.233	0.518	2.472
P00004	Codon	Sto.Soft	11	176	0.838	2.109	0.466	3.912
P00004	Codon	Sto.Hard	11	176	1.201	4.082	0.448	2.670
P00004	Lagrangian	Det.Soft	11	176	1.059	6.899	0.257	3.776
P00004	Lagrangian	Det.Hard	11	176	0.520	1.402	0.393	2.376
P00004	Lagrangian	Sto.Soft	11	176	3.100	5.608	0.408	2.294
P00004	Lagrangian	Sto.Hard	11	176	1.184	2.749	0.424	2.627
P01308	Amino	Det.Soft	11	176	0.872	2.514	0.313	1.844
P01308	Amino	Det.Hard	11	176	1.102	2.271	0.357	2.204
P01308	Amino	Sto.Soft	11	176	1.535	5.136	0.566	3.299
P01308	Amino	Sto.Hard	11	176	1.468	2.828	0.421	2.381
P01308	Codon	Det.Soft	11	176	0.923	2.176	0.352	2.188
P01308	Codon	Det.Hard	11	176	1.389	4.353	0.365	2.168
P01308	Codon	Sto.Soft	11	176	2.030	3.096	0.402	2.561
P01308	Codon	Sto.Hard	11	176	1.674	3.303	0.397	2.333
P01308	Lagrangian	Det.Soft	11	176	2.141	6.579	0.428	3.397
P01308	Lagrangian	Det.Hard	11	176	1.193	3.958	0.359	3.416
P01308	Lagrangian	Sto.Soft	11	176	3.095	5.609	0.352	3.519
P01308	Lagrangian	Sto.Hard	11	176	1.574	2.313	0.447	3.063
P01825	Amino	Det.Soft	11	176	1.223	4.388	0.537	2.713
P01825	Amino	Det.Hard	11	176	0.831	2.203	0.341	2.485
P01825	Amino	Sto.Soft	11	176	1.184	3.537	0.555	2.383
P01825	Amino	Sto.Hard	11	176	2.315	3.701	0.491	2.789
P01825	Codon	Det.Soft	11	176	1.336	2.319	0.388	3.447
P01825	Codon	Det.Hard	11	176	1.182	3.616	0.384	3.410
P01825	Codon	Sto.Soft	11	176	1.971	4.322	0.451	3.274
P01825	Codon	Sto.Hard	11	176	1.483	2.300	0.407	2.836
P01825	Lagrangian	Det.Soft	11	176	2.009	6.917	0.270	3.464
P01825	Lagrangian	Det.Hard	11	176	0.923	1.608	0.385	2.407
P01825	Lagrangian	Sto.Soft	11	176	3.140	5.581	0.405	2.474
P01825	Lagrangian	Sto.Hard	11	176	1.739	3.955	0.503	3.327
P04637	Amino	Det.Soft	11	176	0.824	1.819	0.262	1.966
P04637	Amino	Det.Hard	11	176	0.918	2.075	0.358	2.664
P04637	Amino	Sto.Soft	11	176	1.392	2.871	0.440	2.458
P04637	Amino	Sto.Hard	11	176	2.058	6.574	0.380	1.966
P04637	Codon	Det.Soft	11	176	0.787	1.547	0.350	1.830
P04637	Codon	Det.Hard	11	176	1.312	2.006	0.356	2.377
P04637	Codon	Sto.Soft	11	176	1.392	2.808	0.467	2.573
P04637	Codon	Sto.Hard	11	176	1.553	2.488	0.399	2.871
P04637	Lagrangian	Det.Soft	11	176	0.880	6.044	0.212	3.676
P04637	Lagrangian	Det.Hard	11	176	0.867	2.276	0.319	2.294
P04637	Lagrangian	Sto.Soft	11	176	3.154	5.424	0.374	3.502
P04637	Lagrangian	Sto.Hard	11	176	1.939	2.768	0.416	2.348
P0CG48	Amino	Det.Soft	11	176	0.654	1.650	0.393	2.746
P0CG48	Amino	Det.Hard	11	176	0.797	0.902	0.419	1.977
P0CG48	Amino	Sto.Soft	11	176	0.832	1.197	0.374	2.212
P0CG48	Amino	Sto.Hard	11	176	0.814	1.148	0.423	3.402
P0CG48	Codon	Det.Soft	11	176	0.655	1.906	0.442	2.618
P0CG48	Codon	Det.Hard	11	176	0.849	1.139	0.321	1.926
P0CG48	Codon	Sto.Soft	11	176	0.756	1.858	0.460	1.926
P0CG48	Codon	Sto.Hard	11	176	0.825	1.384	0.360	3.337

Continued on next page

Table S23: Case-level full-matrix local-regularity diagnostics for all 144 protein-by-variant cases (continued).

Protein	Mechanism	Variant	Checkpoints	FD samples	G_t med.	G_t max	R med.	R max
P0CG48	Lagrangian	Det.Soft	11	176	1.433	6.552	0.341	3.179
P0CG48	Lagrangian	Det.Hard	11	176	0.823	1.671	0.343	1.883
P0CG48	Lagrangian	Sto.Soft	11	176	3.153	5.545	0.302	2.160
P0CG48	Lagrangian	Sto.Hard	11	176	0.937	2.086	0.429	2.606
P0DTC2	Amino	Det.Soft	11	176	1.062	2.070	0.391	2.488
P0DTC2	Amino	Det.Hard	11	176	1.371	1.615	0.408	2.799
P0DTC2	Amino	Sto.Soft	11	176	1.595	2.435	0.476	3.224
P0DTC2	Amino	Sto.Hard	11	176	1.594	2.499	0.422	2.614
P0DTC2	Codon	Det.Soft	11	176	1.131	2.107	0.385	2.059
P0DTC2	Codon	Det.Hard	11	176	1.939	2.733	0.492	3.892
P0DTC2	Codon	Sto.Soft	11	176	1.403	2.097	0.417	2.649
P0DTC2	Codon	Sto.Hard	11	176	1.234	1.986	0.427	2.399
P0DTC2	Lagrangian	Det.Soft	11	176	1.954	6.749	0.304	2.672
P0DTC2	Lagrangian	Det.Hard	11	176	1.213	2.641	0.449	2.367
P0DTC2	Lagrangian	Sto.Soft	11	176	3.196	5.451	0.303	2.033
P0DTC2	Lagrangian	Sto.Hard	11	176	1.645	3.155	0.420	2.290
P0DTC9	Amino	Det.Soft	11	176	0.565	5.800	0.381	3.442
P0DTC9	Amino	Det.Hard	11	176	0.799	3.115	0.374	2.106
P0DTC9	Amino	Sto.Soft	11	176	0.948	2.858	0.381	2.619
P0DTC9	Amino	Sto.Hard	11	176	1.019	2.318	0.425	2.347
P0DTC9	Codon	Det.Soft	11	176	0.589	7.339	0.393	2.626
P0DTC9	Codon	Det.Hard	11	176	0.853	2.482	0.347	2.597
P0DTC9	Codon	Sto.Soft	11	176	1.498	6.513	0.438	2.676
P0DTC9	Codon	Sto.Hard	11	176	0.893	3.127	0.465	2.453
P0DTC9	Lagrangian	Det.Soft	11	176	1.265	6.581	0.220	2.863
P0DTC9	Lagrangian	Det.Hard	11	176	0.898	7.185	0.387	3.123
P0DTC9	Lagrangian	Sto.Soft	11	176	3.098	5.543	0.398	2.348
P0DTC9	Lagrangian	Sto.Hard	11	176	0.837	2.713	0.462	3.218
P31417	Amino	Det.Soft	11	176	1.431	2.210	0.473	2.847
P31417	Amino	Det.Hard	11	176	0.929	2.853	0.384	2.106
P31417	Amino	Sto.Soft	11	176	1.270	3.582	0.341	2.264
P31417	Amino	Sto.Hard	11	176	1.671	2.689	0.412	2.179
P31417	Codon	Det.Soft	11	176	1.440	2.337	0.432	2.439
P31417	Codon	Det.Hard	11	176	1.195	2.301	0.481	2.677
P31417	Codon	Sto.Soft	11	176	1.492	2.945	0.481	2.970
P31417	Codon	Sto.Hard	11	176	1.935	2.604	0.356	1.988
P31417	Lagrangian	Det.Soft	11	176	1.071	6.832	0.212	3.308
P31417	Lagrangian	Det.Hard	11	176	1.293	2.758	0.501	3.146
P31417	Lagrangian	Sto.Soft	11	176	3.191	5.638	0.407	2.218
P31417	Lagrangian	Sto.Hard	11	176	1.457	6.203	0.386	2.774
P42212	Amino	Det.Soft	11	176	0.452	2.141	0.421	2.331
P42212	Amino	Det.Hard	11	176	0.574	1.392	0.529	3.303
P42212	Amino	Sto.Soft	11	176	1.146	3.029	0.383	2.694
P42212	Amino	Sto.Hard	11	176	0.872	3.495	0.397	2.522
P42212	Codon	Det.Soft	11	176	0.453	1.755	0.424	2.278
P42212	Codon	Det.Hard	11	176	0.825	3.149	0.437	2.908
P42212	Codon	Sto.Soft	11	176	1.184	2.599	0.422	2.419
P42212	Codon	Sto.Hard	11	176	1.117	3.047	0.385	2.635
P42212	Lagrangian	Det.Soft	11	176	0.879	6.604	0.328	3.305
P42212	Lagrangian	Det.Hard	11	176	0.645	1.187	0.433	2.646
P42212	Lagrangian	Sto.Soft	11	176	3.141	5.592	0.386	2.788
P42212	Lagrangian	Sto.Hard	11	176	0.936	4.184	0.449	1.954
P61626	Amino	Det.Soft	11	176	0.556	1.713	0.445	2.479
P61626	Amino	Det.Hard	11	176	0.854	1.334	0.386	2.167
P61626	Amino	Sto.Soft	11	176	1.358	2.429	0.371	2.361
P61626	Amino	Sto.Hard	11	176	1.046	5.332	0.482	2.320
P61626	Codon	Det.Soft	11	176	0.585	1.523	0.349	2.278
P61626	Codon	Det.Hard	11	176	0.909	1.778	0.402	3.291
P61626	Codon	Sto.Soft	11	176	1.045	5.571	0.396	3.687
P61626	Codon	Sto.Hard	11	176	0.991	2.603	0.331	2.446
P61626	Lagrangian	Det.Soft	11	176	0.969	6.659	0.297	2.509
P61626	Lagrangian	Det.Hard	11	176	1.199	4.105	0.493	3.965
P61626	Lagrangian	Sto.Soft	11	176	3.141	5.584	0.386	2.474
P61626	Lagrangian	Sto.Hard	11	176	1.026	2.268	0.326	2.402
P99999	Amino	Det.Soft	11	176	0.524	2.672	0.425	3.132

Continued on next page

Table S23: Case-level full-matrix local-regularity diagnostics for all 144 protein-by-variant cases (continued).

Protein	Mechanism	Variant	Checkpoints	FD samples	G_t med.	G_t max	R med.	R max
P99999	Amino	Det.Hard	11	176	1.042	3.185	0.407	2.523
P99999	Amino	Sto.Soft	11	176	0.978	2.459	0.403	2.869
P99999	Amino	Sto.Hard	11	176	1.303	3.553	0.535	3.186
P99999	Codon	Det.Soft	11	176	0.545	3.001	0.393	2.814
P99999	Codon	Det.Hard	11	176	0.828	1.892	0.452	3.241
P99999	Codon	Sto.Soft	11	176	1.042	4.032	0.344	2.080
P99999	Codon	Sto.Hard	11	176	1.218	4.866	0.454	2.725
P99999	Lagrangian	Det.Soft	11	176	0.926	6.626	0.252	3.776
P99999	Lagrangian	Det.Hard	11	176	0.544	2.227	0.476	2.480
P99999	Lagrangian	Sto.Soft	11	176	3.153	5.611	0.317	3.170
P99999	Lagrangian	Sto.Hard	11	176	1.354	3.515	0.498	2.901

Assumption 2 (Optimization Trajectory Boundedness) The probability distributions and temperature parameters remain within practical bounds during optimization. Here, $p_k^{(i)}$ denotes the probability of nucleotide $k \in \{A, U, G, C\}$ at position i in the RNA sequence, and σ is the temperature parameter controlling the softness of the distribution.

- **Probability bounds**: All nucleotide probabilities satisfy $p_k^{(i)} \in [\epsilon_{\min}, 1 - \epsilon_{\min}]$ where $\epsilon_{\min} > 0$ prevents probability collapse (i.e., ensures no probability approaches exactly 0 or 1).
- **Temperature bounds**: The temperature parameter remains bounded: $\sigma \in [\sigma_{\min}, \sigma_{\max}]$ with $0 < \sigma_{\min} \leq \sigma_{\max} < \infty$.

Practical significance: These bounds prevent numerical instabilities and ensure that the softmax transformation maintains meaningful probability distributions. In practice, $\epsilon_{\min} = 10^{-6}$ and $\sigma \in [0.1, 2.0]$ provide stable optimization behavior while preserving biological interpretability.

Assumption 3 (Loss Function Regularity) The loss function $\mathcal{L}$ possesses basic regularity properties:

- **Continuous differentiability**: $\mathcal{L}$ is continuously differentiable with well-defined gradients.
- **Lipschitz continuous gradients**: There exists a constant $C_{\mathcal{L}} > 0$ such that:

$$\|\nabla \mathcal{L}(z_1) - \nabla \mathcal{L}(z_2)\| \leq C_{\mathcal{L}} \|z_1 - z_2\| \tag{7}$$

  where z_1, z_2 are generic function inputs.

Note: Standard loss functions such as MSE, cross-entropy, and smooth approximations of ranking losses naturally satisfy these conditions.

S5.2. Optimization Objective

The general ID3 optimization problem takes the form:

$$\min_{\Theta} f_{\text{model}}(T(\Theta)) \tag{8}$$

where f_{model} is the fixed pre-trained model, $T(\Theta) = \Psi(\Pi(\theta))$ is the unified transformation function following the three-layer hierarchical architecture (Parameter Level → Probability Level → Output Level), and Θ represents optimizable parameters.

The objective function represents a composition of three functions:

$$F(\Theta) = \mathcal{L}(f_{\text{model}}(T(\Theta))) = (\mathcal{L} \circ f_{\text{model}} \circ T)(\Theta) \tag{9}$$

S6. Convergence Analysis

S6.1. Base Variant Convergence Analysis

S6.1.1. Det.Soft: Deterministic Soft Convergence

Det.Soft represents the most theoretically tractable variant, using deterministic softmax transformations without additional complexity.

Theorem 1 (Det.Soft Convergence) *Under Assumptions 1-3, gradient descent for Det.Soft mode with learning rate $\eta < 2/C_{composite}$ satisfies:*

$$\lim_{t \to \infty} \|\nabla F(\Theta^{(t)})\| = 0 \tag{10}$$

where t denotes the iteration index, $\Theta^{(t)}$ represents the parameters at iteration t, and the composite Lipschitz constant is:

$$C_{composite} = C_{\mathcal{L}} \cdot C_f \cdot \frac{1}{\sigma} \tag{11}$$

Table S22. Full-matrix local-regularity diagnostics across the complete 12-protein × 12-variant grid. Each case used 1000 optimization iterations, with local-regularity diagnostics evaluated at 11 checkpoints and 8 zero-sum finite-difference perturbations for each $\rho \in \{0.002, 0.005\}$ at each checkpoint. G_t denotes the input-gradient norm and R denotes the finite-difference ratio. Each row summarizes the 12 protein-specific cases for one ID3 variant: median columns report medians of the per-protein trajectory medians, whereas maximum columns report the largest per-protein trajectory maximum. Table S23 reports the corresponding unaggregated protein-by-variant case values. The table summarizes empirical trajectory-level support and is not intended as a uniform proof over all admissible input regions.

Mechanism	Variant	Proteins	G_t med.	G_t max	R med.	R max
Amino	Det.Soft	12	0.665	5.800	0.407	3.442
Amino	Det.Hard	12	0.923	10.15	0.385	3.930
Amino	Sto.Soft	12	1.227	5.136	0.393	3.621
Amino	Sto.Hard	12	1.385	6.574	0.424	3.785
Codon	Det.Soft	12	0.632	7.339	0.392	4.175
Codon	Det.Hard	12	0.989	4.353	0.419	3.892
Codon	Sto.Soft	12	1.384	6.513	0.430	3.912
Codon	Sto.Hard	12	1.226	4.866	0.403	3.595
Lagrangian	Det.Soft	12	1.168	6.917	0.271	3.776
Lagrangian	Det.Hard	12	0.910	7.985	0.413	3.965
Lagrangian	Sto.Soft	12	3.147	5.638	0.386	3.519
Lagrangian	Sto.Hard	12	1.405	6.203	0.438	3.754

Proof We establish convergence through a three-step analysis leveraging the composition structure $F = \mathcal{L} \circ f_{\text{model}} \circ T$.

Step 1: Lipschitz Constant Analysis. The composite Lipschitz constant is determined by the chain rule. For the gradient of the composite function:

$$\|\nabla F(\Theta)\| = \|\nabla \mathcal{L}(f_{\text{model}}(T(\Theta))) \cdot \nabla f_{\text{model}}(T(\Theta)) \cdot \nabla T(\Theta)\| \tag{12}$$

For the softmax transformation $T(\Theta)$, the gradient with respect to parameters has magnitude bounded by $\|\nabla T(\Theta)\| \leq \frac{1}{\sigma}$. This follows from the softmax gradient formula. Recall that $\theta_j^{(i)}$ denotes the parameter (logit) for nucleotide j at position i, and $p_k^{(i)}$ is the corresponding probability. The gradient of probability with respect to the parameter is:

$$\frac{\partial p_k^{(i)}}{\partial \theta_j^{(i)}} = \frac{1}{\sigma}[p_k^{(i)}(\delta_{kj} - p_j^{(i)})] \tag{13}$$

where δ_{kj} is the Kronecker delta function ($\delta_{kj} = 1$ if $k = j$, and $\delta_{kj} = 0$ otherwise). The bound follows from $|p_k^{(i)}(\delta_{kj} - p_j^{(i)})| \leq p_k^{(i)} \leq 1$.

Combining with Assumptions 1 and 3, the composite Lipschitz constant becomes:

$$C_{\text{composite}} = C_{\mathcal{L}} \cdot C_f \cdot \frac{1}{\sigma} \tag{14}$$

Step 2: Descent Lemma Application. For the $C_{\text{composite}}$-smooth function F, the standard descent lemma gives:

$$F(\Theta^{(t+1)}) \leq F(\Theta^{(t)}) - \frac{\eta}{2}\|\nabla F(\Theta^{(t)})\|^2 \tag{15}$$

provided $\eta < 2/C_{\text{composite}}$. This inequality holds for gradient descent updates $\Theta^{(t+1)} = \Theta^{(t)} - \eta \nabla F(\Theta^{(t)})$.

Step 3: Telescoping Analysis and Convergence. Summing the descent inequality over iterations $t = 0, \ldots, N-1$:

$$\sum_{t=0}^{N-1} \|\nabla F(\Theta^{(t)})\|^2 \leq \frac{2}{\eta}[F(\Theta^{(0)}) - F(\Theta^{(N)})] \tag{16}$$

Since F is bounded below (the loss function $\mathcal{L}$ has a lower bound by assumption) and $F(\Theta^{(0)})$ is finite, the right-hand side is bounded. Therefore, $\sum_{t=0}^{\infty} \|\nabla F(\Theta^{(t)})\|^2 < \infty$, which implies:

$$\lim_{t \to \infty} \|\nabla F(\Theta^{(t)})\| = 0 \tag{17}$$

□

S6.1.2. Sto.Soft: Stochastic Soft Convergence

Sto.Soft extends Det.Soft by adding Gumbel noise [Jang et al., 2017] for exploration, introducing stochastic elements while maintaining the core convergence structure.

Theorem 2 (Sto.Soft Stochastic Convergence) *With Gumbel noise, Sto.Soft mode satisfies:*

$$E[\|\nabla F(\Theta^{(t)})\|^2] \to 0 \text{ as } t \to \infty \tag{18}$$

Proof We analyze stochastic convergence through gradient decomposition and expectation bounds.

Step 1: Gradient Decomposition. The stochastic gradient can be decomposed as:

$$\nabla F(\Theta^{(t)}) = \nabla F_{\text{det}}(\Theta^{(t)}) + \xi^{(t)} \tag{19}$$

where $\nabla F_{\text{det}}(\Theta^{(t)})$ is the deterministic gradient (without noise), and $\xi^{(t)}$ represents the stochastic noise contribution to the gradient at iteration t from Gumbel sampling. This noise is zero-mean ($E[\xi^{(t)}] = 0$) with bounded variance $E[\|\xi^{(t)}\|^2] \leq \sigma_G^2$, where σ_G denotes the standard deviation of the Gumbel noise.

Step 2: Expected Descent Inequality. Applying the descent lemma to the expected objective and using the bias-variance decomposition:

$$\begin{aligned} E[F(\Theta^{(t+1)})] &\leq E[F(\Theta^{(t)})] - \eta E[\langle \nabla F(\Theta^{(t)}), \nabla F_{\text{det}}(\Theta^{(t)})\rangle] \\ &\quad + \frac{\eta^2 C_{\text{composite}}}{2} E[\|\nabla F(\Theta^{(t)})\|^2] \\ &= E[F(\Theta^{(t)})] - \eta E[\|\nabla F_{\text{det}}(\Theta^{(t)})\|^2] \\ &\quad + \frac{\eta^2 C_{\text{composite}}}{2} (E[\|\nabla F_{\text{det}}(\Theta^{(t)})\|^2] + \sigma_G^2) \end{aligned} \tag{20}$$

where we used $E[\langle \nabla F_{\text{det}}(\Theta^{(t)}), \xi^{(t)}\rangle] = 0$ due to independence.

Step 3: Convergence in Expectation. Rearranging the inequality:

$$\begin{aligned} E[\|\nabla F_{\text{det}}(\Theta^{(t)})\|^2] &\leq \frac{2}{\eta(2 - \eta C_{\text{composite}})} [E[F(\Theta^{(t)})] - E[F(\Theta^{(t+1)})]] \\ &\quad + \frac{\eta C_{\text{composite}} \sigma_G^2}{2 - \eta C_{\text{composite}}} \end{aligned} \tag{21}$$

For learning rate $\eta < 2/C_{\text{composite}}$, telescoping over iterations and using boundedness of F yields:

$$\sum_{t=0}^{\infty} E[\|\nabla F_{\text{det}}(\Theta^{(t)})\|^2] < \infty \tag{22}$$

Therefore, $E[\|\nabla F(\Theta^{(t)})\|^2] \to 0$ as $t \to \infty$. □

S6.1.3. Det.Hard and Sto.Hard: Straight-Through Estimator Variants

The Straight-Through Estimator (STE) [Bengio et al., 2013] is a gradient approximation technique used when the forward pass involves discrete operations (e.g., argmax, sampling) that are non-differentiable. In the ID3 framework:

- **Forward pass**: Use discrete/hard sequences (e.g., via argmax or Gumbel sampling with discretization)
- **Backward pass**: Compute gradients as if the forward pass used soft probabilities (ignoring the discretization)

This approximation introduces a gradient bias B_{STE}, which affects convergence properties.

Theorem 3 (Straight-Through Estimator Convergence Under Local Strong Convexity and Smoothness) *Let $f : \mathbb{R}^n \to \mathbb{R}$ be continuously differentiable. Assume that there exists a convex neighborhood U of a local minimizer x^* such that f is μ-strongly convex and L-smooth on U. Suppose that, for all $x \in U$, the Straight-Through Estimator (STE) produces a gradient estimate satisfying $\|g_{STE}(x) - \nabla f(x)\| \leq B_{STE}$. Assume that the iterates of the biased update*

$$x^{(t+1)} = x^{(t)} - \eta g_{STE}(x^{(t)}) \tag{23}$$

remain in U, and that the step size satisfies $0 < \eta \leq 1/L$. Then

$$\|x^{(t+1)} - x^*\| \leq (1 - \eta\mu)\|x^{(t)} - x^*\| + \eta B_{STE}, \tag{24}$$

and consequently

$$\limsup_{t\to\infty} \|x^{(t)} - x^*\| \leq \frac{B_{STE}}{\mu}. \tag{25}$$

Proof Let the STE error be

$$e^{(t)} := g_{\text{STE}}(x^{(t)}) - \nabla f(x^{(t)}), \tag{26}$$

so that $\|e^{(t)}\| \leq B_{\text{STE}}$ for all iterates in U. Since x^* is a local minimizer of a continuously differentiable function, $\nabla f(x^*) = 0$.

Using the update rule and the triangle inequality, we obtain

$$\begin{aligned}\|x^{(t+1)} - x^*\| &= \|x^{(t)} - x^* - \eta\nabla f(x^{(t)}) - \eta e^{(t)}\| \\ &\leq \|x^{(t)} - x^* - \eta(\nabla f(x^{(t)}) - \nabla f(x^*))\| + \eta\|e^{(t)}\|.\end{aligned} \tag{27}$$

Because f is μ-strongly convex and L-smooth on U, and $0 < \eta \leq 1/L$, the gradient descent map is contractive on U:

$$\|x - x^* - \eta(\nabla f(x) - \nabla f(x^*))\| \leq (1 - \eta\mu)\|x - x^*\|, \qquad x \in U. \tag{28}$$

Therefore,

$$\|x^{(t+1)} - x^*\| \leq (1 - \eta\mu)\|x^{(t)} - x^*\| + \eta B_{\text{STE}}. \tag{29}$$

Iterating this recursion yields

$$\|x^{(t)} - x^*\| \leq (1 - \eta\mu)^t\|x^{(0)} - x^*\| + \eta B_{\text{STE}} \sum_{k=0}^{t-1}(1 - \eta\mu)^k. \tag{30}$$

Since $\sum_{k=0}^{t-1}(1 - \eta\mu)^k \leq 1/(\eta\mu)$, we obtain

$$\|x^{(t)} - x^*\| \leq (1 - \eta\mu)^t\|x^{(0)} - x^*\| + \frac{B_{\text{STE}}}{\mu}. \tag{31}$$

Taking $\limsup$ as $t \to \infty$ gives

$$\limsup_{t\to\infty} \|x^{(t)} - x^*\| \leq \frac{B_{\text{STE}}}{\mu}, \tag{32}$$

which shows convergence to an $O(B_{\text{STE}})$ neighborhood of the local minimizer rather than exact convergence. □

This fundamental result leads to the following extensions:

Corollary 4 (Det.Hard Convergence) *Det.Hard (STE + Deterministic) satisfies:*

$$\textit{Det.Soft: } \|\nabla F(\Theta^*)\| = 0 \Rightarrow \textit{Det.Hard: } \|\nabla F(\Theta^*)\| \leq B_{STE} \tag{33}$$

Corollary 5 (Sto.Hard Convergence) *Sto.Hard (STE + Stochastic) satisfies:*

$$E[\|\nabla F(\Theta^*)\|] \leq \sqrt{B_{STE}^2 + \sigma_G^2} \tag{34}$$

The STE bias bound quantifies the error introduced by using soft gradients while discretizing the forward pass. At the symbolic level, we write it in terms of the soft-hard input discrepancy:

$$B_{\text{STE}} \leq C_{\text{STE}} \cdot \delta_{\text{SH}}(\Theta), \qquad \delta_{\text{SH}}(\Theta) := \|T_{\text{soft}}(\Theta) - T_{\text{hard}}(\Theta)\| \tag{35}$$

where:

- B_{STE} is the maximum difference between the biased STE gradient and the true gradient: $\|g_{\text{STE}}(x) - \nabla f(x)\| \leq B_{\text{STE}}$
- C_{STE} is a constant depending on the Lipschitz constants of the model f_{model} and loss function $\mathcal{L}$ (specifically, $C_{\text{STE}} = C_{\mathcal{L}} \cdot C_f$)
- $\delta_{\text{SH}}(\Theta)$ is the soft-hard input discrepancy induced by the relaxation and discretization
- σ is the temperature parameter controlling the sharpness of the soft approximation

Theorem 3 treats B_{STE} as an abstract local bias bound. To connect this quantity to the ID3 pipeline, we next derive a symbolic discrepancy-form expression for B_{STE} in terms of the soft-hard input mismatch.

In all experiments reported in this study, we fixed the relaxation temperature at $\sigma = 1.0$ and did not use temperature annealing. The STE bias reflects the mismatch between the soft path used for gradient computation and the hard path used for discrete evaluation. Lower σ typically makes T_{soft} closer to T_{hard}, but it also sharpens the softmax and can increase gradient sensitivity through the Jacobian scaling. We therefore do not claim a monotonic dependence of the overall STE bias on σ from the symbolic derivation alone.

Symbolic derivation of the STE bias bound.
The STE gradient bias is defined as the difference between the STE gradient estimate and the true gradient:

$$B_{\text{STE}} = \|g_{\text{STE}}(\Theta) - \nabla F(\Theta)\| \tag{36}$$

Here,

$$F(\Theta) := \mathcal{L}(f_{\text{model}}(T_{\text{hard}}(\Theta))) \tag{37}$$

denotes the hard-objective composed with the discrete input used in the forward pass, so $\nabla F(\Theta)$ is the true gradient of that hard objective with respect to Θ. By contrast, $g_{\text{STE}}(\Theta)$ is the surrogate gradient actually used to update Θ in the STE pipeline: the forward pass evaluates the hard/discrete input $T_{\text{hard}}(\Theta)$, while the backward pass substitutes the derivative of the soft transformation $T_{\text{soft}}(\Theta)$.

Step 1: Formal chain rule decomposition. At a symbolic level, the gradient mismatch through the full pipeline can be written as:

$$g_{\text{STE}} - \nabla F = \nabla \mathcal{L} \cdot \nabla f_{\text{model}} \cdot [\nabla T_{\text{soft}} - \nabla T_{\text{hard}}] \tag{38}$$

Here the hard-path term is only formal: because T_{hard} is a discrete transformation, it does not provide a usable pathwise derivative for backpropagation. STE therefore substitutes ∇T_{soft} as a surrogate, and this surrogate substitution is the source of the bias.

Step 2: Bounding via Lipschitz constants. The bias is bounded by the product of the Lipschitz constants of $\mathcal{L}$ and f_{model} (from Assumptions 1 and 3) and the discrepancy between soft and hard inputs:

$$\|g_{\text{STE}} - \nabla F\| \leq C_{\mathcal{L}} \cdot C_f \cdot \|T_{\text{soft}}(\Theta) - T_{\text{hard}}(\Theta)\| \tag{39}$$

Step 3: Soft-hard discrepancy form. Let

$$\delta_{\text{SH}}(\Theta) := \|T_{\text{soft}}(\Theta) - T_{\text{hard}}(\Theta)\|. \tag{40}$$

Combining with Step 2 gives

$$B_{\text{STE}} \leq C_{\mathcal{L}} \cdot C_f \cdot \delta_{\text{SH}}(\Theta) = C_{\text{STE}} \cdot \delta_{\text{SH}}(\Theta). \tag{41}$$

This form keeps the symbolic bound aligned with the soft-hard mismatch that is directly measured in the empirical diagnostics below. We do not further reduce $\delta_{\text{SH}}(\Theta)$ to a temperature-monotone closed form here, because such a reduction would require additional assumptions on the logits and margin structure.

Empirical validation. In the practical full-sequence setting, the exact hard-objective gradient $\nabla F(\Theta)$ is not directly observable, because the hard forward path contains discrete argmax/one-hot decoding and backpropagation through that exact hard objective is zero/undefined almost everywhere. Any numerical replacement would therefore introduce an additional surrogate of its own. We therefore focus on two measurable diagnostics on the representative O15263 trajectory (Amino.Sto.Soft, 1000 iterations) and further evaluate the same STE-soft discrepancy diagnostics across the full 12-protein × 12-variant matrix summarized in Table S24.

For empirical diagnostics at iteration t, we denote the instantiated soft and hard inputs by

$$\mathbf{S}_{\text{soft},t} := T_{\text{soft}}(\Theta_t), \qquad \mathbf{S}_{\text{hard},t} := T_{\text{hard}}(\Theta_t). \tag{42}$$

The first diagnostic is the parameter-space discrepancy

$$\Delta_\Theta(t) := \|g_{\text{STE}}(\Theta_t) - g_{\text{soft}}(\Theta_t)\|, \tag{43}$$

where

$$g_{\text{soft}}(\Theta) := \nabla_\Theta \mathcal{L}(f_{\text{model}}(T_{\text{soft}}(\Theta))). \tag{44}$$

Its average value is 0.206. Across the 1000 sampled iterations, 95% of the observed discrepancies are below 0.574, and $\Delta_\Theta(t)$ tracks the soft-hard input gap $\|\mathbf{S}_{\text{soft},t} - \mathbf{S}_{\text{hard},t}\|$ with Pearson correlation 0.741. Figure S5 visualizes this parameter-space diagnostic.

The second diagnostic is the input-space discrepancy

$$\Delta_S(t) := \|\nabla_{\mathbf{S}} f_{\text{model}}(\mathbf{S}_{\text{hard},t}) - \nabla_{\mathbf{S}} f_{\text{model}}(\mathbf{S}_{\text{soft},t})\|, \tag{45}$$

which we compare against the theory-motivated local upper-bound surrogate

$$\hat{U}_S(t) := \hat{L}_{\text{local}}(t)\|\mathbf{S}_{\text{hard},t} - \mathbf{S}_{\text{soft},t}\|. \tag{46}$$

This surrogate is the measurable local analogue of the symbolic discrepancy-form bound above: it replaces the unknown global constant by a local finite-difference estimate and uses the observed soft-hard input gap. Here, $\hat{L}_{\text{local}}(t)$ is estimated from zero-sum finite-difference probes of the input gradient around both $\mathbf{S}_{\text{soft},t}$ and $\mathbf{S}_{\text{hard},t}$, using the same row-wise simplex repair operator defined in Assumption 1, and the larger local estimate is retained.

Across the 1000 sampled iterations, $\Delta_S(t)$ has average value 1.950, 95% of the observed discrepancies are below 4.309, and the largest observed discrepancy is 9.484. In all 1000 iterations, $\Delta_S(t)$ remained below $\hat{U}_S(t)$. The observed/surrogate ratio has mean 0.064, median 0.054, and maximum 0.396, indicating that the local surrogate is conservative even in the largest-ratio case. Figure S6 visualizes this bound-style diagnostic. We therefore present it as a local upper-bound surrogate rather than a strict global empirical proof of the exact hard-objective gradient bias.

We further extended the STE discrepancy diagnostics to the same complete 12-protein × 12-variant matrix used for the local-regularity analysis. For each case, the local surrogate bound was evaluated at the same 11 parameter checkpoints, and we compared both the STE-soft parameter-gradient discrepancy and the input-space discrepancy against the local upper-bound surrogate. Table S24 summarizes the resulting discrepancies and surrogate coverage by variant, and Table S25 reports the corresponding unaggregated protein-by-variant values. Across the full diagnostic matrix, the diagnostics remain consistent with the local discrepancy-bound interpretation. We emphasize that this remains a surrogate-based validation because the exact hard-path gradient is not directly observable for the non-differentiable argmax/one-hot transformation.

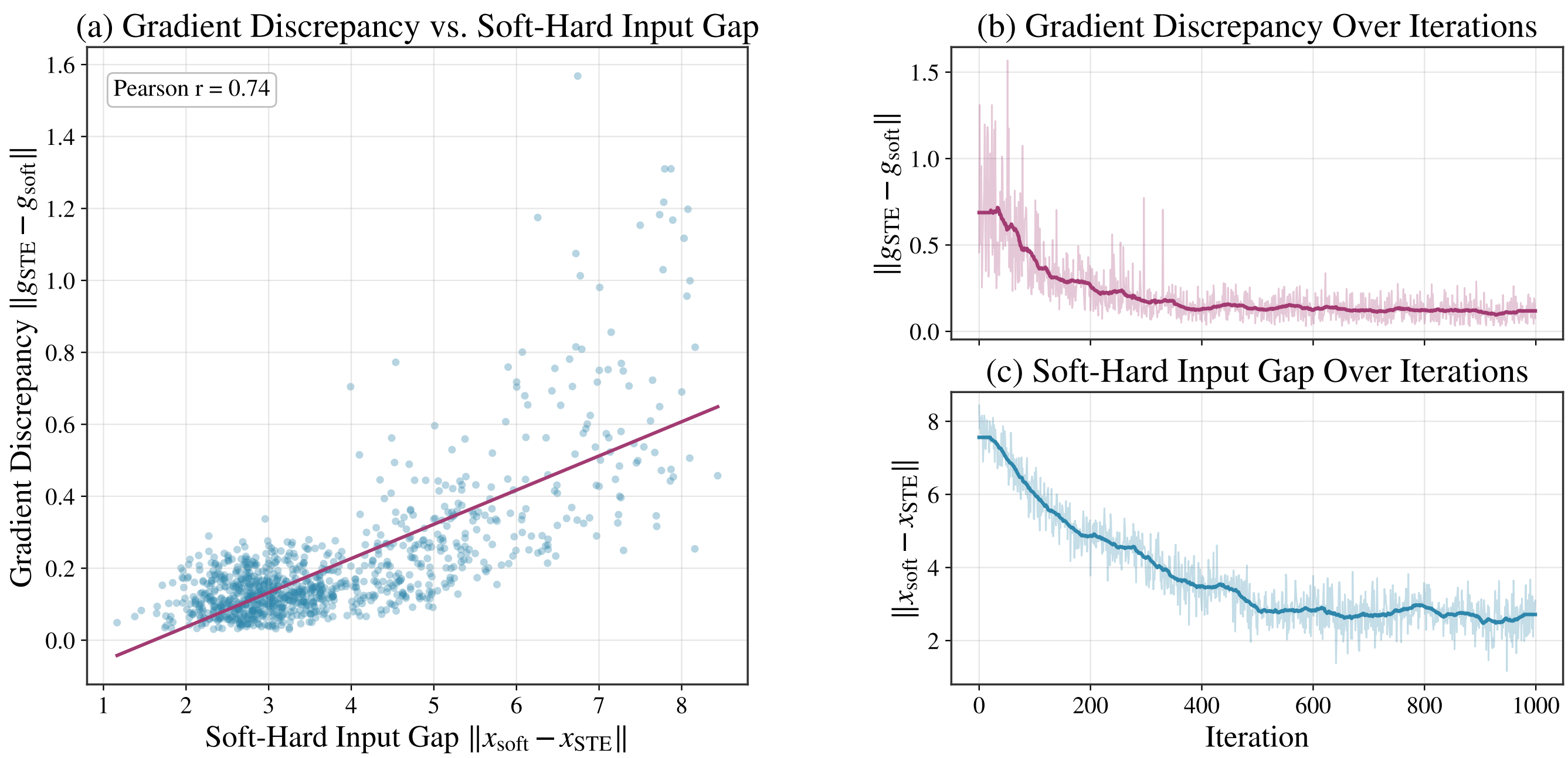

Fig. S5: Empirical STE discrepancy analysis in parameter space (O15263, Amino.Sto.Soft, 1000 iterations). **(a)** Parameter-space discrepancy $\|g_{\text{STE}} - g_{\text{soft}}\|$ versus the soft-hard input gap $\|\mathbf{S}_{\text{soft}} - \mathbf{S}_{\text{hard}}\|$, showing a clear positive relationship (Pearson $r = 0.741$). **(b)** Parameter-space discrepancy over iterations. **(c)** Soft-hard input gap over iterations. These results show that the STE update discrepancy is structured and remains bounded in the tested regime.

Table S24. Full-matrix STE discrepancy diagnostics across the complete 12-protein $\times$ 12-variant grid. For each case, the STE-soft parameter-gradient discrepancy Δ_Θ and the input-space discrepancy Δ_S were evaluated at the same 11 checkpoints used for the local-regularity matrix. Coverage denotes the fraction of checkpoints for which $\Delta_S \leq \widehat{U}_S$, where $\widehat{U}_S$ is the local upper-bound surrogate, and Max ratio denotes the maximum observed $\Delta_S/\widehat{U}_S$ value within the row. The table summarizes empirical support for the local discrepancy-bound interpretation without implying a global proof over all variants, proteins, or input regions.

Mechanism	Variant	Cases	Δ_Θ med.	Δ_Θ max	Δ_S max	Coverage	Max ratio
Amino	Det.Soft	12	0.177	2.338	11.58	100%	0.059
Amino	Det.Hard	12	0.231	2.338	11.58	100%	0.059
Amino	Sto.Soft	12	0.210	1.388	6.171	100%	0.034
Amino	Sto.Hard	12	0.236	1.444	6.588	100%	0.025
Codon	Det.Soft	12	0.115	1.250	7.716	100%	0.025
Codon	Det.Hard	12	0.192	1.253	7.716	100%	0.029
Codon	Sto.Soft	12	0.169	0.957	6.490	100%	0.023
Codon	Sto.Hard	12	0.199	0.983	6.630	100%	0.030
Lagrangian	Det.Soft	12	0.649	2.301	9.366	100%	0.028
Lagrangian	Det.Hard	12	0.960	2.495	10.67	100%	0.017
Lagrangian	Sto.Soft	12	0.689	1.451	6.735	100%	0.022
Lagrangian	Sto.Hard	12	0.786	1.734	7.791	100%	0.014

Table S25: Case-level full-matrix STE discrepancy diagnostics for all 144 protein-by-variant cases. Each case used 1000 optimization iterations, with STE discrepancy diagnostics evaluated at the 11 checkpoints $\{0, 1, 2, 5, 10, 20, 50, 100, 250, 500, 999\}$. Δ_Θ denotes the STE-soft parameter-gradient discrepancy, Δ_S denotes the input-space discrepancy, Coverage denotes the fraction of checkpoints satisfying $\Delta_S \leq \widehat{U}_S$, and Max ratio denotes the largest observed $\Delta_S/\widehat{U}_S$ value for the case. These entries are the unaggregated case-level values summarized in Table S24.

Protein	Mechanism	Variant	Checkpoints	Δ_Θ med.	Δ_Θ max	Δ_S max	Coverage	Max ratio
O15263	Amino	Det.Soft	11	0.180	2.338	11.58	100%	0.059
O15263	Amino	Det.Hard	11	0.490	2.338	11.58	100%	0.059
O15263	Amino	Sto.Soft	11	0.441	1.388	6.171	100%	0.034
O15263	Amino	Sto.Hard	11	0.577	1.444	6.588	100%	0.025
O15263	Codon	Det.Soft	11	0.153	0.436	3.150	100%	0.022

Continued on next page

Table S25: Case-level full-matrix STE discrepancy diagnostics for all 144 protein-by-variant cases (continued).

Protein	Mechanism	Variant	Checkpoints	Δ_Θ med.	Δ_Θ max	Δ_S max	Coverage	Max ratio
O15263	Codon	Det.Hard	11	0.160	0.240	2.256	100%	0.024
O15263	Codon	Sto.Soft	11	0.168	0.556	4.353	100%	0.023
O15263	Codon	Sto.Hard	11	0.355	0.647	5.210	100%	0.030
O15263	Lagrangian	Det.Soft	11	0.836	2.301	9.366	100%	0.028
O15263	Lagrangian	Det.Hard	11	1.341	2.301	9.366	100%	0.017
O15263	Lagrangian	Sto.Soft	11	0.927	1.287	6.034	100%	0.022
O15263	Lagrangian	Sto.Hard	11	0.963	1.240	5.759	100%	0.010
P00004	Amino	Det.Soft	11	0.127	0.316	2.728	100%	0.025
P00004	Amino	Det.Hard	11	0.144	0.282	2.212	100%	0.017
P00004	Amino	Sto.Soft	11	0.143	0.332	2.427	100%	0.008
P00004	Amino	Sto.Hard	11	0.168	0.345	2.554	100%	0.016
P00004	Codon	Det.Soft	11	0.039	0.231	2.280	100%	0.020
P00004	Codon	Det.Hard	11	0.132	0.231	2.280	100%	0.020
P00004	Codon	Sto.Soft	11	0.120	0.241	2.026	100%	0.014
P00004	Codon	Sto.Hard	11	0.124	0.241	2.026	100%	0.010
P00004	Lagrangian	Det.Soft	11	0.290	1.575	6.718	100%	0.012
P00004	Lagrangian	Det.Hard	11	1.099	1.992	7.697	100%	0.012
P00004	Lagrangian	Sto.Soft	11	0.749	0.934	4.587	100%	0.006
P00004	Lagrangian	Sto.Hard	11	0.825	1.354	6.192	100%	0.010
P01308	Amino	Det.Soft	11	0.204	0.500	3.557	100%	0.011
P01308	Amino	Det.Hard	11	0.336	0.499	2.806	100%	0.011
P01308	Amino	Sto.Soft	11	0.250	0.338	2.816	100%	0.013
P01308	Amino	Sto.Hard	11	0.297	0.539	3.713	100%	0.013
P01308	Codon	Det.Soft	11	0.203	0.318	2.491	100%	0.014
P01308	Codon	Det.Hard	11	0.219	0.359	4.287	100%	0.023
P01308	Codon	Sto.Soft	11	0.312	0.572	4.458	100%	0.015
P01308	Codon	Sto.Hard	11	0.310	0.453	3.944	100%	0.017
P01308	Lagrangian	Det.Soft	11	0.649	1.797	7.258	100%	0.011
P01308	Lagrangian	Det.Hard	11	0.925	2.119	9.042	100%	0.007
P01308	Lagrangian	Sto.Soft	11	0.632	0.910	4.758	100%	0.009
P01308	Lagrangian	Sto.Hard	11	0.802	1.440	7.083	100%	0.009
P01825	Amino	Det.Soft	11	0.193	0.563	4.545	100%	0.016
P01825	Amino	Det.Hard	11	0.204	0.323	2.427	100%	0.012
P01825	Amino	Sto.Soft	11	0.393	0.787	4.853	100%	0.015
P01825	Amino	Sto.Hard	11	0.485	0.974	5.022	100%	0.018
P01825	Codon	Det.Soft	11	0.179	0.556	3.960	100%	0.015
P01825	Codon	Det.Hard	11	0.150	0.556	3.960	100%	0.015
P01825	Codon	Sto.Soft	11	0.170	0.865	3.574	100%	0.015
P01825	Codon	Sto.Hard	11	0.192	0.591	3.169	100%	0.013
P01825	Lagrangian	Det.Soft	11	0.556	1.642	6.998	100%	0.007
P01825	Lagrangian	Det.Hard	11	0.977	1.509	6.748	100%	0.010
P01825	Lagrangian	Sto.Soft	11	0.682	1.093	5.371	100%	0.011
P01825	Lagrangian	Sto.Hard	11	0.942	1.514	6.683	100%	0.009
P04637	Amino	Det.Soft	11	0.184	0.552	2.216	100%	0.007
P04637	Amino	Det.Hard	11	0.177	0.395	2.058	100%	0.007
P04637	Amino	Sto.Soft	11	0.223	0.498	3.245	100%	0.007
P04637	Amino	Sto.Hard	11	0.261	0.498	3.245	100%	0.005
P04637	Codon	Det.Soft	11	0.097	0.202	1.534	100%	0.004
P04637	Codon	Det.Hard	11	0.182	0.253	1.631	100%	0.005
P04637	Codon	Sto.Soft	11	0.164	0.508	3.216	100%	0.006
P04637	Codon	Sto.Hard	11	0.204	0.647	3.023	100%	0.014
P04637	Lagrangian	Det.Soft	11	0.786	1.497	6.369	100%	0.020
P04637	Lagrangian	Det.Hard	11	0.883	2.495	10.67	100%	0.009
P04637	Lagrangian	Sto.Soft	11	0.905	1.451	6.735	100%	0.009
P04637	Lagrangian	Sto.Hard	11	0.885	1.734	7.791	100%	0.009
P0CG48	Amino	Det.Soft	11	0.081	0.308	1.572	100%	0.007
P0CG48	Amino	Det.Hard	11	0.128	0.317	2.481	100%	0.007
P0CG48	Amino	Sto.Soft	11	0.143	0.353	1.931	100%	0.005
P0CG48	Amino	Sto.Hard	11	0.130	0.569	2.759	100%	0.008
P0CG48	Codon	Det.Soft	11	0.082	0.281	1.923	100%	0.006
P0CG48	Codon	Det.Hard	11	0.170	0.281	1.923	100%	0.006
P0CG48	Codon	Sto.Soft	11	0.140	0.347	3.245	100%	0.005
P0CG48	Codon	Sto.Hard	11	0.157	0.453	3.535	100%	0.005
P0CG48	Lagrangian	Det.Soft	11	0.342	1.523	6.522	100%	0.007
P0CG48	Lagrangian	Det.Hard	11	0.784	1.397	6.068	100%	0.009

Continued on next page

Table S25: Case-level full-matrix STE discrepancy diagnostics for all 144 protein-by-variant cases (continued).

Protein	Mechanism	Variant	Checkpoints	Δ_Θ med.	Δ_Θ max	Δ_S max	Coverage	Max ratio
P0CG48	Lagrangian	Sto.Soft	11	0.575	0.959	4.820	100%	0.006
P0CG48	Lagrangian	Sto.Hard	11	0.763	1.336	6.834	100%	0.011
P0DTC2	Amino	Det.Soft	11	0.175	0.414	2.177	100%	0.004
P0DTC2	Amino	Det.Hard	11	0.252	0.367	2.382	100%	0.005
P0DTC2	Amino	Sto.Soft	11	0.196	0.484	2.828	100%	0.005
P0DTC2	Amino	Sto.Hard	11	0.206	0.581	3.426	100%	0.004
P0DTC2	Codon	Det.Soft	11	0.188	0.329	2.881	100%	0.004
P0DTC2	Codon	Det.Hard	11	0.288	0.394	3.072	100%	0.006
P0DTC2	Codon	Sto.Soft	11	0.220	0.424	2.717	100%	0.004
P0DTC2	Codon	Sto.Hard	11	0.264	0.421	3.108	100%	0.005
P0DTC2	Lagrangian	Det.Soft	11	0.807	1.667	7.114	100%	0.009
P0DTC2	Lagrangian	Det.Hard	11	1.103	1.344	5.440	100%	0.009
P0DTC2	Lagrangian	Sto.Soft	11	0.743	0.915	4.555	100%	0.011
P0DTC2	Lagrangian	Sto.Hard	11	0.742	1.125	5.349	100%	0.009
P0DTC9	Amino	Det.Soft	11	0.075	1.380	5.830	100%	0.007
P0DTC9	Amino	Det.Hard	11	0.544	1.451	7.072	100%	0.017
P0DTC9	Amino	Sto.Soft	11	0.130	0.474	3.159	100%	0.009
P0DTC9	Amino	Sto.Hard	11	0.156	0.655	4.161	100%	0.010
P0DTC9	Codon	Det.Soft	11	0.107	1.250	7.716	100%	0.010
P0DTC9	Codon	Det.Hard	11	0.619	1.253	7.716	100%	0.014
P0DTC9	Codon	Sto.Soft	11	0.228	0.957	6.490	100%	0.013
P0DTC9	Codon	Sto.Hard	11	0.194	0.983	6.630	100%	0.008
P0DTC9	Lagrangian	Det.Soft	11	0.543	2.018	8.300	100%	0.008
P0DTC9	Lagrangian	Det.Hard	11	0.422	2.306	9.756	100%	0.010
P0DTC9	Lagrangian	Sto.Soft	11	0.739	1.049	4.667	100%	0.005
P0DTC9	Lagrangian	Sto.Hard	11	0.647	0.994	4.777	100%	0.005
P31417	Amino	Det.Soft	11	0.256	0.691	2.309	100%	0.013
P31417	Amino	Det.Hard	11	0.206	1.069	3.781	100%	0.020
P31417	Amino	Sto.Soft	11	0.227	0.658	3.282	100%	0.012
P31417	Amino	Sto.Hard	11	0.214	0.486	3.389	100%	0.012
P31417	Codon	Det.Soft	11	0.257	0.515	3.161	100%	0.014
P31417	Codon	Det.Hard	11	0.364	0.547	3.443	100%	0.029
P31417	Codon	Sto.Soft	11	0.242	0.451	3.912	100%	0.016
P31417	Codon	Sto.Hard	11	0.284	0.531	3.458	100%	0.014
P31417	Lagrangian	Det.Soft	11	0.690	1.596	6.831	100%	0.013
P31417	Lagrangian	Det.Hard	11	0.943	1.718	6.584	100%	0.010
P31417	Lagrangian	Sto.Soft	11	0.622	0.924	5.135	100%	0.007
P31417	Lagrangian	Sto.Hard	11	0.770	1.183	5.886	100%	0.013
P42212	Amino	Det.Soft	11	0.148	0.461	2.422	100%	0.009
P42212	Amino	Det.Hard	11	0.246	0.394	2.110	100%	0.009
P42212	Amino	Sto.Soft	11	0.296	0.691	3.928	100%	0.016
P42212	Amino	Sto.Hard	11	0.293	0.751	3.928	100%	0.012
P42212	Codon	Det.Soft	11	0.122	0.454	2.771	100%	0.011
P42212	Codon	Det.Hard	11	0.200	0.533	3.667	100%	0.010
P42212	Codon	Sto.Soft	11	0.183	0.637	4.404	100%	0.011
P42212	Codon	Sto.Hard	11	0.299	0.575	3.998	100%	0.018
P42212	Lagrangian	Det.Soft	11	0.564	1.517	6.445	100%	0.008
P42212	Lagrangian	Det.Hard	11	0.503	2.120	9.177	100%	0.011
P42212	Lagrangian	Sto.Soft	11	0.604	1.083	5.081	100%	0.006
P42212	Lagrangian	Sto.Hard	11	0.677	1.068	5.191	100%	0.008
P61626	Amino	Det.Soft	11	0.185	0.350	2.312	100%	0.012
P61626	Amino	Det.Hard	11	0.216	0.445	2.108	100%	0.010
P61626	Amino	Sto.Soft	11	0.168	0.624	4.212	100%	0.009
P61626	Amino	Sto.Hard	11	0.199	1.025	5.069	100%	0.016
P61626	Codon	Det.Soft	11	0.078	0.251	2.445	100%	0.011
P61626	Codon	Det.Hard	11	0.201	0.251	2.168	100%	0.009
P61626	Codon	Sto.Soft	11	0.141	0.683	5.638	100%	0.009
P61626	Codon	Sto.Hard	11	0.173	0.683	5.638	100%	0.011
P61626	Lagrangian	Det.Soft	11	0.891	1.905	8.113	100%	0.023
P61626	Lagrangian	Det.Hard	11	1.053	1.777	7.449	100%	0.007
P61626	Lagrangian	Sto.Soft	11	0.566	0.915	4.594	100%	0.011
P61626	Lagrangian	Sto.Hard	11	0.619	1.125	5.117	100%	0.012
P99999	Amino	Det.Soft	11	0.068	0.869	3.156	100%	0.010
P99999	Amino	Det.Hard	11	0.325	0.869	3.794	100%	0.021
P99999	Amino	Sto.Soft	11	0.122	0.365	2.476	100%	0.015

Continued on next page

Table S25: Case-level full-matrix STE discrepancy diagnostics for all 144 protein-by-variant cases (continued).

Protein	Mechanism	Variant	Checkpoints	Δ_Θ med.	Δ_Θ max	Δ_S max	Coverage	Max ratio
P99999	Amino	Sto.Hard	11	0.257	0.839	5.517	100%	0.022
P99999	Codon	Det.Soft	11	0.043	0.445	3.201	100%	0.025
P99999	Codon	Det.Hard	11	0.183	0.445	3.201	100%	0.025
P99999	Codon	Sto.Soft	11	0.124	0.409	2.940	100%	0.012
P99999	Codon	Sto.Hard	11	0.150	0.409	2.940	100%	0.011
P99999	Lagrangian	Det.Soft	11	0.649	1.845	7.885	100%	0.023
P99999	Lagrangian	Det.Hard	11	1.180	2.055	8.519	100%	0.009
P99999	Lagrangian	Sto.Soft	11	0.696	1.191	5.280	100%	0.015
P99999	Lagrangian	Sto.Hard	11	0.865	1.387	6.324	100%	0.014

S6.2. Constraint Mechanism Convergence Analysis

S6.2.1. Vectorized Transformation Functions

Before presenting constraint-specific convergence analysis, we define the vectorized transformation functions introduced in the main paper that convert codon probabilities to sequence representations.

Hard Mode Discretization (main paper Equation 7):

$$\text{Onehot}(\mathbf{P}_{\text{codon}}) = \{\mathcal{E}[\arg\max_{c\in\mathcal{C}(y_j)} P_{\text{codon}_{j,c}}]\}_{j=1}^{M} \tag{47}$$

This function selects the highest-probability codon at each position j and returns its one-hot encoding $\mathcal{E}[c]$, producing a discrete RNA sequence.

Soft Mode Reconstruction (main paper Equation 8):

$$R_{\text{nuc}}(\mathbf{P}_{\text{codon}}) = \{\sum_{c\in\mathcal{C}(y_j)} P_{\text{codon}_{j,c}} \cdot \mathcal{E}[c]\}_{j=1}^{M} \tag{48}$$

This function reconstructs nucleotide probabilities as weighted sums of codon encodings, producing a continuous probability distribution over nucleotides while preserving amino acid constraints.

Transformation Patterns by Constraint Type (main paper Table 2):

- **Codon Profile Constraint & Amino Matching Softmax:** Both use:
  - Soft mode: $T^{\text{soft}} = R_{\text{nuc}}(\mathbf{P}_{\text{codon}})$
  - Hard mode: $T^{\text{hard}} = \text{Onehot}(\mathbf{P}_{\text{codon}})$

  They differ in how $\mathbf{P}_{\text{codon}}$ is computed: via $\Pi_{\text{codon}}(\Theta_{\text{codon}})$ for Codon Profile versus $\Pi_{\text{amino}}(\Theta^{(j)})$ for Amino Matching.
- **Lagrangian Multiplier:** Uses:
  - Soft mode: $T^{\text{soft}} = \mathbf{P}$ (raw nucleotide probabilities)
  - Hard mode: $T^{\text{hard}} = \text{Onehot}(\Pi_{\text{amino}}(\mathbf{P}))$ (valid-codon conversion then discretization)

These unified transformation functions enable consistent analysis across all constraint mechanisms while respecting their distinct approaches to amino acid preservation.

S6.2.2. Codon Profile Convergence

Codon Profile operates in a reduced parameter space where amino acid sequences are guaranteed to be preserved throughout optimization.

Parameter Space (main paper Equation 1): $\Theta_{\text{codon}} = \{\theta_{j,c} : c \in \mathcal{C}(y_j), j \in [1, M]\}$ where

$$\dim(\Theta_{\text{codon}}) = \sum_{j=1}^{M} |\mathcal{C}(y_j)| \tag{49}$$

Theorem 6 (Codon Profile Convergence Properties) *Under Assumptions 1-3, Codon Profile-constrained optimization using parameter space Θ_{codon} (main paper Equation 1) and probability function Π_{codon} (main paper Equation 2) satisfies:*

1. ***Constraint Preservation:*** *By construction, codon probabilities $\mathbf{P}_{codon} = \Pi_{codon}(\Theta_{codon}) = softmax(\theta_{j,c}/\sigma)_{c\in\mathcal{C}(y_j)}$ operate only within valid codon space, automatically preserving amino acid identity at all positions j*
2. ***Enhanced Convergence:*** *$\lim_{t\to\infty} \|\nabla F_{Codon}(\Theta^{(t)}_{codon})\| = 0$ with potentially improved conditioning*

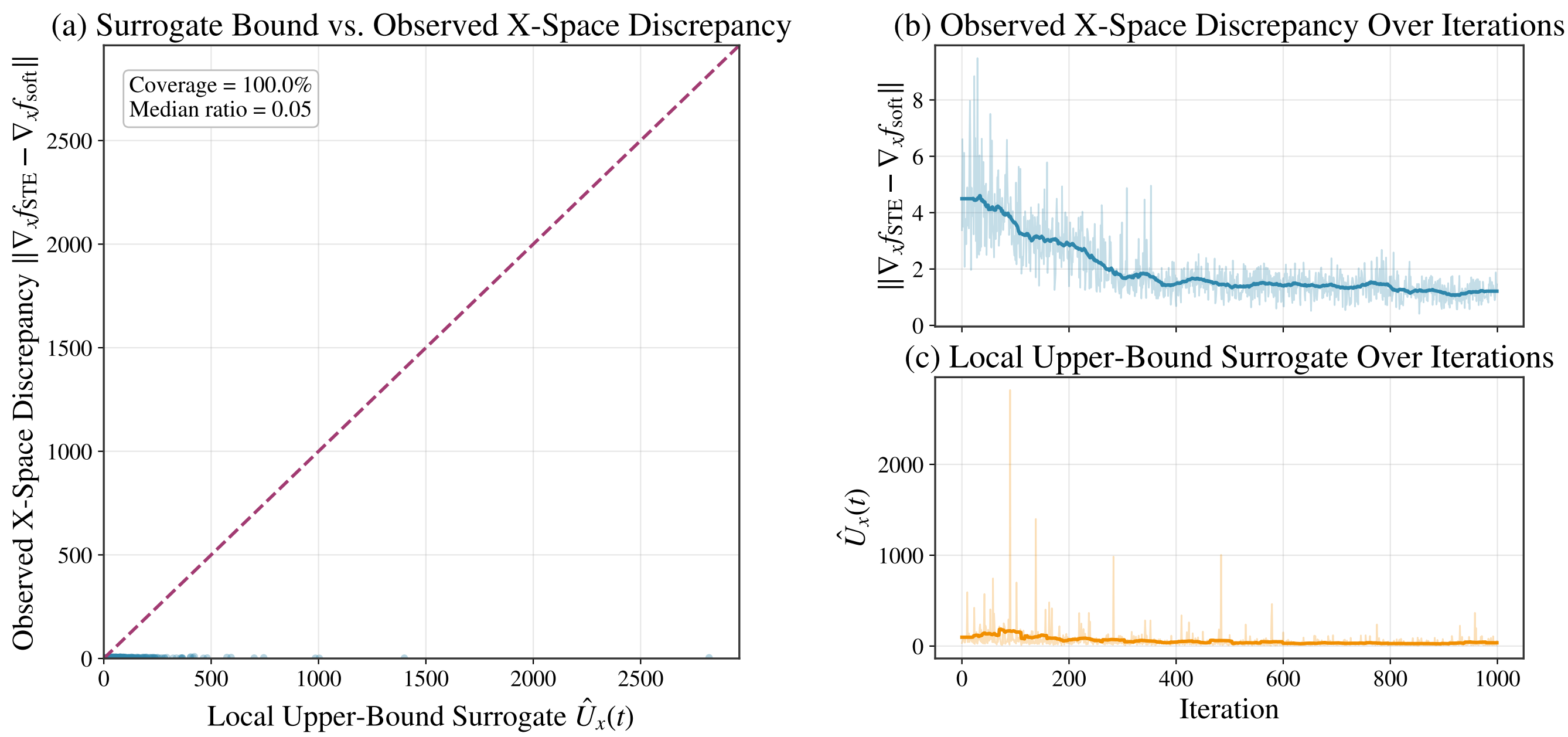

Fig. S6: Theory-motivated local upper-bound surrogate for the input-space STE discrepancy (O15263, Amino.Sto.Soft, 1000 iterations). **(a)** Observed input-space discrepancy $\|\nabla_{\mathbf{S}} f_{\text{model}}(\mathbf{S}_{\text{hard}}) - \nabla_{\mathbf{S}} f_{\text{model}}(\mathbf{S}_{\text{soft}})\|$ versus the local surrogate $\hat{U}_S(t)$; all points lie below the $y = x$ diagonal. **(b)** Observed input-space discrepancy over iterations. **(c)** Local upper-bound surrogate over iterations. The coverage is 100%, while the observed/surrogate ratio remains small (mean 0.064, median 0.054, maximum 0.396), indicating that the surrogate is conservative rather than tight.

Proof We establish both constraint preservation and enhanced convergence through dimensionality reduction.

Step 1: Automatic Constraint Satisfaction. By construction, Codon Profile uses the probability function Π_{codon} (main paper Equation 2):

$$\mathbf{P}_{\text{codon}} = \Pi_{\text{codon}}(\Theta_{\text{codon}}) = \text{softmax}(\theta_{j,c}/\sigma)_{c \in \mathcal{C}(y_j)} \tag{50}$$

The nucleotide probabilities are obtained via the soft mode transformation $T^{\text{soft}} = R_{\text{nuc}}(\mathbf{P}_{\text{codon}})$ (main paper Equation 8), which reconstructs nucleotide probabilities as weighted sums of codon encodings. Since all operations are restricted to valid codons $\mathcal{C}(y_j)$ for the target amino acid y_j, amino acid identity is preserved by construction.

Step 2: Reduced Parameter Space Analysis. The optimization operates in the reduced space $\mathbb{R}^{\sum_{j=1}^{M} |\mathcal{C}(y_j)|}$ (dimensionality of Θ_{codon}) instead of the full RNA parameter space $\mathbb{R}^{L \times 4}$ where $L = 3M$. For standard amino acids, $|\mathcal{C}(y_j)| \in \{1, 2, 3, 4, 6\}$, yielding an average dimensionality reduction by a factor of approximately 4, from $4L$ parameters to $\sum_{j=1}^{M} |\mathcal{C}(y_j)| \approx L$ parameters.

Step 3: Convergence Rate Improvement. The constraint eliminates infeasible directions from the gradient flow, potentially improving the condition number of the Hessian matrix. Specifically:

$$\kappa(H_{\text{Codon}}) \leq \kappa(H_{\text{full}}) \tag{51}$$

where $\kappa(\cdot)$ denotes the condition number. This improvement follows from the fact that constraining to a lower-dimensional manifold removes directions of high curvature that are orthogonal to the constraint manifold.

Therefore, convergence is enhanced while automatically maintaining constraint satisfaction. □

S6.2.3. Amino Matching Convergence

Amino Matching uses a valid-codon-restricted probability mapping that maintains differentiability while enforcing amino acid preservation through similarity-based codon selection.

Codon Probability Function (main paper Equation 3):

$$\mathbf{P}_{\text{codon}} = \Pi_{\text{amino}}(\Theta^{(j)})_c = \text{softmax}(\langle \Theta^{(j)}, \mathcal{E}[c] \rangle / \sigma)_{c \in \mathcal{C}(y_j)} \tag{52}$$

where $\Theta^{(j)} \in \mathbb{R}^{3 \times 4}$ is the RNA parameter matrix for the codon at amino acid position j, and $\mathcal{E}[c]$ is the one-hot encoding of codon c. The inner product $\langle \Theta^{(j)}, \mathcal{E}[c] \rangle$ measures similarity between RNA parameters and valid codons. The nucleotide probabilities are obtained via $T^{\text{soft}} = R_{\text{nuc}}(\mathbf{P}_{\text{codon}})$ or $T^{\text{hard}} = \text{Onehot}(\mathbf{P}_{\text{codon}})$ (main paper Table 2).

Theorem 7 (Amino Matching Convergence with Valid-Codon Softmax) *Under Assumptions 1-3, Amino Matching optimization using probability function Π_{amino} (main paper Equation 3) satisfies:*

1. ***Constraint Preservation:*** *Amino acid constraints are automatically satisfied by restricting codon probabilities to valid codons $\mathcal{C}(y_j)$*
2. ***Full Parameter Space:*** *Optimization occurs in full RNA parameter space $\mathbb{R}^{L\times 4}$ (where $L = 3M$) while maintaining feasibility*
3. ***Differentiable Valid-Codon Mapping:*** *The probability function Π_{amino} preserves gradient flow through inner product similarity*

Proof We establish the three key properties through analysis of the differentiable valid-codon probability mapping.

Step 1: Automatic Constraint Satisfaction. By construction, Amino Matching ensures that codon probabilities are generated only from valid codons through the probability function Π_{amino} (main paper Equation 3):

$$\mathbf{P}_{\text{codon}} = \Pi_{\text{amino}}(\Theta^{(j)})_c = \text{softmax}(\langle \Theta^{(j)}, \mathcal{E}[c]\rangle/\sigma)_{c\in\mathcal{C}(y_j)} \tag{53}$$

Since the softmax is computed only over valid codons $\mathcal{C}(y_j)$ for target amino acid y_j, amino acid constraints are automatically preserved. The transformation to nucleotide space via T^{soft} or T^{hard} (main paper Table 2) maintains this constraint preservation.

Step 2: Preservation of Gradient Flow. The probability function Π_{amino} maintains continuous and well-defined gradients. For the codon probability gradient:

$$\frac{\partial \Pi_{\text{amino}}(\Theta^{(j)})_c}{\partial \Theta^{(j)}} = \frac{\partial}{\partial \Theta^{(j)}} \left[\text{softmax}(\langle \Theta^{(j)}, \mathcal{E}[c]\rangle/\sigma)_{c\in\mathcal{C}(y_j)}\right] \tag{54}$$

The gradient exists everywhere and is Lipschitz continuous with respect to $\Theta^{(j)}$, ensuring stable optimization dynamics for the soft probability map. The soft transformation T^{soft} remains differentiable by the chain rule, while hard-mode updates use the STE surrogate described in Section S6 rather than relying on differentiability of the hard decoding map.

Step 3: Full Parameter Space Flexibility. Unlike Codon Profile, Amino Matching explores the complete RNA parameter space $\mathbb{R}^{L\times 4}$ (where $L = 3M$) while maintaining feasibility through the valid-codon probability function Π_{amino}. This provides optimization flexibility while preserving biological constraints, combining nucleotide-level parameterization with automatic constraint satisfaction through valid-codon-restricted softmax normalization. □

S6.2.4. Lagrangian Method Convergence

The Lagrangian approach [Boyd and Vandenberghe, 2004] uses penalty methods to enforce amino acid constraints through dual variables, with probabilistic codon selection for discrete sequence generation.

Lagrangian Formulation (main paper Equation 5):

$$\mathcal{L}_{\text{total}} = f_{\text{model}}(T(\Theta)) + \lambda \cdot \mathcal{C}(\mathbf{P}) \tag{55}$$

where $\lambda \geq 0$ is the Lagrange multiplier that controls penalty strength, and the constraint penalty (main paper Equation 4) measures amino acid constraint violation:

$$\mathcal{C}(\mathbf{P}) = \frac{1}{M}\sum_{j=1}^{M} \min_{c\in\mathcal{C}(y_j)} \|\mathbf{P}^{(j)} - \mathcal{E}[c]\|^2 \tag{56}$$

Here, $\mathbf{P}^{(j)}$ denotes the 3×4 probability matrix for the codon at amino acid position j, $\mathcal{C}(y_j)$ is the set of valid codons encoding amino acid y_j, and $\mathcal{E}[c]$ is the one-hot encoding of codon c.

The discrete sequence selection uses a probabilistic criterion:

$$\text{selected codon} = \arg\max_{c\in\mathcal{C}(y_j)} P(c|\mathbf{P}^{(j)}) \tag{57}$$

where $P(c|\mathbf{P}^{(j)})$ represents the probability of codon c given the probability distribution at position j.

Theorem 8 (Lagrangian Convergence with Standard Subgradient Method) *Under standard regularity conditions, the Lagrangian method with standard subgradient updates [Rockafellar, 1970] for λ and probabilistic codon selection converges to points satisfying the KKT conditions:*

$$\begin{aligned} \nabla F(\Theta^*) + \lambda^* \nabla \mathcal{C}(\mathbf{P}^*) &= 0 \\ \mathcal{C}(\mathbf{P}^*) &\leq \epsilon \end{aligned} \tag{58}$$

with convergence rate $O(1/\sqrt{N})$ for the multiplier λ.

Proof We establish convergence through standard subgradient analysis with probabilistic selection consistency.

Table S26. Unified Convergence Summary for ID3 Variants

Variant	Type	Convergence Target	Key Property
**Det.Soft**	Deterministic Soft	Exact critical point	Strongest guarantee
**Sto.Soft**	Stochastic Soft	Expected convergence	Exploration capability
**Det.Hard**	Deterministic Hard	$O(B_{\text{STE}})$-neighborhood	Discrete tracking
**Sto.Hard**	Stochastic Hard	$O(\sqrt{B_{\text{STE}}^2 + \sigma_G^2})$-neighborhood	Combined benefits

Step 1: Subgradient Method for λ Updates. Following the main paper (Equation 6), the Lagrange multiplier is updated using the standard subgradient method:

$$\begin{aligned} \lambda^{(t+1)} &= \max(0, \lambda^{(t)} + \eta_t \cdot g^{(t)}) \\ \eta_t &= \eta_0/\sqrt{t+1} \end{aligned} \tag{59}$$

where $g^{(t)}$ is the subgradient of the constraint at iteration t, η_t is the decaying step size (with η_0 being the initial step size), and the max operation ensures $\lambda \geq 0$.

The step size satisfies the Robbins-Monro conditions:

$$\sum_{t=1}^{\infty} \eta_t = \infty \quad \text{and} \quad \sum_{t=1}^{\infty} \eta_t^2 < \infty \tag{60}$$

Step 2: Convergence Rate Analysis. For the convex constraint penalty function $\mathcal{C}(\mathbf{P})$, the standard subgradient method achieves:

$$\min_{1 \leq t \leq N} \left| \mathcal{C}(\mathbf{P}^{(t)}) - \epsilon \right| \leq \frac{D_{\text{feas}}}{\sqrt{N}} \tag{61}$$

where D_{feas} is a constant depending on the diameter of the feasible region and the subgradient bound. This establishes the $O(1/\sqrt{N})$ convergence rate.

Step 3: Probabilistic Selection Consistency. The discrete sequence selection using $\arg\max_{c \in \mathcal{C}(y_j)} P(c|\mathbf{P}^{(j)})$ ensures that:

- The selected codon is always valid (satisfies amino acid constraints)
- The selection aligns with the continuous optimization objective
- As $\mathbf{P}^{(t)}$ converges, the discrete selection stabilizes

Step 4: KKT Conditions at Convergence. At convergence, with $\mathcal{C}(\mathbf{P}^*) \leq \epsilon$ and optimal λ^*, the stationarity condition holds:

$$\nabla F(\Theta^*) + \lambda^* \nabla \mathcal{C}(\mathbf{P}^*) = 0 \tag{62}$$

The combination of subgradient convergence for λ and gradient descent for Θ ensures convergence to a point satisfying the approximate KKT conditions with tolerance ϵ. □

S6.3. Convergence Summary and Hierarchy

S6.3.1. Convergence Hierarchy

S6.3.2. Practical Implications

Progressive Complexity: Each extension adds specific capabilities while maintaining convergence, but also introduces new practical considerations.

Temperature Tuning: Temperature affects two competing factors: lower σ typically brings T_{soft} closer to the hard decision, whereas the global softmax Jacobian bound scales like $1/\sigma$ and increases optimization sensitivity.

Variant Selection Guidelines:

- Use **Det.Soft** when model robustness is high and soft inputs are acceptable
- Use **Det.Hard** when discrete outputs are required and gradient bias is acceptable
- Use **Sto.Soft** when exploration is critical and noise robustness is available
- Use **Sto.Hard** when both exploration and discrete tracking are needed

S6.4. Conclusion of Convergence Analysis

This comprehensive convergence analysis establishes theoretical foundations for the ID3 framework across all variants and constraint mechanisms. The three constraint mechanisms (Codon Profile Constraint, Amino Matching Softmax, Lagrangian Multiplier) operate within the unified three-layer architecture while maintaining their distinct approaches to amino acid sequence preservation. The analysis reveals that:

1. All ID3 variants possess well-defined convergence properties under practical assumptions
2. Constraint mechanisms maintain convergence while providing biological sequence preservation
3. The choice of variant should be based on the trade-off between convergence quality and practical requirements
4. Pre-trained model quality fundamentally determines optimization success

These theoretical guarantees provide confidence for practical RNA sequence optimization applications while highlighting the importance of appropriate variant selection based on specific design requirements.

S7. CAI Reference Gene Set Construction

S7.1. Introduction and Rationale

The Codon Adaptation Index (CAI) serves as a fundamental metric for evaluating the translational efficiency of heterologous genes in bacterial expression systems. Traditional CAI calculations rely on genome-wide codon usage frequencies, which may not accurately reflect the codon preferences of highly expressed genes in specific expression systems. This section details the development and implementation of an expression-system-specific CAI reference set for *E. coli* BL21(DE3), the predominant strain used in recombinant protein production.

S7.2. Reference Gene Set Composition

The BL21(DE3) reference gene set was constructed using multiple data sources to ensure comprehensive coverage of highly expressed genes. The final reference set comprises **83 genes** across four functional categories, selected through automated GenBank extraction and validated for biological relevance to protein production.

S7.2.1. Ribosomal Proteins (54 genes)

All components of the 30S and 50S ribosomal subunits were included due to their direct role in protein synthesis and consistently high expression levels:

- **30S ribosomal proteins**: rpsA, rpsB, rpsC, rpsD, rpsE, rpsF, rpsG, rpsH, rpsI, rpsJ, rpsK, rpsL, rpsM, rpsN, rpsO, rpsP, rpsQ, rpsR, rpsS, rpsT, rpsU (21 genes)
- **50S ribosomal proteins**: rplA, rplB, rplC, rplD, rplE, rplF, rplI, rplJ, rplK, rplL, rplM, rplN, rplO, rplP, rplQ, rplR, rplS, rplT, rplU, rplV, rplW, rplX, rplY, rpmA, rpmB, rpmC, rpmD, rpmE, rpmF, rpmG, rpmH, rpmI, rpmJ (33 genes)

S7.2.2. Translation Factors (11 genes)

Essential components of the translation machinery were selected based on their critical roles in initiation, elongation, and termination:

- **Elongation factors**: fusA (EF-G), tufA (EF-Tu), tufB (EF-Tu), tsf (EF-Ts)
- **Initiation factors**: infA (IF-1), infB (IF-2), infC (IF-3)
- **Release factors**: prfA (RF-1), prfB (RF-2), prfC (RF-3), frr (ribosome recycling factor)

S7.2.3. Central Metabolism Enzymes (14 genes)

Key enzymes in glycolysis and associated pathways were included due to their high expression during exponential growth:

- **Primary glycolytic pathway**: pgi, pfkA, fbaA, tpiA, gapA, pgk, gpmA, eno, pykA
- **Associated enzymes**: aceE, aceF, lpdA (pyruvate dehydrogenase complex)
- **Alternative pathway components**: pykF, pfkB

S7.2.4. Additional Functional Genes (4 genes)

BL21(DE3)-specific genes important for metabolism and RNA processing:

- fbp (fructose-1,6-bisphosphatase)
- ppsA (phosphoenolpyruvate synthase)
- trmD (tRNA methyltransferase)
- truA (tRNA pseudouridine synthase)

S7.3. RSCU-Based CAI Calculation Methodology

S7.3.1. RSCU Calculation

For each amino acid family, Relative Synonymous Codon Usage (RSCU) [Sharp and Li, 1987] values are computed from reference sequences:

$$\text{RSCU}_{ij} = X_{ij} \times \frac{n_i}{\sum_k X_{ik}} \tag{63}$$

where X_{ij} is the count of codon j for amino acid i in reference sequences, n_i is the number of synonymous codons for amino acid i, and $\sum_k X_{ik}$ is the total count of all codons for amino acid i.

S7.3.2. Relative Adaptiveness Weights

CAI weights are derived from RSCU values. For each codon c encoding amino acid i:

$$w_c = \frac{\text{RSCU}_{ic}}{\max_{c' \in \mathcal{C}(i)}(\text{RSCU}_{ic'})} \tag{64}$$

where $\max_{c' \in \mathcal{C}(i)}(\text{RSCU}_{ic'})$ is the maximum RSCU value among all codons encoding amino acid i.

S7.3.3. CAI Definition

The Codon Adaptation Index (CAI) for a given sequence $S = (c_1, c_2, \ldots, c_M)$ is defined as the geometric mean of the relative adaptiveness values:

$$\mathrm{CAI}(S) = \left(\prod_{j=1}^{M} w_{c_j} \right)^{1/M} \tag{65}$$

where w_{c_j} is the relative adaptiveness weight for codon c_j at position j, and M is the total number of codons in the sequence. For numerical stability, this is commonly computed in log-space:

$$\mathrm{CAI}(S) = \exp\left(\frac{1}{M} \sum_{j=1}^{M} \ln(w_{c_j}) \right) \tag{66}$$

CAI values range from 0 to 1, with higher values indicating better adaptation to the host organism's codon usage preferences.

S7.3.4. Non-Synonymous Codon Exclusion

Critical for standard library consistency, the system excludes non-synonymous codons from CAI calculations:

- **Stop codons**: TAA, TAG, TGA (weight = 0.0)
- **Non-synonymous codons**: ATG (methionine), TGG (tryptophan) are excluded from CAI calculation

S7.4. Validation and Comparison

S7.4.1. Standard Library Compatibility

The implementation achieves perfect consistency with the Python CAI library through:

- Identical RSCU calculation using the same reference sequence methodology
- Consistent codon exclusion matching non-synonymous and stop codon handling
- Numerical precision maintaining floating-point consistency in geometric mean calculations
- 0% calculation difference with Python CAI library across 96 test sequences

S7.5. Complete Codon Weight Tables

The complete 64-codon weight matrices for the BL21(DE3) reference system are available in the supplementary data files. A representative subset is shown below:

Table S27. Representative codon weights for BL21(DE3) reference set

Amino Acid	Codon	RSCU	Weight (w_i)
Lysine	AAA	1.52	1.000
Lysine	AAG	0.48	0.316
Leucine	CTG	3.12	1.000
Leucine	CTT	0.65	0.208
Arginine	CGT	2.45	1.000
Arginine	CGC	1.89	0.771
Arginine	CGA	0.23	0.094

The BL21(DE3) reference set provides strain-specific codon optimization targets that better reflect the translation machinery preferences in this widely-used expression system, enabling more accurate CAI calculations for recombinant protein production applications.

S8. Binary Search for CAI-Aware Discretization

S8.1. Problem Setup and Motivation

S8.1.1. The CAI-Constrained Optimization Problem

The CAI-aware discretization function (main paper Equation 17) seeks to find the most probable codon sequence under the accessibility-optimized distribution while maintaining sufficient codon adaptation:

$$\begin{aligned} \Psi_{\mathrm{CAI}}^{\mathrm{hard}}(\mathbf{P}_{\mathrm{codon}}) &= \arg\max_{S} P(S|\mathbf{P}_{\mathrm{codon}}) \\ \text{s.t.} \quad \mathrm{CAI}(S) &\geq \text{threshold} \end{aligned} \tag{67}$$

where S is the discrete codon sequence, $\mathbf{P}_{\text{codon}}$ is the accessibility-optimized codon probability distribution from the model, and $P(S|\mathbf{P}_{\text{codon}}) = \prod_{j=1}^{M} P_{\text{codon}_{j,S_j}}$ represents the likelihood of sequence S under this distribution. The CAI threshold threshold is set based on organism-specific requirements (e.g., 0.8 for *E. coli*).

S8.1.2. Computational Challenge

This optimization problem is computationally intractable in its general form:

- **Search Space**: The full combinatorial codon space contains $\prod_{j=1}^{M} |\mathcal{C}(y_j)|$ possible sequences, where $\mathcal{C}(y_j)$ is the codon set for amino acid y_j at position j
- **Constraint Complexity**: The CAI constraint is non-linear and couples decisions across all positions
- **Greedy Limitations**: Position-wise greedy selection (choosing $\arg\max_c P_{\text{codon}_{j,c}}$ at each position) often violates the CAI constraint

To address this computational challenge, we employ a subspace reduction strategy that restricts the search to a one-dimensional parametric curve. Specifically, we linearly interpolate between two extreme strategies: (1) maximizing sequence probability under the accessibility-optimized distribution $\mathbf{P}_{\text{codon}}$, and (2) maximizing CAI using organism-specific codon frequencies. This dimensional reduction makes the optimization problem tractable while preserving the essential trade-off between accessibility optimization and codon adaptation, as formalized in the following subsection.

S8.2. Linear Interpolation Strategy

S8.2.1. Interpolation Formulation

We define a parametric family of probability distributions that interpolates between the accessibility-optimized distribution $\mathbf{P}_{\text{codon}}$ and the organism-specific codon usage frequencies $\mathbf{w}$ (main paper Equation 18):

$$\mathbf{P}(\gamma) = \gamma \cdot \mathbf{w} + (1-\gamma) \cdot \mathbf{P}_{\text{codon}} \tag{68}$$

where:

- $\mathbf{w} = [w_{j,c}]$ is the normalized codon usage frequency vector for the target organism
- $\mathbf{P}_{\text{codon}} = [P_{\text{codon}_{j,c}}]$ is the accessibility-optimized distribution from the model
- $\gamma \in [0,1]$ is the interpolation parameter controlling the trade-off:
  - $\gamma = 0$: Pure accessibility optimization (may have low CAI)
  - $\gamma = 1$: Pure CAI optimization (CAI = 1, but may have low model probability)

The reduced optimization problem becomes finding the minimal γ^* that satisfies the CAI constraint:

$$\gamma^* = \min\{\gamma \in [0,1] : \text{CAI}(\arg\max \mathbf{P}(\gamma)) \geq \text{threshold}\} \tag{69}$$

where $\arg\max \mathbf{P}(\gamma)$ denotes the sequence obtained by selecting the highest-probability codon at each position under distribution $\mathbf{P}(\gamma)$.

S8.2.2. Theoretical Guarantees

The linear interpolation strategy provides strong theoretical guarantees within the one-dimensional search space.

Theorem 9 (Linear Interpolation Properties) *The linear interpolation strategy satisfies the following properties within the one-dimensional search space $\mathbf{P}(\gamma) = \gamma \cdot \mathbf{w} + (1-\gamma) \cdot \mathbf{P}_{codon}$ for $\gamma \in [0,1]$:*

1. ***Piecewise Monotonicity:*** *CAI$(\arg\max \mathbf{P}(\gamma))$ is piecewise monotonically non-decreasing in γ*
2. ***Subspace Global Optimality:*** *The solution γ^* is globally optimal within the one-dimensional interpolation subspace*
3. ***Logarithmic Convergence:*** *Binary search achieves $O(\log M)$ convergence on at most M switching events*

Proof We establish each property through rigorous mathematical analysis.

Property 1: Piecewise Monotonicity.

For each amino acid position j, the selected codon is determined by:

$$c_j^*(\gamma) = \arg\max_{c \in \mathcal{C}(y_j)} P_{j,c}(\gamma) = \arg\max_{c \in \mathcal{C}(y_j)} [\gamma w_{j,c} + (1-\gamma) P_{\text{codon}_{j,c}}] \tag{70}$$

Consider two codons $c_{\text{acc}}, c_{\text{CAI}} \in \mathcal{C}(y_j)$ where:

- c_{acc} is the accessibility-optimal codon: $P_{\text{codon}_{j,c_{\text{acc}}}} > P_{\text{codon}_{j,c_{\text{CAI}}}}$
- c_{CAI} is the CAI-optimal codon: $w_{j,c_{\text{CAI}}} > w_{j,c_{\text{acc}}}$

Define the score difference:

$$\Delta(\gamma) = [\gamma w_{j,c_{\text{CAI}}} + (1-\gamma)P_{\text{codon}_{j,c_{\text{CAI}}}}] - [\gamma w_{j,c_{\text{acc}}} + (1-\gamma)P_{\text{codon}_{j,c_{\text{acc}}}}] \tag{71}$$

This simplifies to:

$$\Delta(\gamma) = \gamma(w_{j,c_{\text{CAI}}} - w_{j,c_{\text{acc}}}) + (1-\gamma)(P_{\text{codon}_{j,c_{\text{CAI}}}} - P_{\text{codon}_{j,c_{\text{acc}}}}) \tag{72}$$

By our definitions, we have:

- $\Delta(0) = P_{\text{codon}_{j,c_{\text{CAI}}}} - P_{\text{codon}_{j,c_{\text{acc}}}} < 0$ (accessibility-optimal codon wins at $\gamma = 0$)
- $\Delta(1) = w_{j,c_{\text{CAI}}} - w_{j,c_{\text{acc}}} > 0$ (CAI-optimal codon wins at $\gamma = 1$)

By the Intermediate Value Theorem, there exists a unique switching point $\gamma_j^{\text{switch}} \in (0,1)$ where codon selection switches from c_{acc} to c_{CAI}. The derivative $\frac{d\Delta}{d\gamma} = (w_{j,c_{\text{CAI}}} - w_{j,c_{\text{acc}}}) - (P_{\text{codon}_{j,c_{\text{CAI}}}} - P_{\text{codon}_{j,c_{\text{acc}}}}) > 0$ confirms this is the only switching point.

Solving $\Delta(\gamma_j^{\text{switch}}) = 0$ yields:

$$\gamma_j^{\text{switch}} = \frac{P_{\text{codon}_{j,c_{\text{acc}}}} - P_{\text{codon}_{j,c_{\text{CAI}}}}}{(w_{j,c_{\text{CAI}}} - w_{j,c_{\text{acc}}}) + (P_{\text{codon}_{j,c_{\text{acc}}}} - P_{\text{codon}_{j,c_{\text{CAI}}}})} \tag{73}$$

The CAI function for the selected sequence is:

$$\text{CAI}(\gamma) = \exp\left(\frac{1}{M}\sum_{j=1}^{M}\ln(w_{c_j^*(\gamma)})\right) \tag{74}$$

Between switching points, codon selection remains constant, making CAI constant. At each switching point γ_j^{switch}, position j switches from the accessibility-optimal codon to the CAI-optimal codon (which has higher weight by definition), causing:

$$\lim_{\gamma \to (\gamma_j^{\text{switch}})^+} \text{CAI}(\gamma) \geq \lim_{\gamma \to (\gamma_j^{\text{switch}})^-} \text{CAI}(\gamma) \tag{75}$$

Therefore, $\text{CAI}(\gamma)$ is piecewise monotonically non-decreasing on $[0,1]$.

Property 2: Subspace Global Optimality.

The feasible region is:

$$\mathcal{F} = \{\gamma \in [0,1] : \text{CAI}(\mathbf{P}(\gamma)) \geq \text{threshold}\} \tag{76}$$

By piecewise monotonicity (Property 1), if $\gamma \in \mathcal{F}$, then for any $\gamma' \geq \gamma$, we have $\text{CAI}(\gamma') \geq \text{CAI}(\gamma) \geq \text{threshold}$, implying $\gamma' \in \mathcal{F}$. Therefore, $\mathcal{F} = [\gamma^*, 1]$ where $\gamma^* = \inf \mathcal{F}$ (assuming feasibility).

The optimization problem within the interpolation subspace is:

$$\min_{\gamma \in [0,1]} \gamma \quad \text{subject to} \quad \text{CAI}(\mathbf{P}(\gamma)) \geq \text{threshold} \tag{77}$$

Since the objective $f(\gamma) = \gamma$ is linear (hence convex) and the feasible region $\mathcal{F} = [\gamma^*, 1]$ is convex, the global optimum within the subspace is γ^*, which minimizes γ (maximizes consistency with accessibility-optimized distribution) while satisfying the CAI constraint.

Property 3: Logarithmic Convergence.

Let $\mathcal{G} = \{0 = \gamma_0 < \gamma_1 < \cdots < \gamma_K = 1\}$ be the set of all switching points (at most M switching events, one per position). The binary search operates on this discrete set.

At each iteration, the search interval is halved. To locate γ^* within tolerance ε, we need:

$$\frac{1}{2^k} \leq \varepsilon \Rightarrow k \geq \log_2(1/\varepsilon) \tag{78}$$

For discrete search on at most M switching events, the complexity is $O(\log M)$ iterations. □

Remark 1 (Search Space Limitations) *The binary search algorithm explores only a one-dimensional subspace of the full combinatorial codon space. Specifically:*

- ***Full Space Size:*** $\prod_{j=1}^{M} |\mathcal{C}(y_j)|$ *possible codon combinations*
- ***Explored Space:*** *The 1D parametric curve* $\mathbf{P}(\gamma)$ *for* $\gamma \in [0,1]$
- ***Reachable Codons:*** *At position* j*, only codons with* $w_{j,c} > 0$ *OR* $P_{codon_{j,c}} > 0$ *can be selected*
- ***Potential Gap:*** *Codon combinations outside the interpolation path are not explored*

While this linear interpolation captures the primary accessibility-CAI trade-off axis, alternative codon combinations outside this path may exist. The algorithm provides a computationally efficient approach with strong guarantees within the explored subspace, representing a pragmatic balance between theoretical optimality and practical tractability.

Algorithm 1 Binary Search for CAI-Aware Discretization

Require: Codon distribution $\mathbf{P}_{\text{codon}}$, codon usage frequencies $\mathbf{w}$, CAI threshold τ, tolerance $\varepsilon = 10^{-6}$, max iterations $K = 50$
Ensure: Discrete sequence S^* with $\text{CAI}(S^*) \geq \tau$

$\gamma_{\text{lo}} \leftarrow 0$, $\gamma_{\text{hi}} \leftarrow 1$
$S_0 \leftarrow \text{Decode}(\mathbf{P}_{\text{codon}})$ ▷ Greedy argmax at $\gamma = 0$
if $\text{CAI}(S_0) \geq \tau$ **then**
    return S_0 ▷ Already satisfies constraint
end if
for $k = 1, \ldots, K$ **do**
    $\gamma_{\text{mid}} \leftarrow (\gamma_{\text{lo}} + \gamma_{\text{hi}})/2$
    $\mathbf{P}(\gamma_{\text{mid}}) \leftarrow \gamma_{\text{mid}} \cdot \mathbf{w} + (1 - \gamma_{\text{mid}}) \cdot \mathbf{P}_{\text{codon}}$
    $S_{\text{mid}} \leftarrow \text{Decode}(\mathbf{P}(\gamma_{\text{mid}}))$ ▷ Greedy argmax per position
    if $\text{CAI}(S_{\text{mid}}) \geq \tau$ **then**
        $\gamma_{\text{hi}} \leftarrow \gamma_{\text{mid}}$, $S^* \leftarrow S_{\text{mid}}$
    else
        $\gamma_{\text{lo}} \leftarrow \gamma_{\text{mid}}$
    end if
    if $\gamma_{\text{hi}} - \gamma_{\text{lo}} < \varepsilon$ **then**
        break
    end if
end for
return S^*

S8.3. Implementation

The binary search algorithm exploits the piecewise monotonicity property (Theorem 9, Property 1) to efficiently find the minimal γ^* satisfying the CAI constraint. Algorithm 1 provides the complete pseudocode.

To enhance diversity and explore sequences beyond the interpolation subspace, we additionally employ a greedy refinement strategy with hash-based duplicate detection. This combination ensures both theoretical optimality within the interpolation subspace and practical sequence diversity.

S9. CAI Integration: Two Orthogonal Dimensions

This section describes the two orthogonal dimensions of CAI integration in the ID3 framework. The first dimension is *CAI-aware discretization*, the base evaluation/discretization-stage CAI mechanism used in all CAI experiments: a binary search-based method that finds discrete sequences satisfying CAI constraints by interpolating between accessibility-optimal and CAI-optimal codon distributions. The second dimension is the optional *penalty-based joint loss* (applied during the optimization phase): a multi-objective loss that penalizes deviation from CAI targets during gradient descent. In Table S2, the no-penalty rows use the base CAI-aware discretization mechanism alone, whereas the +Penalty rows add the joint-loss term during optimization. These two dimensions are therefore independent and can be combined. Statistical performance comparison is provided in Section S2.2.6.

S9.1. Two Implementation Dimensions

CAI-aware Discretization (base mechanism; applied during evaluation/discretization). This approach focuses solely on accessibility during gradient optimization:

$$\min_{\Theta} \mathcal{L}_{\text{Access}}(T(\Theta)) \tag{79}$$

CAI targets are then achieved through binary search interpolation between accessibility-optimal and CAI-optimal codon distributions during the discretization phase (detailed in Section S8). This base mechanism separates accessibility optimization from CAI adjustment and is used in all CAI experiments reported in this paper.

Penalty-Based Joint Loss (optional, applied during optimization). This approach incorporates CAI constraints directly into the optimization objective through a unified loss function:

$$\mathcal{L}_{\text{total}} = \mathcal{L}_{\text{access}} + \lambda_{\text{CAI}} \cdot \mathcal{L}_{\text{CAI}} \tag{80}$$

where $\mathcal{L}_{\text{CAI}}(\Theta) = (\text{ECAI}(T(\Theta)) - \text{CAI}_{\text{target}})^2$ is computed using the Expected CAI. This approach works with both soft mode (continuous probability distributions) and hard mode (discrete sequences), making it the only option for CAI optimization in soft discretization mode. In all cases, the decoded discrete sequence is still produced through the base CAI-aware discretization mechanism at evaluation time; the penalty term is the optional mechanism by which CAI also influences the optimization trajectory itself. For hard mode, penalty-based joint loss can therefore be combined with CAI-aware discretization (described in the previous subsection) for enhanced CAI control. The penalty coefficient λ_{CAI} is updated using the same subgradient method as the Lagrangian multiplier in the Lagrangian constraint mechanism (main paper Equation 6), enabling adaptive penalty strength adjustment during optimization.

Expected CAI (ECAI): For the penalty-based optimization approach, we define the Expected CAI that supports both continuous probability sequences and discrete one-hot sequences:

$$\text{ECAI}(\mathbf{P}_{\text{codon}}) = \left(\prod_{j=1}^{M} \sum_{c \in \mathcal{C}(y_j)} P_{\text{codon}_{j,c}} \cdot w_c \right)^{1/M} \tag{81}$$

where $\mathbf{P}_{\text{codon}} = (P_{\text{codon}_1}, P_{\text{codon}_2}, \ldots, P_{\text{codon}_M})$ represents either continuous probability distributions or discrete one-hot sequences at each position j, $\mathcal{C}(y_j)$ is the set of valid codons for amino acid y_j, and w_c is the CAI weight for codon c. This unified formulation enables ECAI computation from both probabilistic constraint outputs and discrete sequences, supporting gradient-based optimization of the penalty term.

S9.2. Performance Equivalence and Design Choice

Complete results for both approaches across 12 variants and 12 proteins are presented in Tables S2–S3, with detailed statistical comparison in Section S2.2.6. The use of penalty-based optimization during training shows minimal performance difference compared to training without penalty (mean difference = 0.005 kcal/mol, p = 0.661, Cohen's d = 0.012). Notably, the best-performing variant (Amino.Sto.Hard +Penalty, rank 1 in Table S2) does employ the penalty-based approach during optimization.

The strong performance of CAI-aware discretization with hard mode can be attributed to two key factors. First, hard discretization enables the pre-trained DeepRaccess model to operate on discrete sequences matching its training distribution, avoiding the distribution shift inherent in soft probability inputs. Second, the binary search allows precise CAI targeting without the gradient noise and optimization conflicts introduced by multi-objective penalty terms during training. These technical advantages explain why hard mode variants dominate the top rankings in Table S2, with CAI-aware discretization achieving the optimal balance between accessibility optimization and CAI constraint satisfaction.

S10. UTR Template Specifications

The ID3 framework employs fixed 5' and 3' UTR sequences derived from the pET-11a expression vector (Novagen), optimized for T7 RNA polymerase-based protein expression in *E. coli* [Studier and Moffatt, 1986].

5' UTR (70 nucleotides):

```
GGGAATTGTGAGCGGATAACAATTCCCCTCTAGAAATAATTTTGTTTAACTTTAAGAAGGAGATATACAT
```

3' UTR (63 nucleotides):

`GATCCGGCTGCTAACAAAGCCCGAAAGGAAGCTGAGTTGGCTGCTGCCACCGCTGAGCAATAA`

Vector details: `https://www.snapgene.com/plasmids/pet_and_duet_vectors_(novagen)/pET-11a`

References

Y. Bengio, N. Léonard, and A. Courville. Estimating or propagating gradients through stochastic neurons for conditional computation. *arXiv preprint arXiv:1308.3432*, 2013.

S. Boyd and L. Vandenberghe. *Convex Optimization*. Cambridge University Press, Cambridge, UK, 2004. doi: 10.1017/CBO9780511804441.

N. Dai, T. Zhou, W. Y. Tang, D. H. Mathews, and L. Huang. EnsembleDesign: messenger rna design minimizing ensemble free energy via probabilistic lattice parsing. *Bioinformatics*, 41(Supplement_1):i391–i400, 2025. doi: 10.1093/bioinformatics/btaf245.

I. Goodfellow, Y. Bengio, and A. Courville. *Deep Learning*. MIT Press, 2016. URL `http://www.deeplearningbook.org`.

S. Holm. A simple sequentially rejective multiple test procedure. *Scandinavian Journal of Statistics*, 6(2):65–70, 1979.

E. Jang, S. Gu, and B. Poole. Categorical reparameterization with Gumbel-Softmax. In *International Conference on Learning Representations (ICLR 2017)*. OpenReview. net, 2017.

N. Killoran, L. J. Lee, A. Delong, D. Duvenaud, and B. J. Frey. Generating and designing dna with deep generative models. arXiv preprint arXiv:1712.06148, 2017.

R. K. Krueger and M. Ward. JAX-RNAfold: scalable differentiable folding. *Bioinformatics*, 41(5):btaf203, 04 2025. doi: 10.1093/bioinformatics/btaf203.

J. Linder and G. Seelig. Fast activation maximization for molecular sequence design. *BMC Bioinformatics*, 22(1):510, 2021. ISSN 1471-2105. doi: 10.1186/s12859-021-04437-5.

Y. Nesterov. *Introductory Lectures on Convex Optimization: A Basic Course*, volume 87 of *Applied Optimization*. Springer, 2004. doi: 10.1007/978-1-4419-8853-9.

R. T. Rockafellar. *Convex Analysis*, volume 28 of *Princeton Mathematical Series*. Princeton University Press, Princeton, NJ, 1970.

P. M. Sharp and W. H. Li. The codon adaptation index-a measure of directional synonymous codon usage bias, and its potential applications. *Nucleic Acids Research*, 15(3):1281–1295, 1987.

F. W. Studier and B. A. Moffatt. Use of bacteriophage t7 rna polymerase to direct selective high-level expression of cloned genes. *Journal of Molecular Biology*, 189(1):113–130, 1986.
